# Geotemporal disparities in hip fractures burden among individuals aged ≥55 years (1990–2021) with projections to 2050

**DOI:** 10.3389/fpubh.2025.1600452

**Published:** 2025-09-01

**Authors:** Yuan Gu, Yingqi Chen, Wenzhao Li, Ningning Cheng, Songyun Deng, Yuan Ma, Daorong Xu, Jikun Qian

**Affiliations:** ^1^Division of Orthopaedics and Traumatology, Department of Orthopaedics, Nanfang Hospital, Southern Medical University, Guangzhou, China; ^2^Department of Orthopedics, The Second Xiangya Hospital, Central South University, Changsha, China; ^3^Department of Obstetrics, The First Affiliated Hospital, Guangzhou University of Chinese Medicine, Guangzhou, China; ^4^Department of Orthopaedics and Traumatology, Zhujiang Hospital, Southern Medical University, Guangzhou, China; ^5^Division of Orthopaedics and Traumatology, Department of Orthopaedics, The Seventh Affiliated Hospital, Southern Medical University, Foshan, China

**Keywords:** hip fractures, global burden of disease, incidence, prevalence, years lived with disability, trend analysis

## Abstract

**Background:**

Hip fractures (HFs) are common among older adults and represent a major cause of long-term functional impairment. The lack of up-to-date epidemiological data hinders the development of effective public health policies. This study investigates trends in HFs among individuals aged ≥55 years (HFs (≥55 years)), providing essential evidence to inform future prevention strategies.

**Methods:**

Using Global Burden of Disease Study 2021 data, we analyzed the age-standardized incidence rate (ASIR), prevalence rate (ASPR), and years lived with disability (YLDs) rate (ASYR), along with their trends, driving factors, age-sex-time patterns, health outcomes efficiency, and projections up to 2050.

**Results:**

In 2021, the global ASIR, ASPR, and ASYR of HFs (≥55 years) were 1,027.46 (95% UI: 719.73–1416.07), 2,037.39 (95% UI: 1,670.75–2475.71) per 100,000, and 185.49 (95% UI: 125.69–259.43) per 100,000 person-years, respectively. From 1990 to 2021, global ASIR and ASPR showed an overall upward trend, whereas ASYR declined (ASIR: AAPC = 0.20, 95% CI: 0.12–0.28; ASPR: AAPC = 0.31, 95% CI: 0.27–0.36; ASYR: AAPC = −0.43, 95% CI: −0.50 – −0.36). These trends are expected to persist by 2050, with ASIR reaching 1,102.66 (95% CI: 101.40–2,142.83), ASPR 2,052.14 (95% CI: 141.30–4,112.55) per 100,000, and ASYR declining to 174.43 (95% CI: 0–365.91) per 100,000 person-years. Significant disparities existed across 204 countries and territories. High SDI region bore a greater burden, though their growth rate had slowed, whereas Low SDI region showed a gradual increase from a lower baseline. Health inequalities were more pronounced in High SDI region, which also had the greatest potential for burden reduction. Population growth and aging were the primary drivers of these trends, with falls remaining the predominant cause. Notably, the burden increased more markedly among males.

**Conclusion:**

The global burden of HFs (≥55 years) is rising, underscoring the need to account for the complex distribution across populations and regions. Effective, targeted prevention and treatment strategies are essential to mitigating the disease burden and improving patient outcomes.

## 1 Introduction

Hip fractures (HFs) are common, high-impact orthopedic injuries that occur predominantly in older adults (1, 2). Beyond the fracture itself, HFs are frequently complicated by deep vein thrombosis, pressure ulcers, and cognitive decline that accelerate functional dependence and reduced quality of life (3, 4). Surgical treatment, rehabilitation, and disability-linked productivity losses impose substantial direct and indirect costs on patients, caregivers, and health systems (5, 6). With accelerating global population growth and aging, the burden of HFs is expected to become a serious challenge (7, 8).

Although previous studies have explored the epidemiological characteristics of HFs, most relied on data collected up to 2019 and were limited in geographic scope (9, 10). In the Global Burden of Disease 2021 (GBD 2021) study, not all HFs are classified as osteoporotic fractures. Nevertheless, prior literature and clinical guidelines commonly use HFs among individuals aged ≥55 years (HFs (≥55 years)) as a surrogate indicator for estimating the burden of osteoporosis and osteoporosis-related fractures (9, 11, 12). To specifically evaluate the burden of HFs among older adults, we utilized GBD 2021 data to systematically assess temporal trends and key determinants of HFs (≥55 years) across regions and populations. These findings aimed to provide evidence to optimize public health resource allocation and inform targeted health intervention policies.

## 2 Methods

### 2.1 Data source

The GBD 2021 provides estimates for 371 diseases and injuries, as well as 87 risk factors, across 204 countries and territories from 1990 to 2021 (13). We obtained data on the incidence, prevalence, and years lived with disability (YLDs) associated with HFs at the global, regional, and national levels from the GBD database for the period 1990–2021. All data used in this study are publicly available and can be accessed via the following URL: http://ghdx.healthdata.org/gbd-results-tool. This study was approved by the Ethics Committee for Human Studies of the Second Xiangya Hospital, Central South University (2023JJ40833).

### 2.2 Statistical analysis

The incidence, prevalence, and YLDs estimates for HFs (≥55 years) were presented as absolute numbers and age-standardized rates (ASR) per 100,000 population, along with their 95% uncertainty intervals (UIs), and were stratified by age group, sex, five sociodemographic index (SDI) regions, and 21 GBD regions. HFs (≥55 years) were categorized into 9 age groups: 55–59, 60–64, 65–69, 70–74, 75–79, 80–84, 85–89, 90–94, and 95 years and older. The ASR for each age group were computed using global age-standardized population data sourced from the GBD database. The formula for calculating the ASR of HFs (≥55 years) is as follows:


$$\frac{\sum_{i=1}^{n}(r_{i}*p_{i})}{100}$$

Where:

$r_{i}$ represents the age-specific rate for the i-th age group, where the age-specific rate is defined as the number of events in the age group divided by the population in that group. $p_{i}$ denotes the population size (or weight) for the corresponding age group in the GBD standard population, and n is the total number of age groups.

The SDI is a composite measure that integrates education, economic, and fertility levels, classified into five levels based on SDI quintiles: Low, Low-middle, Middle, High-middle, and High. It ranges from 0 (lowest) to 1 (highest) (14). Pearson correlation was used to assess the linear relationship between SDI and the burden of HFs (≥55 years), and Local Weighted Regression was applied to explore potential non-linear associations.

### 2.3 Joinpoint regression analysis

A log-linear model was used to analyze temporal trends in the age-standardized incidence rate (ASIR), prevalence rate (ASPR), and years lived with disability rate (ASYR) for HFs (≥55 years). The key outcomes included trend turning points (joint points), annual percentage change (APC), and average annual percentage change (AAPC), with 95% confidence intervals (CIs). APC reflects the yearly change in disease burden, while AAPC represents the average change over the entire study period (1990–2021) (15, 16).

### 2.4 Age-period-cohort model

We employed the Age-Period-Cohort model to analyze the disease burden over different time spans, focusing on age, period, and birth cohort effects. The Age-Period-Cohort model is expressed by the following formula: *Y_i,j,k_* = *α_i_* + *β_j_* + *γ_k_* + *ϵ_ijk_*.

Where 𝑌_𝑖,𝑗,𝑘_ represents the observed value for the 𝑖-th age group, the 𝑗-th period, and the 𝑘-th birth cohort. 𝛼_𝑖_ represents the age effect. 𝛽_𝑗_ is the period effect. 𝛾_𝑘_ is the birth cohort effect. 𝜖_𝑖𝑗𝑘_ is the error term, accounting for unexplained random fluctuations in the data. This methodology provides a comprehensive understanding of disease dynamics from demographic, sociological, and epidemiological perspectives (17).

### 2.5 Decomposition analysis

In our study, decomposition analysis was conducted to identify the main factors driving changes in the burden of HFs (≥55 years) from 1990 to 2021. It quantified the contributions of aging, population, and epidemiological changes. Epidemiological changes reflect changes in disease management, medical interventions, and overall health status. The black dots represent the total change values attributed to the three components, and the contribution of each factor to the overall burden change is illustrated using color-coded bar charts. Positive values indicate that the factor contributed to an increase in the disease burden, while negative values denote a corresponding reduction (18).

### 2.6 Predictive analysis

In the predictive analysis, we employed the Bayesian age-period-cohort (BAPC) model to examine the age, period, and cohort effects in the disease burden of HFs (≥55 years). This model was also used to forecast future health burdens. The BAPC model utilizes Bayesian inference by incorporating prior distributions to model the data; as new data are incorporated, the model iteratively updates the posterior distribution, progressively enhancing the accuracy of its predictions (19).

### 2.7 Health inequality analysis

The main purpose of health inequality analysis is to assess and quantify health disparities among different socioeconomic groups. Slope index and concentration index are used to assess the relationship between health inequality and SDI. The slope index measures the difference in health burden between the poorest and wealthiest groups, with a higher value indicating greater inequality. The concentration index reflects the distribution of disease burden across socioeconomic groups (16).

### 2.8 Frontier analysis

Frontier analysis was employed to evaluate healthcare efficiency and optimize the allocation of resources. In this part, we used Data Envelopment Analysis, enhanced with bootstrap resampling techniques, to reduce data bias and improve the robustness of the results. We constructed a nonlinear frontier, representing the “optimal boundary” defined by the minimum achievable burden across different SDI countries and territories. Countries and territories positioned on the frontier indicate that they have successfully minimized the disease burden with the same level of resource input. Frontier analysis facilitates the evaluation and comparison of health output efficiency across different countries and territories under identical resource constraints (20).

### 2.9 Statistics

All statistical analyses and data visualizations were conducted using R programming (version 4.4.1). In the trend analysis, *p* < 0.05 was considered statistically significant. For descriptive statistics, results are presented as means with 95% UI or 95% CI.

## 3 Results

### 3.1 Global burden of HFs (≥55 years)

**Figure 1:**
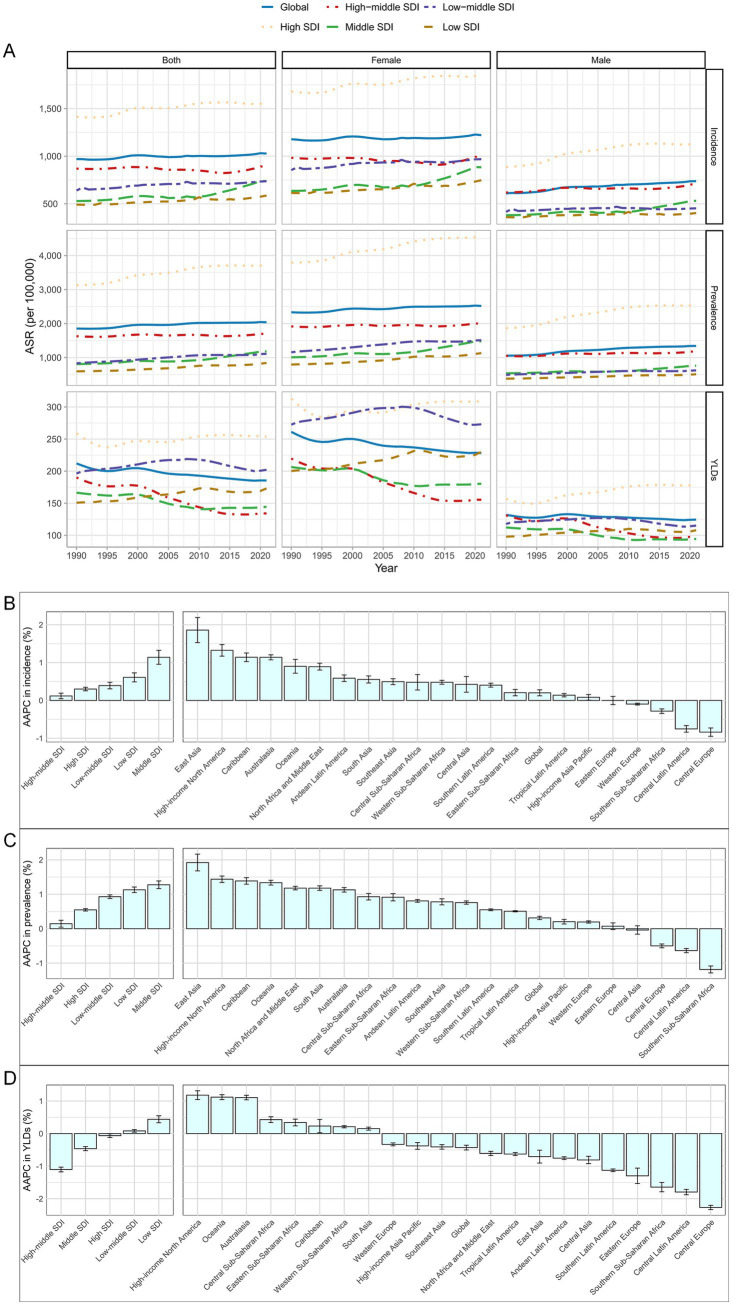
Global burden of HFs (≥55 years). **(A)** ASIR, ASPR, and ASYR for HFs (≥55 years) from 1990 to 2021, by sex and SDI. AAPC analysis of ASIR **(B)**, ASPR **(C)**, and ASYR **(D)** for HFs (≥55 years), from 1990 to 2021, by global, 5 SDI regions, and 21 GBD regions.

In 2021, the global ASIR and ASPR of HFs (≥55 years) reached 1,027.46 (95% UI: 719.73–1,416.07) and 2,037.39 (95% UI: 1,670.75–2,475.71) per 100,000, respectively, reflecting notable increases from 1990 levels. By contrast, the ASYR declined slightly, from 212.08 (95% UI: 144.85–293.83) in 1990 to 185.49 (95% UI: 125.69–259.43) per 100,000 person-years in 2021 (Figure 1A; Supplementary Table S1). The absolute burden also rose substantially, with females consistently bearing a significantly higher burden than males throughout 1990–2021 (Supplementary Table S2; Supplementary Figure S1A). Overall, the burden of HFs (≥55 years) has increased over the past 32 years; however, encouragingly, the associated disability burden has shown signs of improvement (Figures 1B–D).

### 3.2 Regional burden of HFs (≥55 years)

In 2021, the ASIR, ASPR, and ASYR in the High SDI region were 1,545.45 (95% UI: 1,090.09–2,115.88), 3,690.83 (95% UI: 3,029.71–4,474.90) per 100,000, and 253.65 (95% UI: 167.45–362.28) per 100,000 person-years, respectively. These values were the highest among the five SDI regions. In contrast, the lowest ASIR and ASPR were observed in the Low SDI region, while the lowest ASYR appeared in the High-middle SDI region (Figure 1A; Supplementary Table S1). In terms of absolute burden, the High SDI region reported the largest number of new cases, the highest prevalence, and the most YLDs attributable to HFs (≥55 years) (Supplementary Table S2; Supplementary Figure S1A). From 1990 to 2021, ASIR and ASPR rose in all SDI regions, most notably in the Middle SDI region. ASYR declined overall but increased in the Low SDI region (Figures 1B–D).

**Figure 2:**
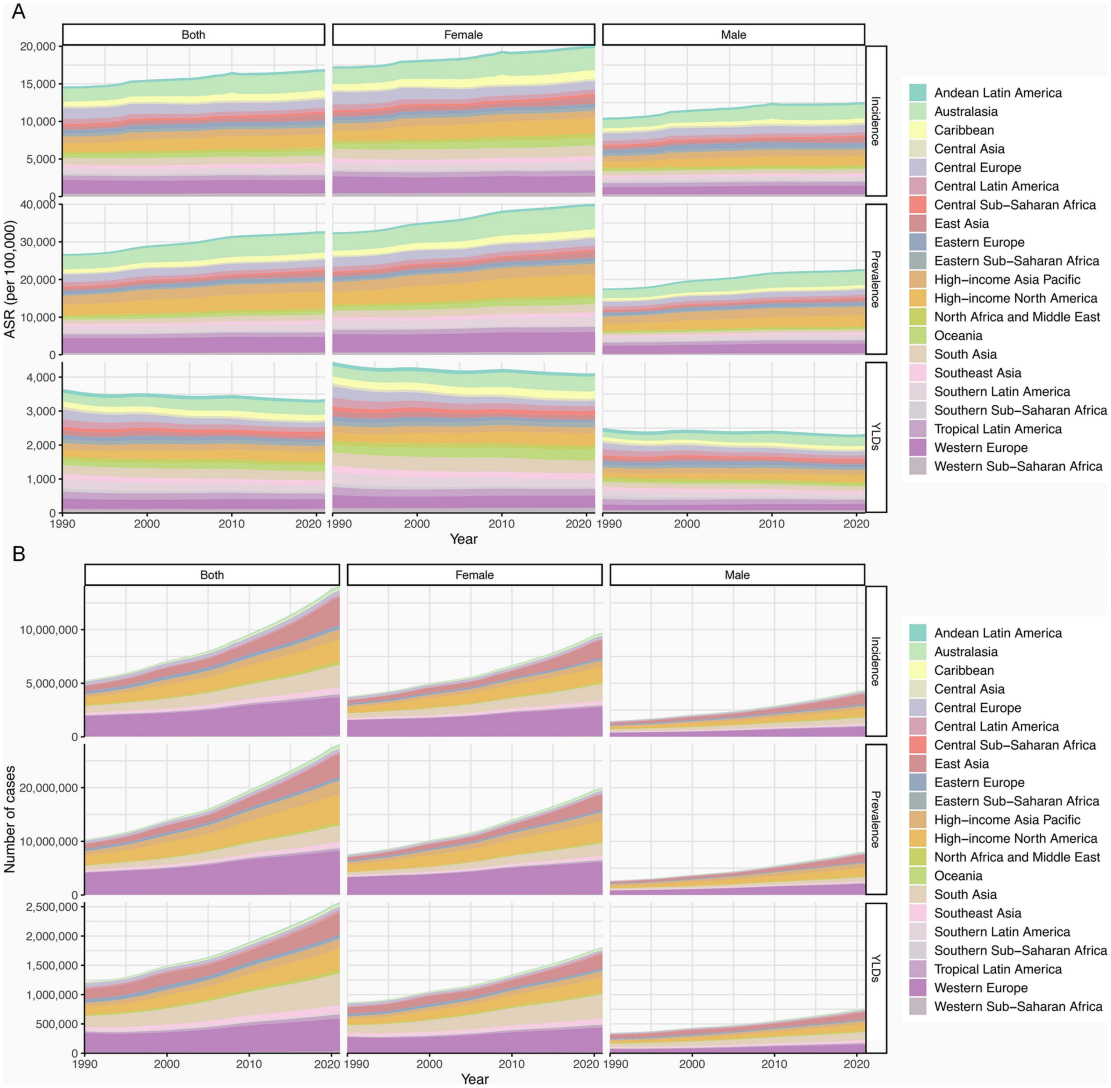
Regional burden of HFs (≥55 years). **(A)** ASIR, ASPR, and ASYR for HFs (≥55 years) from 1990 to 2021, by sex and 21 GBD regions. **(B)** New cases, prevalence count, and YLDs for HFs (≥55 years) from 1990 to 2021, by sex and 21 GBD regions.

As of 2021, the burden of HFs (≥55 years) was highest in high-income GBD regions such as Australasia, while it remained lowest in low-income regions like Southern Sub-Saharan Africa (Figure 2A; Supplementary Table S1). In terms of absolute burden, high-income regions reported substantially more new cases, prevalent cases, and YLDs—up to 20 times greater than those in low-income regions (Figure 2B; Supplementary Table S2). Notably, sex disparities were also more pronounced in high-income regions (Figure 2A; Supplementary Table S1). From 1990 to 2021, ASIR and ASPR rose in most 21 GBD regions, notably in East Asia. Meanwhile, ASYR showed an overall decline, with the steepest drop in Central Europe (Figures 1B–D).

### 3.3 National and regional burden of HFs (≥55 years)

**Figure 3:**
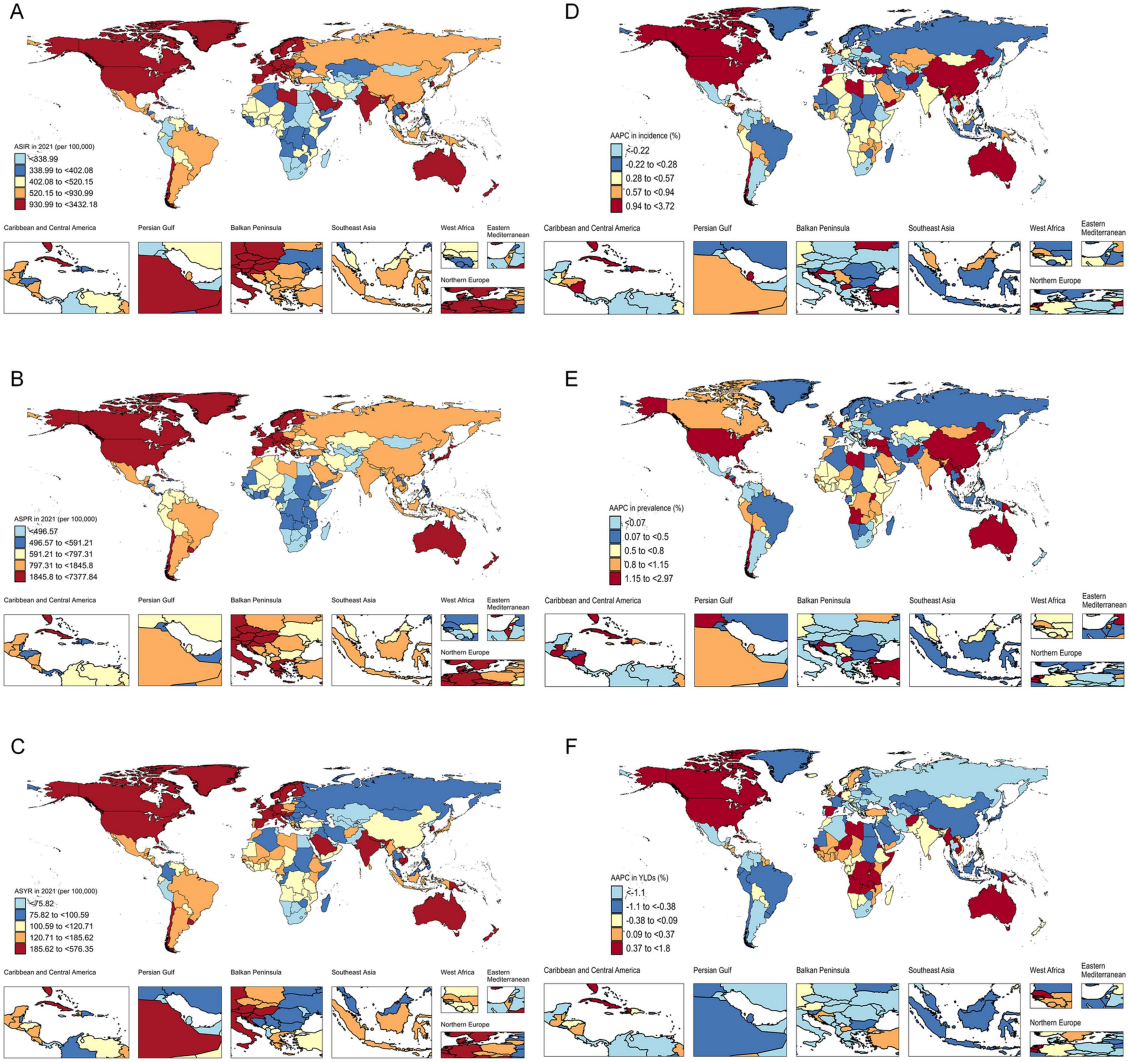
National and regional burden of HFs (≥55 years). ASIR **(A)**, ASPR **(B)**, and ASYR **(C)** for HFs (≥55 years) in 204 countries and territories in 2021. AAPC analysis results of ASIR **(D)**, ASPR **(E)**, and ASYR **(F)** for HFs (≥55 years) in 204 countries and territories from 1990 to 2021.

In 2021, the burden of HFs (≥55 years) varied widely across 204 countries and territories. Andorra reported the highest ASIR and ASPR at 3,432.18 (95% UI: 2,390.22–4,652.60) and 7,377.84 (95% UI: 6,169.25–8,749.68) per 100,000, respectively, while Greenland had the highest ASYR at 576.35 (95% UI: 391.84–797.67) per 100,000 person-years. Conversely, Bangladesh, Kiribati, and Kuwait exhibited the lowest ASIR, ASPR, and ASYR, respectively (Figures 3A–C; Supplementary Table S3). In absolute counts, China, the United States, and India led in new cases, prevalence, and YLDs (Supplementary Table S4; Supplementary Figures S2A–C). From 1990 to 2021, ASIR rose significantly in 136 countries (largest increase in Libya), ASPR increased in 160 (greatest rise in Bhutan), and ASYR declined in 105 (most notable drop in Lebanon) (Figures 3D–F).

### 3.4 Age-sex-time trends in HFs (≥55 years) burden

**Figure 4:**
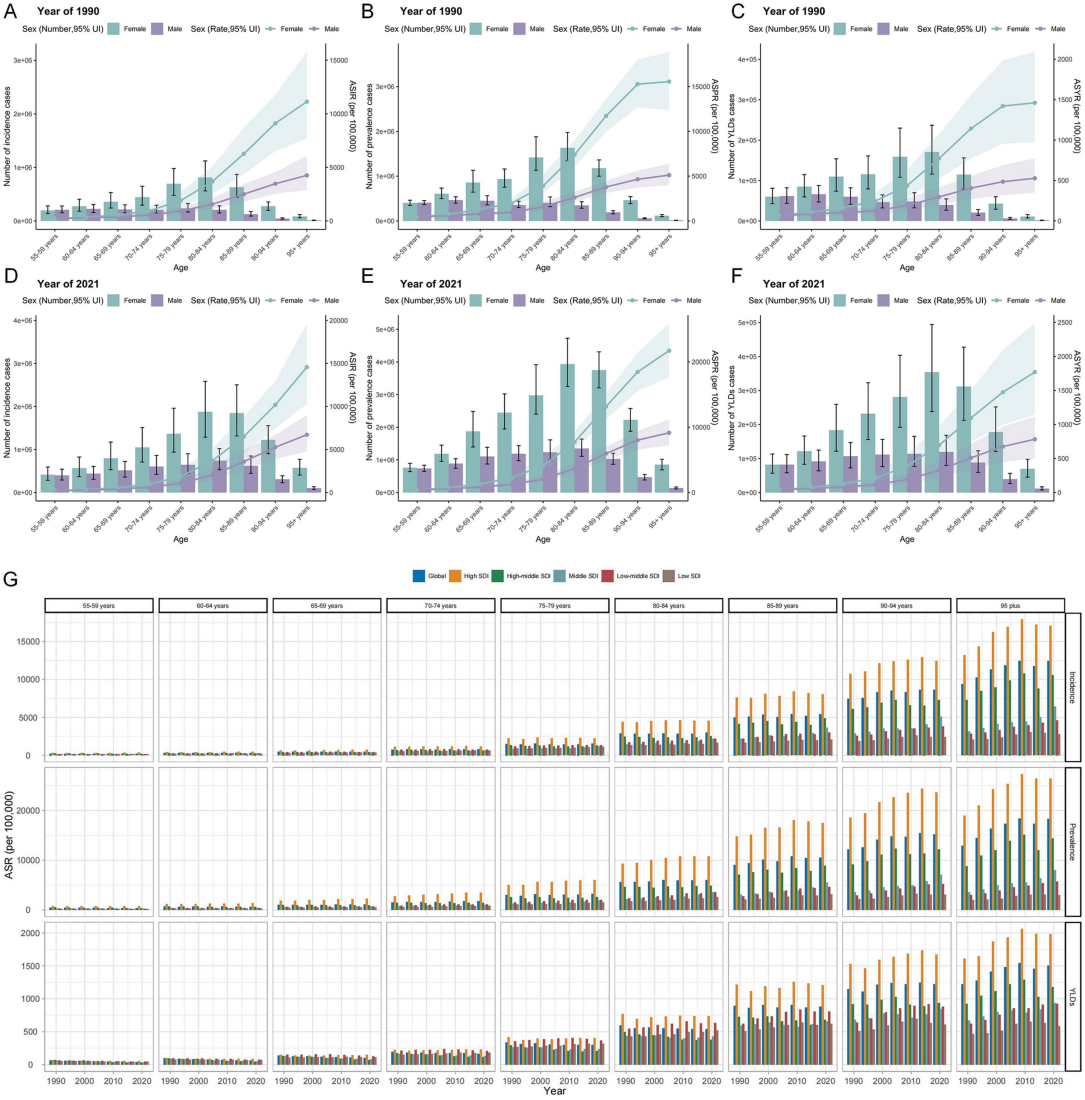
Age-sex-time trends in HFs (≥55 years) burden. Age-sex trends in new cases and ASIR **(A)**, prevalence count and ASPR **(B)**, and YLDs and ASYR **(C)** for HFs (≥55 years) globally in 1990. Age-sex trends in new cases and ASIR **(D)**, prevalence count and ASPR **(E)**, and YLDs and ASYR **(F)** for HFs (≥55 years) globally in 2021. **(G)** Age-time trends in ASIR, ASPR, and ASYR for HFs (≥55 years) from 1990 to 2021, by 5 SDI regions.

In 2021, the global ASIR, ASPR, and ASYR of HFs (≥55 years) increased with age, with the steepest rise observed in those aged ≥70 years—a pattern consistent with that of 1990 (Figures 4A–F). Sex–time analysis revealed a steady increase in ASIR and ASPR for both sexes across all SDI regions. Notably, ASYR trends differed markedly between sexes, varying across SDI levels (Figure 1A). Age–time analysis showed minimal regional differences among those aged 55–70 years. However, for individuals aged ≥70 years, the burden was highest and rising fastest in the High SDI region. In contrast, Low-middle and Low SDI regions, despite lower baseline levels, showed a sustained upward trajectory (Figure 4G). Trends in absolute burden mirrored those in ASR metrics (Supplementary Figure S3A).

### 3.5 Results of the joinpoint regression analysis for HFs (≥55 years)

**Figure 5:**
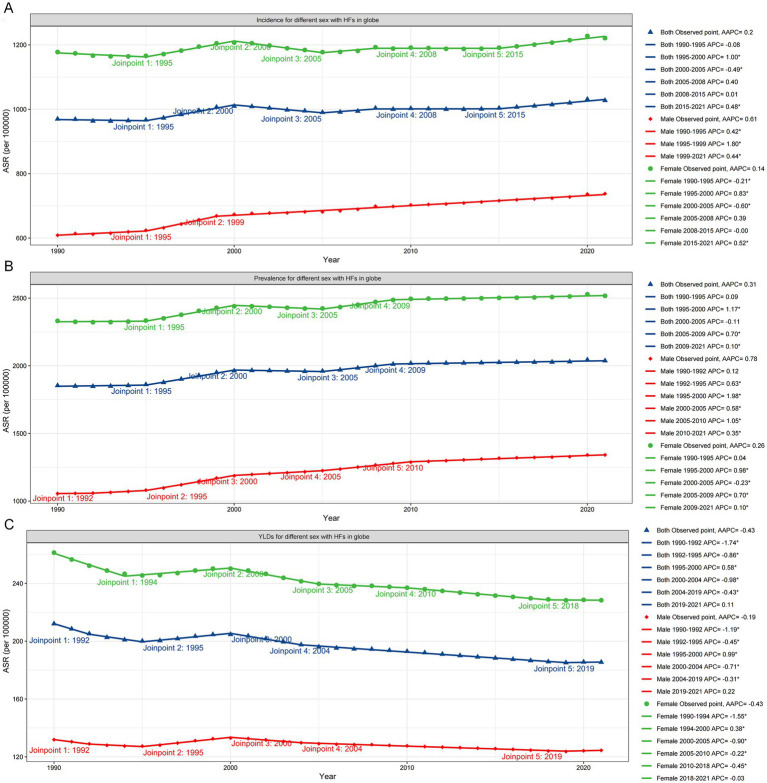
Joinpoint regression analysis for HFs (≥55 years) burden. Joinpoint regression analysis results of ASIR **(A)**, ASPR **(B)**, and ASYR **(C)** for global HFs (≥55 years), by sex.

From 1990 to 2021, the global ASIR and ASPR of HFs (≥55 years) showed overall upward trends (ASIR: AAPC = 0.20, 95% CI: 0.12–0.28; ASPR: AAPC = 0.31, 95% CI: 0.27–0.36), while ASYR declined steadily (AAPC = −0.43, 95% CI: −0.50 to −0.36). Although females consistently carried a higher burden, males experienced a more rapid increase and showed less improvement in disability. Further analysis revealed that all three ASRs exhibited distinct temporal fluctuations over the past 32 years: ASIR rose notably during 1995–2000 and 2015–2021; ASPR increased most rapidly between 1995–2000 and 2005–2009; and ASYR declined most significantly between 1990 and 2004 (Figures 5A–C; Supplementary Table S5).

### 3.6 Relationship between HFs (≥55 years) burden and SDI

**Figure 6:**
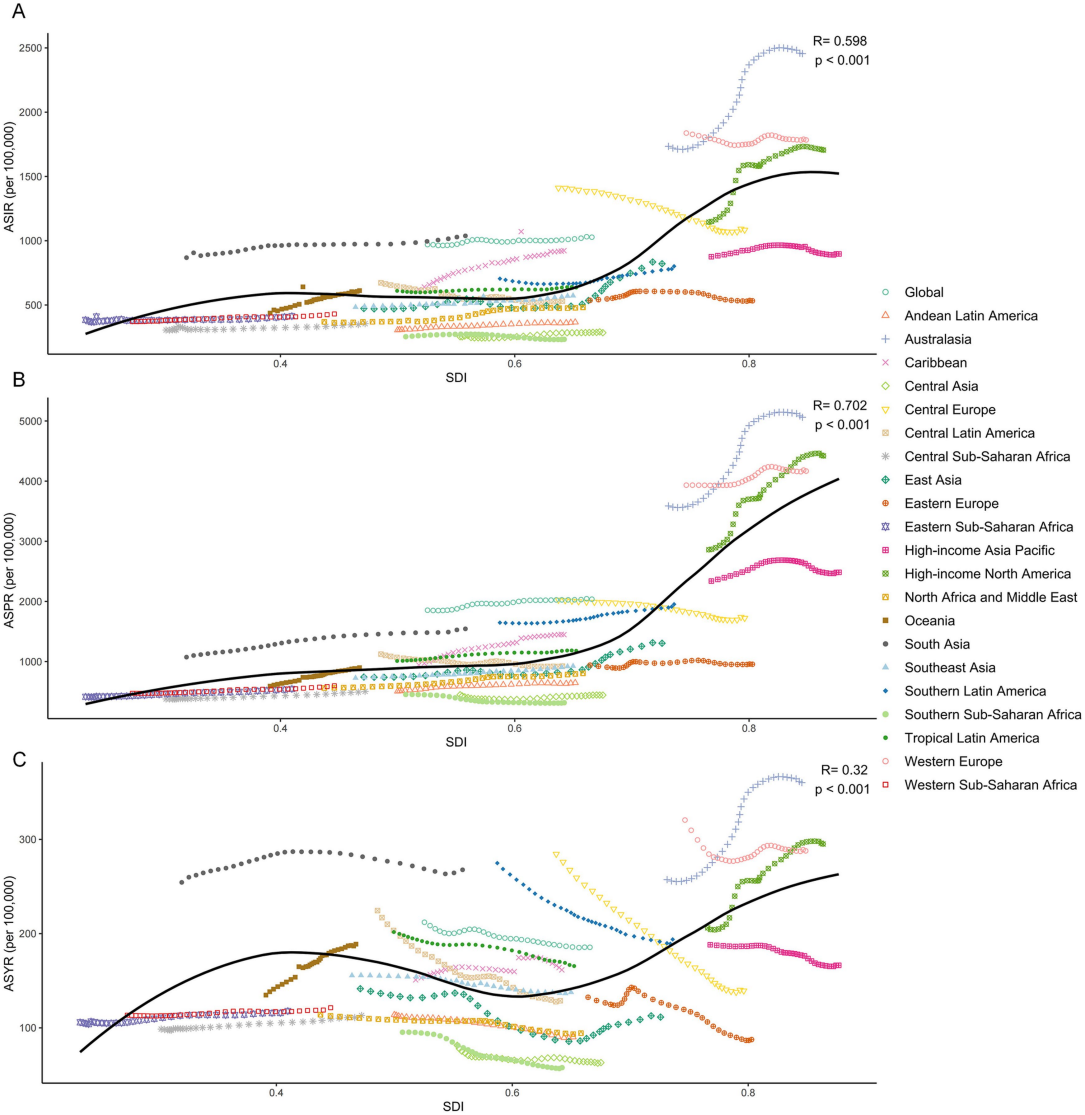
Relationship between HFs (≥55 years) burden and SDI. Relationship between ASIR **(A)**, ASPR **(B)**, and ASYR **(C)** for HFs (≥55 years) and SDI, by global and 21 GBD regions.

Globally and across the 21 GBD regions, the relationship between SDI and the ASIR, ASPR, and ASYR of HFs (≥55 years) was non-linear. Overall, the burden increased with SDI, with the strongest correlation observed for ASPR (*R* = 0.702, *p* < 0.001), followed by ASIR (*R* = 0.598, *p* < 0.001), and ASYR (*R* = 0.320, *p* < 0.001) (Figures 6A–C). Among 204 countries and territories in 2021, the association between SDI and ASRs was weak below an SDI of 0.75, but strengthened significantly above this threshold. In contrast, the inflection point in 1990 occurred at an SDI of 0.5. Similar trends were observed across all five SDI regions (Supplementary Figures S4A–L).

### 3.7 Results of the age-period-cohort analysis for HFs (≥55 years)

**Figure 7:**
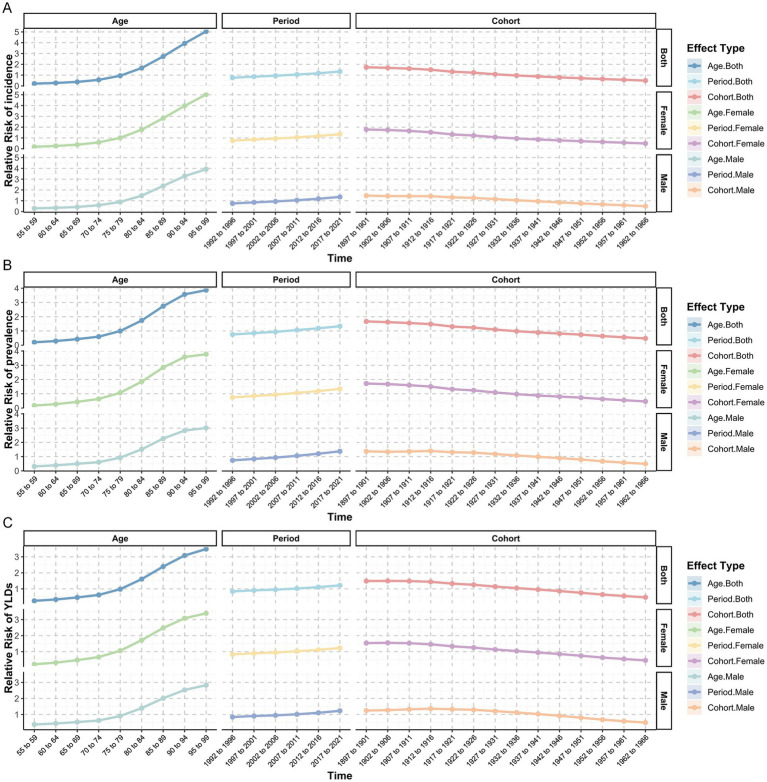
Age-Period-Cohort analysis for HFs (≥55 years) burden. Age-period-cohort analysis results for relative risk of incidence **(A)**, prevalence **(B)**, and YLDs **(C)** for HFs (≥55 years), by sex.

The age effect analysis revealed that the relative risks for the incidence, prevalence, and YLDs of HFs (≥55 years) rose with aging, particularly among individuals aged 70 and older. Period effect analysis indicated a modest upward trend in relative risks from 1990 to 2021. Cohort effect analysis revealed that relative risks across birth cohorts remained generally stable (Figures 7A–C).

### 3.8 Results of the decomposition analysis for HFs (≥55 years)

**Figure 8:**
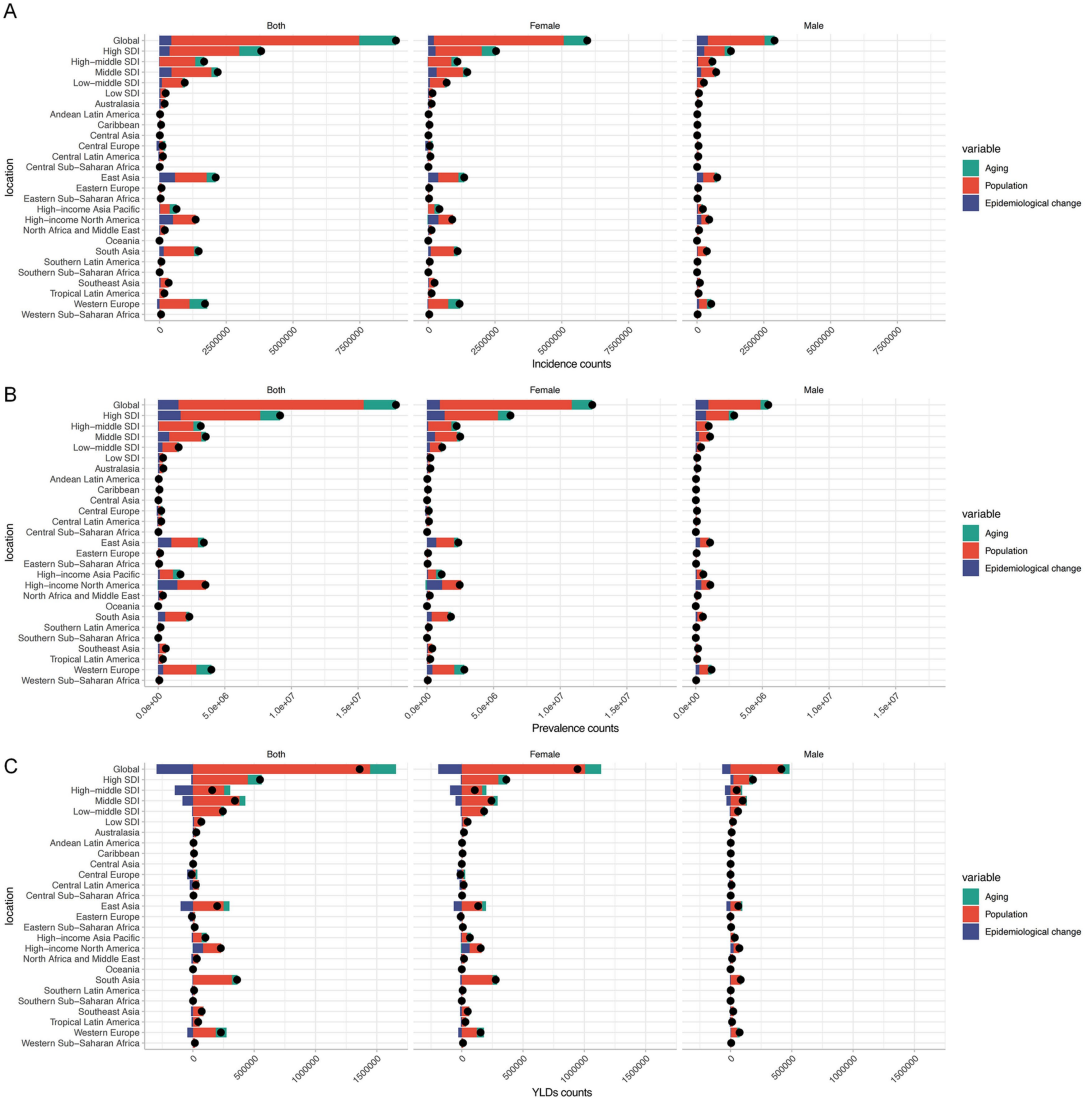
Decomposition analysis for HFs (≥55 years) burden. Decomposition analysis results of new cases **(A)**, prevalence count **(B)**, and YLDs **(C)** for HFs (≥55 years), by global, 5 SDI regions, and 21 GBD regions.

Decomposition analysis showed that the rising burden in HFs (≥55 years) was primarily driven by population growth, followed by aging, with minimal or negative contributions from epidemiological changes. These patterns were consistent across sexes and regions. Globally, population growth, aging, and epidemiological changes accounted for 79.35, 15.58, and 5.07% of the increase in new cases, and 77.95, 13.53, and 8.52% of the rise in prevalence. In contrast, YLDs rose by 106.22 and 15.54% due to population growth and aging, but declined by 21.76% due to epidemiological changes. Other regional trends aligned with global patterns (Figures 8A–C; Supplementary Table S6).

### 3.9 Predictive analysis results for HFs (≥55 years)

**Figure 9:**
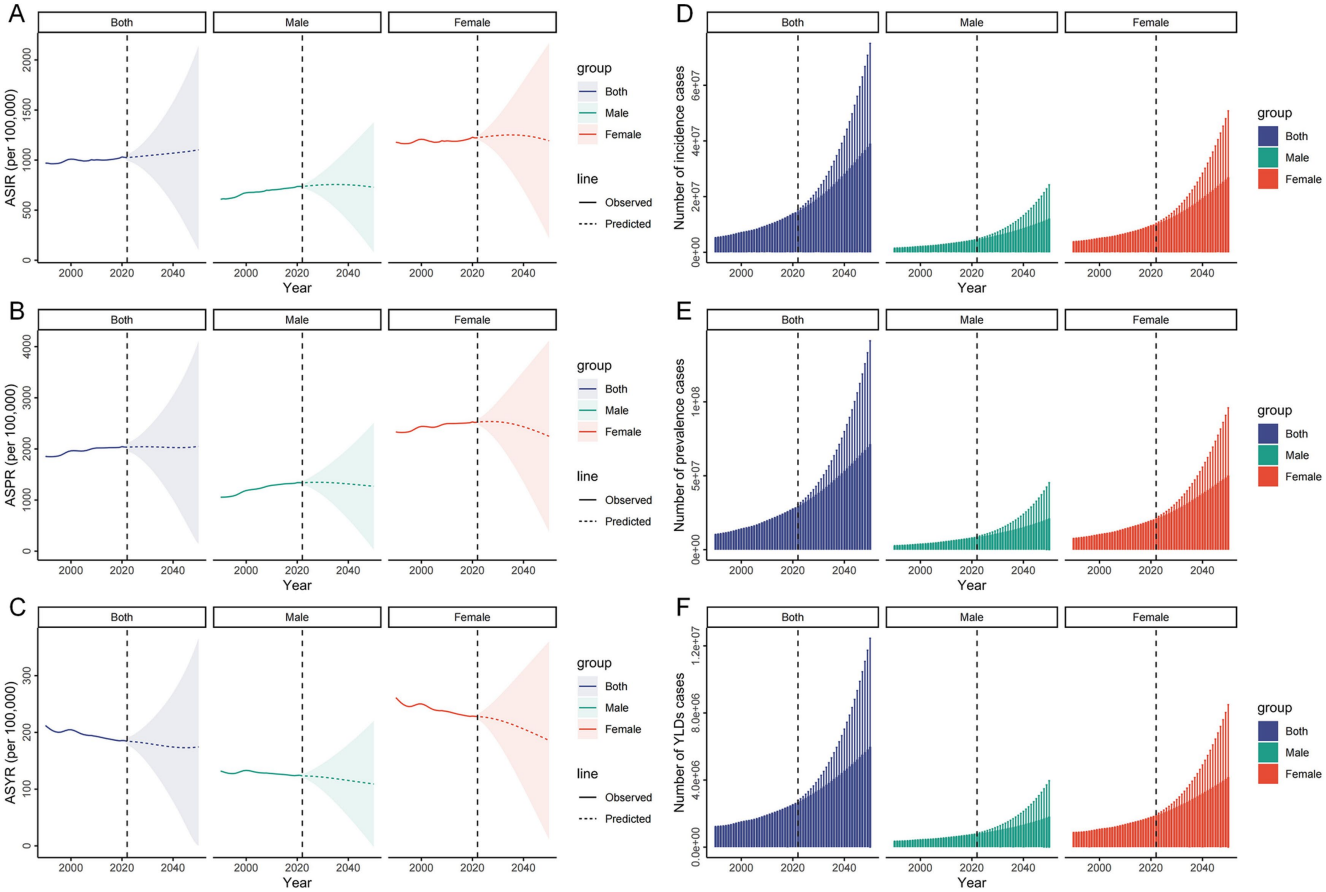
Predictive analysis for HFs (≥55 years) burden. Predictive analysis results of ASIR **(A)**, ASPR **(B)**, and ASYR **(C)** for HFs (≥55 years) globally, by sex. Predictive analysis results of new cases **(D)**, prevalence count **(E)**, and YLDs **(F)** for HFs (≥55 years) globally, by sex.

By 2050, the global ASIR and ASPR of HFs (≥55 years) are projected to reach 1,102.66 (95% CI: 101.40–2,142.83) and 2,052.14 (95% CI: 141.30–4,112.55) per 100,000, respectively. ASYR is expected to decline slightly to 174.43 (95% CI: 0–365.91) per 100,000 person-years (Figures 9A–C). Besides, the absolute burden will increase markedly, with new cases estimated at 26,393,436 (95% CI: 4,174,866–48,612,008), prevalent cases at 48,448,804 (95% CI: 5,870,231–91,027,360), and YLDs at 4,056,285 (95% CI: 140,451–7,972,119) years (Figures 9D–F). While ASRs for both sexes are expected to decline slightly, their absolute burdens are projected to continue rising over the next three decades (Supplementary Table S7).

### 3.10 Health inequality analysis results for HFs (≥55 years)

**Figure 10:**
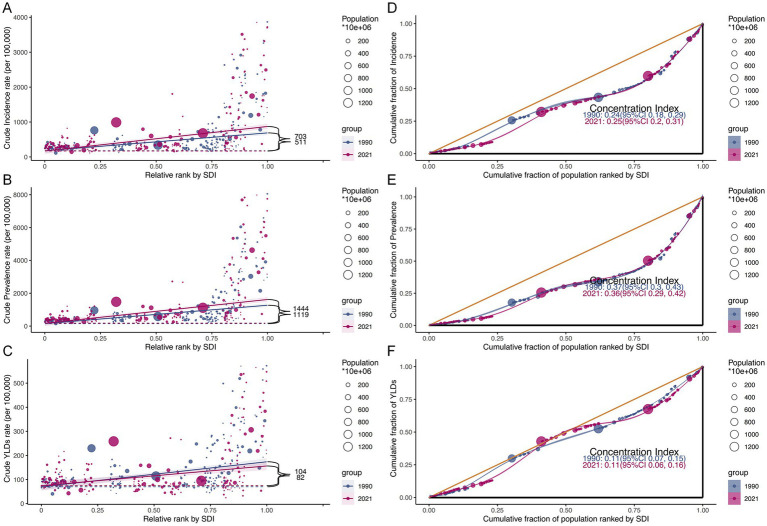
Health inequality analysis for HFs (≥55 years) burden. Health inequality analysis of crude incidence rate **(A)**, crude prevalence rate **(B)**, and crude YLDs rate **(C)** by SDI rank for 1990 and 2021, with the black label representing the slope index of inequality. Cumulative fraction of population by SDI rank with concentration index for incidence **(D)**, prevalence **(E)**, and YLDs **(F)** in 1990 and 2021.

Inequity analysis revealed that health inequity was disproportionately concentrated in High SDI region. In 1990, the slope index for crude incidence, prevalence, and YLDs rates in highest SDI region compared to lowest SDI region were 511, 1,119, and 104, respectively. By 2021, the slope index for crude incidence and prevalence rates had increased to 703 and 1,444, while the slope index for the YLDs rate had declined to 82 (Figures 10A–C). Concentration index analysis indicated that the distributional inequality of incidence, prevalence, and YLDs remained relatively stable over time, with all concentration indices consistently above zero (Figures 10D–F).

### 3.11 Frontier analysis results for HFs (≥55 years)

**Figure 11:**
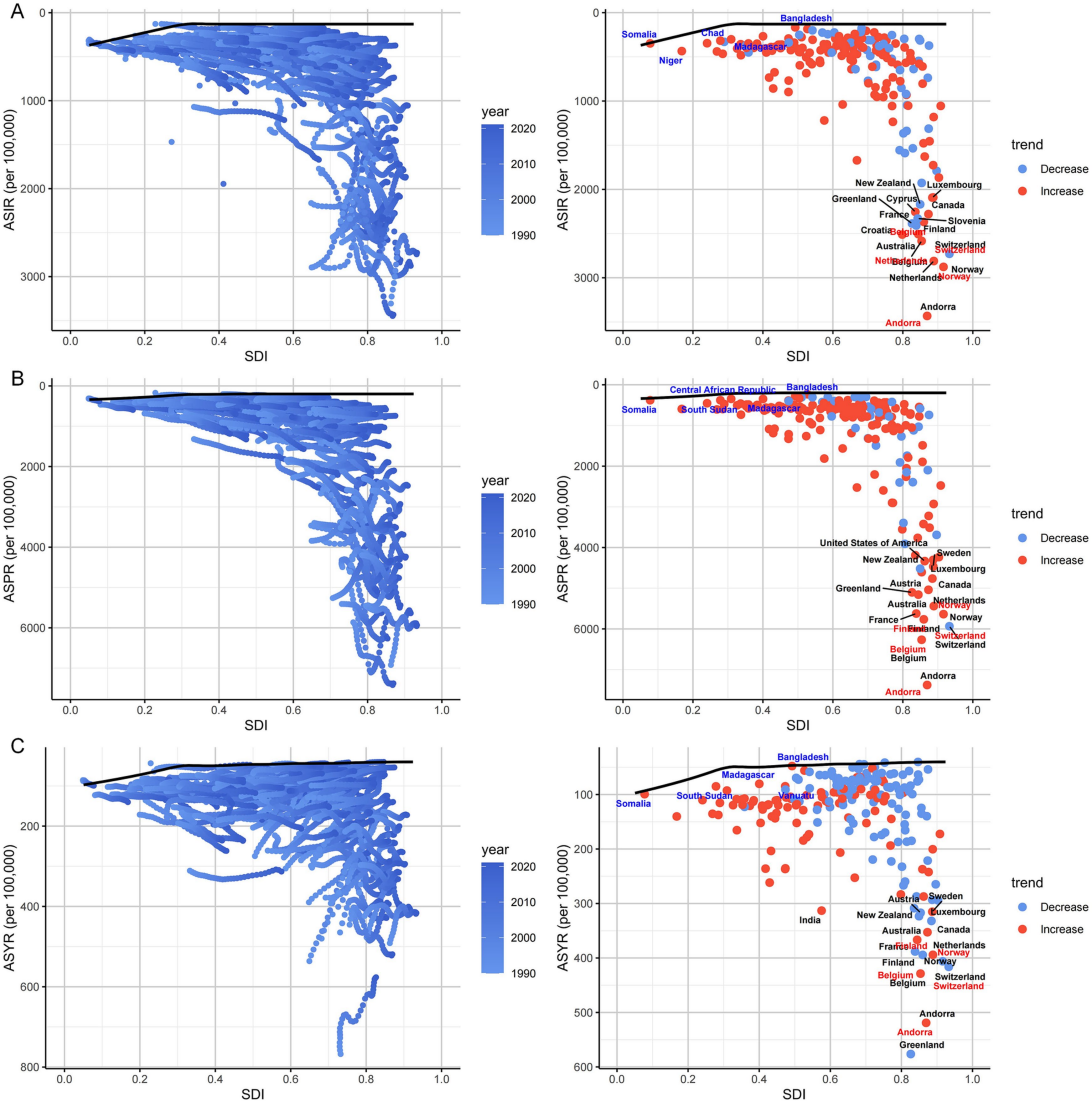
Frontier analysis for HFs (≥55 years) burden. Frontier analysis of ASIR **(A)**, ASPR **(B)**, and ASYR **(C)** for HFs (≥55 years) from 1990 to 2021. Boundaries are delineated by solid black lines, with countries and territories represented by dots. The top 15 countries with the largest effective difference from the frontier are marked in black. Lower SDI (<0.5) countries with the smallest effective difference from the frontier are highlighted in blue, while higher SDI (>0.85) countries with the largest effective difference are highlighted in red. Red dots indicate an increase in the ASR for HFs (≥55 years) from 1990 to 2021; blue dots indicate a decrease in the ASR for HFs (≥55 years) during the same period.

Figure 11 further illustrated that from 1990 to 2021, the global ASIR and ASPR of HFs (≥55 years) increased with rising SDI. In contrast, ASYR showed divergent patterns across SDI levels—rising in low SDI countries (SDI < 0.50) and generally declining in middle-to high-SDI countries. The 15 countries with the greatest potential to reduce disease burden were predominantly located in higher SDI countries. For example, regarding ASYR, countries like Bangladesh (SDI < 0.50) showed minimal gaps from the optimal frontier, while higher SDI countries such as Switzerland exhibited the largest gaps, indicating substantial room for reducing disability burden (Figures 11A–C). Detailed data on frontier differences across 204 countries and territories over the past 32 years are presented in Supplementary Tables S8a–c.

### 3.12 Etiology analysis results for HFs (≥55 years)

**Figure 12:**
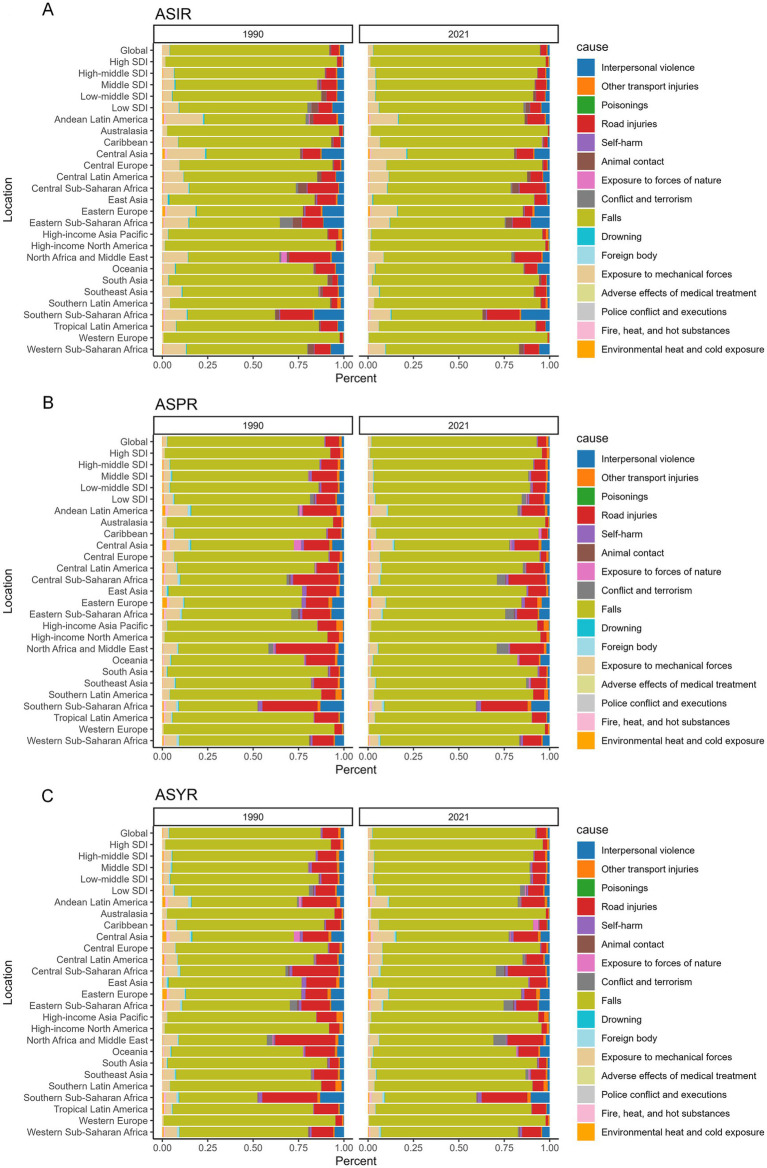
Etiology analysis for HFs (≥55 years) burden. Percentage of risk factors influencing ASIR **(A)**, ASPR **(B)**, and ASYR **(C)** for HFs (≥55 years) in 1990 and 2021, by global, 5 SDI regions, and 21 GBD regions.

In 2021, falls remained the leading cause of HFs (≥55 years) globally, accounting for 91.55% of ASIR, 90.77% of ASPR, and 89.47% of ASYR. This proportion was even more pronounced in regions with higher SDI, such as Western Europe and High-income North America. Compared to 1990, the burden attributable to falls further increased by 2021. Detailed data are provided in Figure 12 and Supplementary Table S9.

## 4 Discussion

Based on GBD 2021 data, we found that although ASYR declined in certain regions, ASIR, ASPR, and the absolute burden among HFs (≥55 years) continued to increase globally and are projected to rise further over the next 30 years. This trend reflects the complexity of global health inequities shaped by the combined effects of age, sex, region, and social development levels.

### 4.1 Trends in the burden of HFs (≥55 years) from 1990 to 2021

HFs are among the most disabling and fatal fractures in older adults and have become a major global public health concern (9). Our study shows that the global ASIR, ASPR, and absolute burden of HFs (≥55 years) have continued to rise over the past three decades. According to the BAPC model, this trend is expected to persist in the coming decades. By 2050, the number of new cases is projected to exceed 26 million, while global prevalence may surpass 48 million. Despite the increase in total global cases, the ASYR showed a declining trend. This may reflect advancements in osteoporosis management, broader use of anti-osteoporotic medications, and improvements in postoperative rehabilitation, all of which have contributed to a partial reduction in the disability burden (21). Shen et al. reported that in most middle-to high-income countries, a stable decline in ASYR was achieved through multifaceted interventions, demonstrating the synergistic effects of comprehensive prevention strategies (22). Further decomposition analysis revealed that the recent increase in the burden was primarily driven by population growth (approximately 79%) and aging (around 15%), with the most pronounced increases observed in Asia and Latin America (23, 24). These regions not only have large and rapidly expanding aging populations but also face significant deficiencies in fracture prevention, fall intervention, and rehabilitation systems. As a result, they may become high-risk areas for future increases in global burden, while simultaneously representing critical windows of opportunity for implementing forward-looking intervention strategies (8, 25). Therefore, the current upward trend not only reflects the impact of global demographic shifts in both population size and age structure on HFs (≥55 years) burden, but also underscores the urgent need for prevention-oriented and earlier-stage intervention strategies.

### 4.2 Socio-demographic development and regional disparities

The burden of HFs (≥55 years) exhibits significant socio-demographic development and regional disparities. Overall, ASIR, ASPR, and ASYR are positively correlated with SDI. In lower SDI regions (SDI < 0.6), the burden remains relatively low, possibly due to limited population aging and gaps in epidemiological data availability (15, 26). However, as socio-demographic development advanced, particularly in regions where SDI exceeded 0.75, the disease burden increases markedly. This reflects the combined influence of a growing aging population and an increasing prevalence of osteoporosis (27). Although High SDI region has historically borne the greatest global burden of disease and experienced marked health inequities, the rate of increase has moderated in recent years, with ASYR showing a downward trend. This suggests sustained advancements in healthcare infrastructure, particularly in areas such as trauma care, postoperative rehabilitation, and integrated osteoporosis management, which have collectively contributed to improved disability outcomes (28, 29). For example, 72% of HFs patients in Germany undergo surgery within 24 h of hospitalization, and 52.8% of patients in the U.S. are seamlessly transferred to rehabilitation institutions for functional recovery (30, 31). Additionally, high-income countries such as those in Northern Europe and Japan have widely implemented population-based screening programs, multidisciplinary preoperative assessment systems, and standardized postoperative rehabilitation pathways, all of which have contributed to improved patient survival and quality of life (32–34). Therefore, High SDI region should continue to focus on secondary prevention through early screening and timely intervention. Integrating osteoporosis management, postoperative rehabilitation, and long-term care at the community level are essential to address the growing fracture burden and the risk of re-fractures associated with aging. In contrast, Low SDI region currently bears a lower overall burden, but the rate of increase is accelerating. In Central Sub-Saharan African countries such as the Democratic Republic of the Congo, weak public health infrastructure, limited medical resources, and frequent social unrest have led to extremely low rates of diagnosis, surgical treatment, and rehabilitation for HFs. As a result, the disease burden continues to rise (35, 36). This highlights the urgent need to strengthen policy interventions and healthcare infrastructure. It is also noteworthy that even within similar SDI levels, management outcomes for HFs (≥55 years) vary across countries and regions. As an illustration, in the High SDI region of Australasia, the disease burden remains high and continues to rise. This is probably associated with significant population aging, advanced diagnostic technologies, and robust health insurance and welfare systems (15).

### 4.3 Age and sex differences

Age and sex are key demographic drivers of HFs (≥55 years) burden (37). This study shows that individuals aged ≥70 have markedly higher ASIR, ASPR, and ASYR than those aged 55–69, and that women bear a substantially greater overall burden. Although this sex difference is well established, the underlying dynamic trends still warrant further exploration. Specifically, we observed a steeper rise in ASIR and ASPR among men, accompanied by a slower decline in ASYR compared to women. This “sex asymmetry” in the rate of improvement suggests that there are significant gaps in the identification and management of osteoporosis prevention and treatment systems for men. Previous studies have reported that approximately 12% of men over the age of 50 will experience at least one osteoporotic fracture in their lifetime (38). However, the identification and treatment coverage of osteoporosis in men remain substantially lower than in women. While women typically enter primary prevention programs after menopause, most men are not included in intervention pathways until after their first fracture. In addition, men are less likely to receive anti-osteoporotic therapy or participate in postoperative rehabilitation, resulting in higher short-term mortality and poorer functional recovery following HFs (39, 40). In contrast, although older women are at higher risk, established mechanisms for screening, pharmacologic treatment, and lifestyle interventions are more widely available (9). Therefore, public health efforts should not only maintain their focus on older women but also routinely implement osteoporosis screening and fall prevention education for men aged ≥65. Clinical practice also should prioritize enhancing adherence to postoperative rehabilitation and expanding medication coverage to address sex disparities in osteoporosis management.

### 4.4 Major causes and prevention challenges

Falls represents the leading cause of accidental injury and premature mortality in older adults, accounting for approximately 80% of fall-related disabilities among individuals over the age of 50 (41). Our study confirms that falls remains the leading cause of HFs (≥55 years), accounting for nearly 90% of cases among the 16 injury causes in the GBD framework. This pattern is particularly evident in high-income countries and underscores the growing health challenges associated with population aging (42). The causes of falls are multifactorial, including physiological degeneration, chronic comorbidities, polypharmacy, and socio-economic conditions. However, the core issue is the continued failure to effectively identify and intervene on high-risk factors (43). Currently, insufficient osteoporosis screening, overlooked synergistic effects of polypharmacy and chronic disease, and fragmented older adult rehabilitation services hinder prevention efforts (7). Even in high-income countries, problems such as inconsistent rehabilitation protocols and delayed interventions remain prevalent (9). Therefore, effective prevention strategies should extend beyond traditional medical approaches and adopt a comprehensive model focused on functional maintenance, medication management, environmental optimization, and community support. In regions with large older populations, priority should be given to establishing integrated, cross-sectoral fall prevention systems that ensure closed-loop management from risk identification to intervention (3, 44).

### 4.5 Limitations

Our study estimates the burden of HFs (≥55 years) using GBD 2021 data; therefore, its limitations are inherently aligned with those of the GBD study itself. While the GBD model plays a critical role in monitoring and comparing disease burden globally, its estimation process is subject to several methodological limitations, particularly in lower SDI regions. First, due to the lack of high-quality epidemiological data in low-income and underreported areas, the GBD model often extrapolates from neighboring regions or global averages. This can lead to a systematic underestimation of the actual disease burden (45). Second, the modeling of multimorbidity in GBD remains relatively simplified and fails to account for complex disease interactions in older populations, limiting its accuracy in reflecting overall health burden (46, 47). Although the GBD reports provide UIs, the transparency regarding model assumptions, sensitivity analyses, and sources of error remains inadequate (45). Moreover, the use of a uniform assumption for disease natural history does not sufficiently reflect cross-country differences in healthcare systems, diagnostic criteria, and cultural contexts, thereby reducing the model’s capacity to capture regional heterogeneity (45, 48).

## 5 Conclusion

In summary, the global burden of HFs (≥55 years) continues to rise, largely driven by population growth and aging. Although ASYR has declined in some countries, the absolute burden remains high as case numbers increase. Significant disparities exist across socio-demographic levels. High SDI region shows slower growth due to more mature intervention systems, yet still bears a substantial burden given its large aging population. Low SDI region, facing rapid aging and limited healthcare capacity, is experiencing a sharper rise. Sex differences remain evident, with men showing lower rates of screening, treatment, and rehabilitation. Falls continues to be the leading cause of HFs (≥55 years). Thus, High SDI region should enhance system integration and long-term care, while Low SDI region needs to expand screening and early intervention, strengthen primary healthcare systems, and improve access to rehabilitation to contain the future burden.

## Data Availability

Publicly available datasets were analyzed in this study. This data can be found here: http://ghdx.healthdata.org/gbd-results-tool.
