# Supplementary material for: Geotemporal disparities in hip fractures burden among individuals aged ≥55 years (1990–2021) with projections to 2050
Source: Front Public Health. 2025 Sep 1;13:1600452. doi: 10.3389/fpubh.2025.1600452 (PMC12433851; doi:10.3389/fpubh.2025.1600452)
Supplement: Supplementary file 1 [file Supplementary_file_1.docx]

***Supplementary Material***

**1.1 Supplementary Figures**


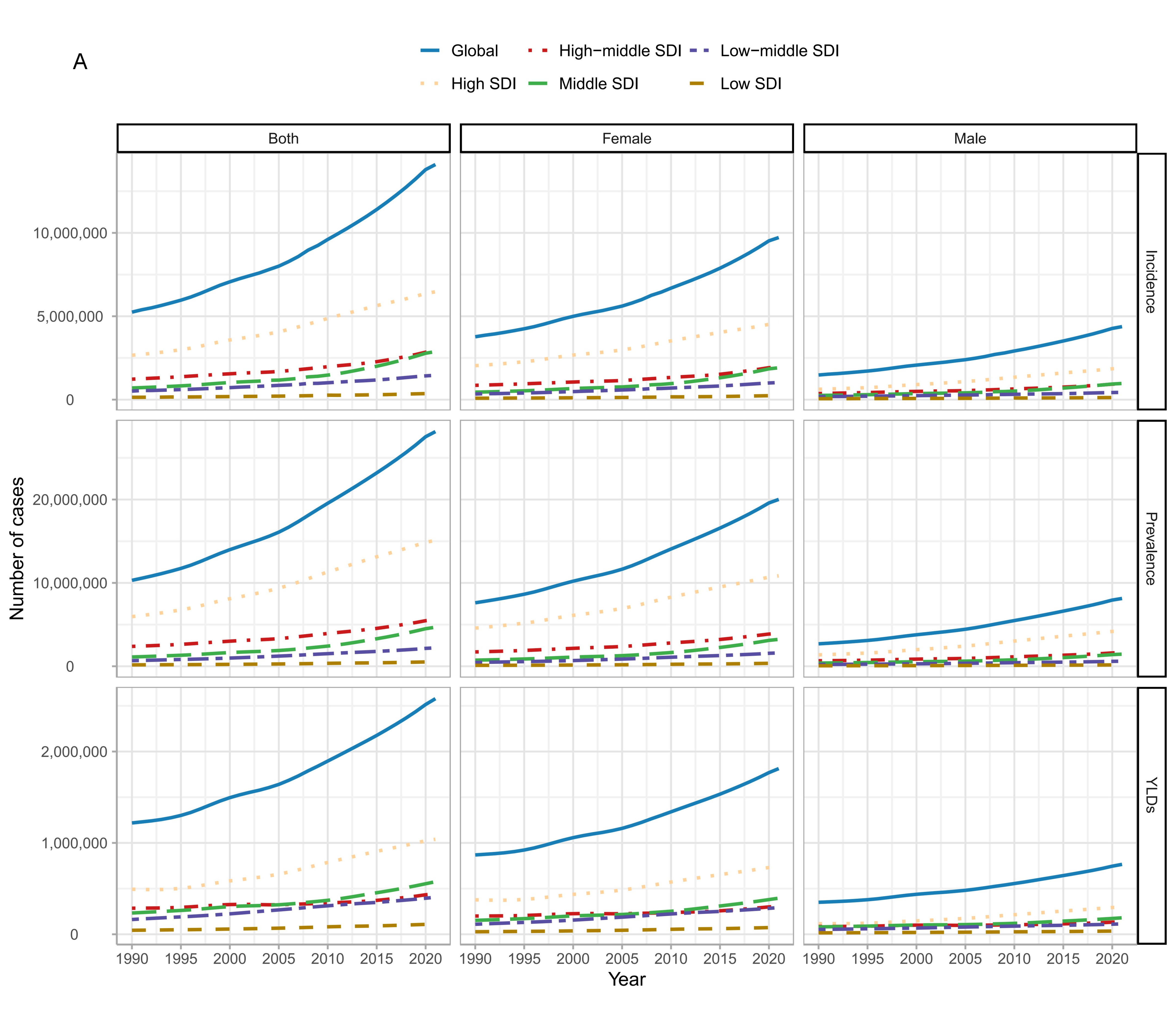


**Figure S1.** Global and five SDI regional absolute burden of HFs (≥55 years). **(A)** New cases, prevalence count, and YLDs for HFs (≥55 years) from 1990 to 2021, by sex and SDI.


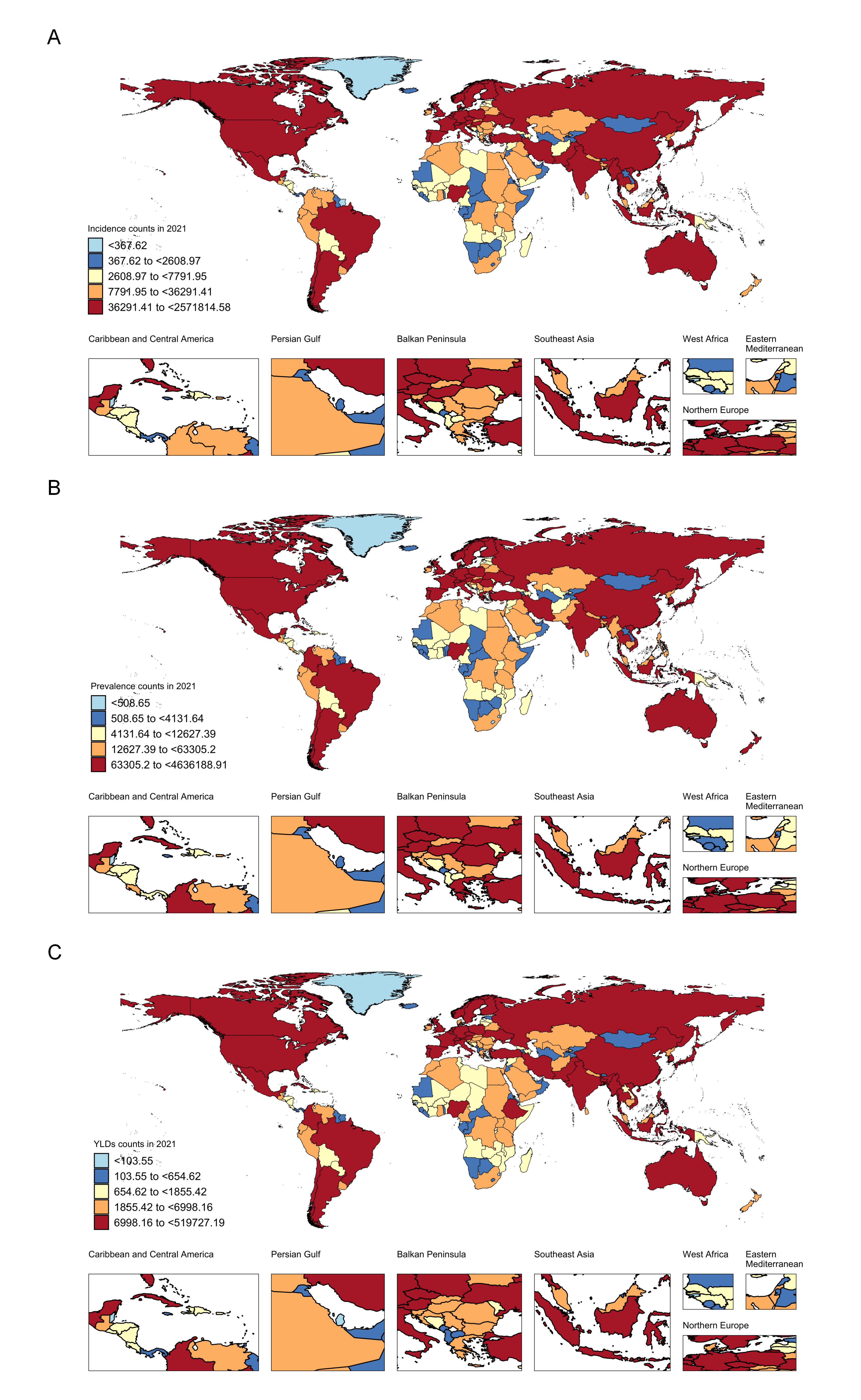


**Figure S2.** National and regional absolute burden of HFs (≥55 years). New cases **(A)**, prevalence count **(B)**, and YLDs **(C)** for HFs (≥55 years) in 204 countries and territories in 2021.


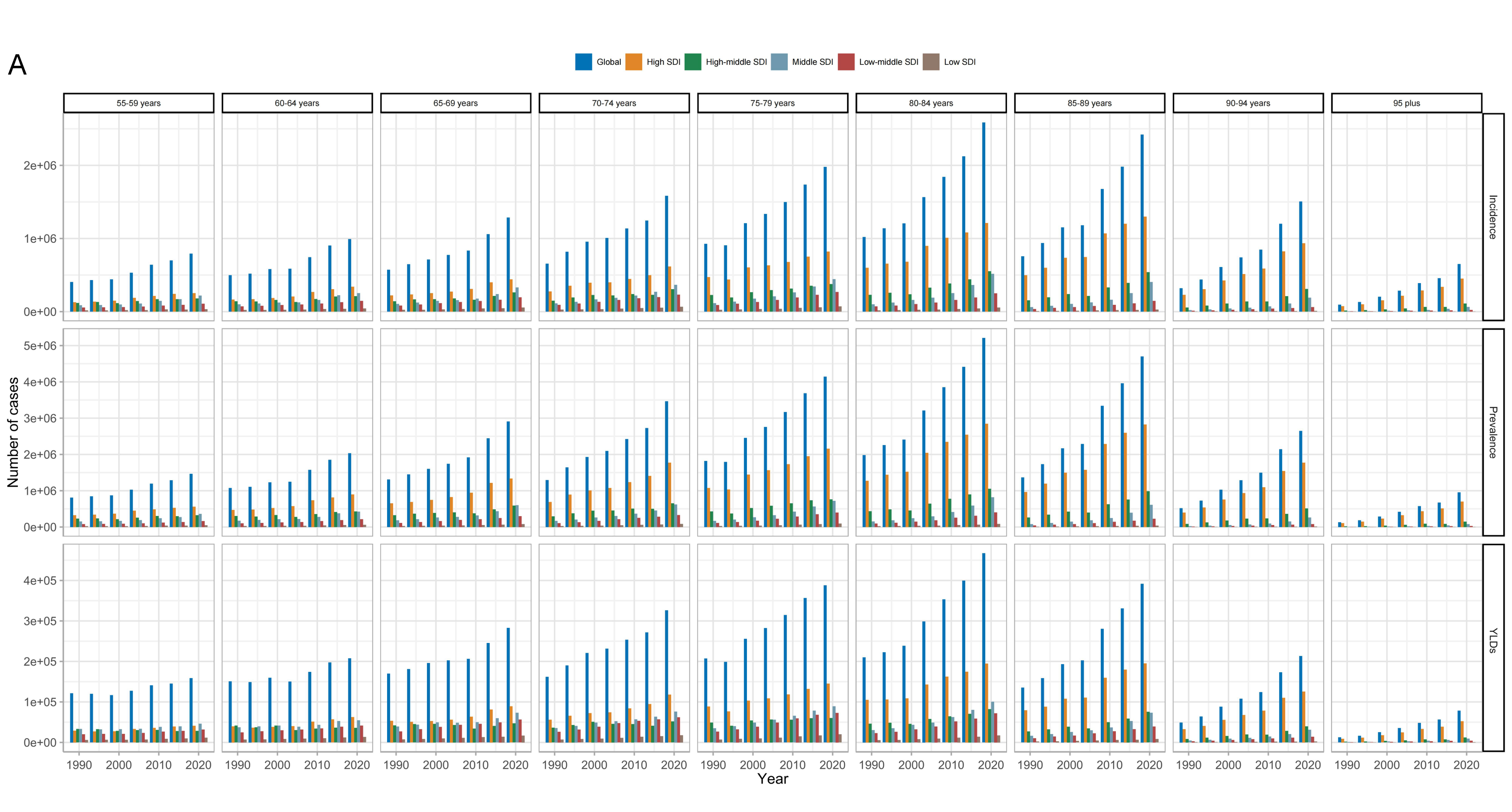


**Figure S3.** Age-time trends in HFs (≥55 years) burden. **(A)** Age-time trends in new cases, prevalence count, and YLDs for HFs (≥55 years) from 1990 to 2021, by 5 SDI regions.


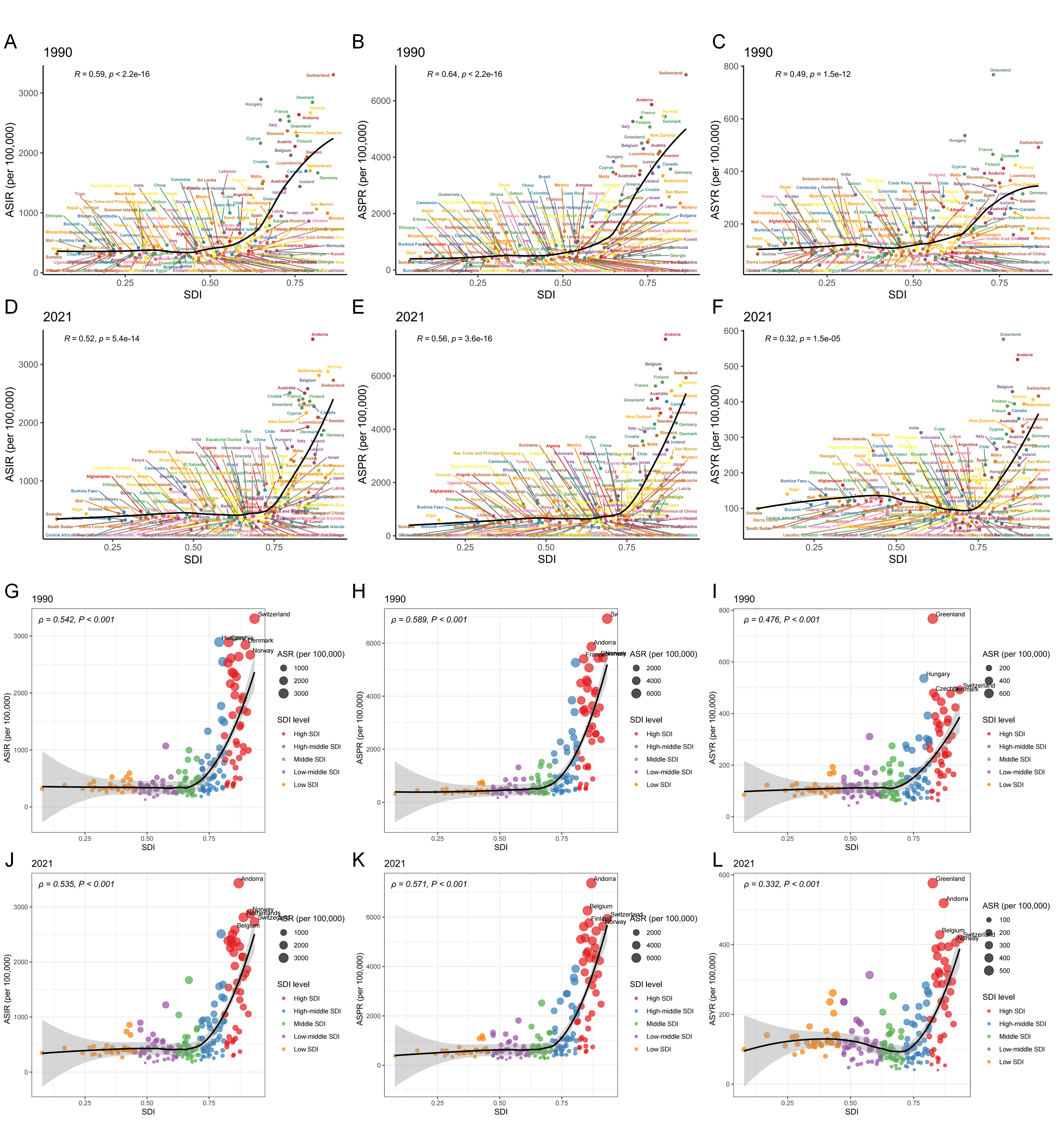


**Figure S4.** The relationship between HFs (≥55 years) burden and SDI. Relationship between ASIR **(A)**, ASPR **(B)**, ASYR **(C)** for HFs (≥55 years) and SDI in 204 countries and territories in 1990. Relationship between ASIR **(D)**, ASPR **(E)**, ASYR **(F)** for HFs (≥55 years) and SDI in 204 countries and territories in 2021. Relationship between ASIR **(G)**, ASPR **(H)**, ASYR **(I)** for HFs (≥55 years) and SDI in 1990, by 5 SDI regions. The scatter points represent 204 countries and territories, with black labels indicating the top 5 countries for ASIR, ASPR, and ASYR. Relationship between ASIR **(J)**, ASPR **(K)**, ASYR **(L)** for HFs (≥55 years) and SDI in 2021, by 5 SDI regions. The scatter points represent 204 countries and territories, with black labels indicating the top 5 countries for ASIR, ASPR, and ASYR.

**2.1 Supplementary Tables**

**Table S1. The results of ASIR, ASPR, and ASYR for HFs (≥55 years) across global, 5 SDI regions, and 21 GBD regions**

| **measure** | **location** | **sex** | **year** | **value** | **95% UI_upper** | **95% UI_lower** |
| --- | --- | --- | --- | --- | --- | --- |
| ASIR | Global | Male | 1990 | 608.73 | 836.38 | 434.54 |
| ASIR | Global | Male | 2021 | 737.80 | 1012.82 | 524.97 |
| ASIR | Global | Female | 1990 | 1177.82 | 1651.99 | 812.24 |
| ASIR | Global | Female | 2021 | 1221.12 | 1694.85 | 846.61 |
| ASIR | Global | Both | 1990 | 969.80 | 1347.95 | 676.22 |
| ASIR | Global | Both | 2021 | 1027.46 | 1416.07 | 719.73 |
| ASIR | East Asia | Male | 1990 | 355.63 | 516.29 | 236.87 |
| ASIR | East Asia | Male | 2021 | 657.80 | 944.72 | 443.97 |
| ASIR | East Asia | Female | 1990 | 544.37 | 815.61 | 352.93 |
| ASIR | East Asia | Female | 2021 | 917.92 | 1344.41 | 601.86 |
| ASIR | East Asia | Both | 1990 | 472.91 | 702.05 | 310.10 |
| ASIR | East Asia | Both | 2021 | 821.19 | 1194.91 | 543.64 |
| ASIR | Southeast Asia | Male | 1990 | 302.06 | 408.87 | 217.94 |
| ASIR | Southeast Asia | Male | 2021 | 367.18 | 499.16 | 264.96 |
| ASIR | Southeast Asia | Female | 1990 | 609.76 | 868.86 | 414.45 |
| ASIR | Southeast Asia | Female | 2021 | 703.10 | 985.15 | 485.43 |
| ASIR | Southeast Asia | Both | 1990 | 483.21 | 677.12 | 335.69 |
| ASIR | Southeast Asia | Both | 2021 | 572.34 | 793.28 | 401.40 |
| ASIR | Oceania | Male | 1990 | 165.03 | 214.12 | 124.80 |
| ASIR | Oceania | Male | 2021 | 178.81 | 231.01 | 135.35 |
| ASIR | Oceania | Female | 1990 | 677.83 | 983.44 | 451.78 |
| ASIR | Oceania | Female | 2021 | 1021.15 | 1452.91 | 687.70 |
| ASIR | Oceania | Both | 1990 | 436.95 | 615.80 | 302.53 |
| ASIR | Oceania | Both | 2021 | 611.35 | 852.68 | 424.97 |
| ASIR | Central Asia | Male | 1990 | 276.55 | 378.55 | 203.22 |
| ASIR | Central Asia | Male | 2021 | 290.26 | 390.27 | 215.15 |
| ASIR | Central Asia | Female | 1990 | 235.51 | 321.66 | 171.38 |
| ASIR | Central Asia | Female | 2021 | 273.95 | 370.41 | 199.37 |
| ASIR | Central Asia | Both | 1990 | 253.45 | 342.95 | 187.05 |
| ASIR | Central Asia | Both | 2021 | 284.48 | 380.02 | 210.58 |
| ASIR | Central Europe | Male | 1990 | 948.32 | 1295.05 | 685.26 |
| ASIR | Central Europe | Male | 2021 | 954.95 | 1322.49 | 678.35 |
| ASIR | Central Europe | Female | 1990 | 1631.31 | 2260.05 | 1148.23 |
| ASIR | Central Europe | Female | 2021 | 1113.26 | 1536.73 | 776.15 |
| ASIR | Central Europe | Both | 1990 | 1410.77 | 1937.43 | 1003.73 |
| ASIR | Central Europe | Both | 2021 | 1084.20 | 1500.47 | 764.80 |
| ASIR | Eastern Europe | Male | 1990 | 580.07 | 800.72 | 413.55 |
| ASIR | Eastern Europe | Male | 2021 | 601.12 | 828.74 | 424.28 |
| ASIR | Eastern Europe | Female | 1990 | 494.74 | 695.49 | 346.57 |
| ASIR | Eastern Europe | Female | 2021 | 471.38 | 656.94 | 331.39 |
| ASIR | Eastern Europe | Both | 1990 | 536.31 | 747.88 | 379.14 |
| ASIR | Eastern Europe | Both | 2021 | 532.66 | 736.32 | 375.76 |
| ASIR | High-income Asia Pacific | Male | 1990 | 702.58 | 1025.37 | 467.80 |
| ASIR | High-income Asia Pacific | Male | 2021 | 815.63 | 1147.87 | 558.76 |
| ASIR | High-income Asia Pacific | Female | 1990 | 950.16 | 1389.88 | 622.35 |
| ASIR | High-income Asia Pacific | Female | 2021 | 931.10 | 1304.84 | 638.72 |
| ASIR | High-income Asia Pacific | Both | 1990 | 875.66 | 1277.95 | 576.25 |
| ASIR | High-income Asia Pacific | Both | 2021 | 897.06 | 1255.34 | 619.07 |
| ASIR | Western Europe | Male | 1990 | 998.91 | 1349.40 | 713.27 |
| ASIR | Western Europe | Male | 2021 | 1143.87 | 1531.47 | 827.58 |
| ASIR | Western Europe | Female | 1990 | 2254.47 | 3084.81 | 1571.56 |
| ASIR | Western Europe | Female | 2021 | 2218.42 | 2971.95 | 1578.90 |
| ASIR | Western Europe | Both | 1990 | 1837.67 | 2495.03 | 1293.66 |
| ASIR | Western Europe | Both | 2021 | 1784.06 | 2383.49 | 1283.36 |
| ASIR | Australasia | Male | 1990 | 1106.75 | 1474.02 | 797.01 |
| ASIR | Australasia | Male | 2021 | 1850.34 | 2456.93 | 1327.01 |
| ASIR | Australasia | Female | 1990 | 2075.03 | 2829.57 | 1455.60 |
| ASIR | Australasia | Female | 2021 | 2913.15 | 3911.11 | 2063.35 |
| ASIR | Australasia | Both | 1990 | 1734.38 | 2331.79 | 1228.88 |
| ASIR | Australasia | Both | 2021 | 2455.40 | 3258.05 | 1765.77 |
| ASIR | Southern Latin America | Male | 1990 | 454.03 | 593.84 | 335.78 |
| ASIR | Southern Latin America | Male | 2021 | 489.32 | 641.33 | 360.35 |
| ASIR | Southern Latin America | Female | 1990 | 854.99 | 1156.57 | 611.06 |
| ASIR | Southern Latin America | Female | 2021 | 983.91 | 1317.39 | 706.68 |
| ASIR | Southern Latin America | Both | 1990 | 704.72 | 941.18 | 511.59 |
| ASIR | Southern Latin America | Both | 2021 | 801.41 | 1057.75 | 586.09 |
| ASIR | High-income North America | Male | 1990 | 832.45 | 1216.77 | 552.82 |
| ASIR | High-income North America | Male | 2021 | 1267.68 | 1801.66 | 852.02 |
| ASIR | High-income North America | Female | 1990 | 1324.26 | 1965.21 | 852.61 |
| ASIR | High-income North America | Female | 2021 | 2039.46 | 2935.94 | 1338.73 |
| ASIR | High-income North America | Both | 1990 | 1144.80 | 1687.60 | 744.76 |
| ASIR | High-income North America | Both | 2021 | 1705.11 | 2439.09 | 1127.41 |
| ASIR | Caribbean | Male | 1990 | 407.15 | 545.66 | 301.62 |
| ASIR | Caribbean | Male | 2021 | 566.54 | 761.05 | 411.08 |
| ASIR | Caribbean | Female | 1990 | 799.19 | 1099.95 | 560.70 |
| ASIR | Caribbean | Female | 2021 | 1187.01 | 1604.18 | 838.13 |
| ASIR | Caribbean | Both | 1990 | 620.09 | 840.25 | 445.03 |
| ASIR | Caribbean | Both | 2021 | 922.94 | 1241.66 | 663.07 |
| ASIR | Andean Latin America | Male | 1990 | 301.51 | 406.33 | 222.80 |
| ASIR | Andean Latin America | Male | 2021 | 341.21 | 455.69 | 253.64 |
| ASIR | Andean Latin America | Female | 1990 | 304.64 | 407.28 | 224.35 |
| ASIR | Andean Latin America | Female | 2021 | 382.43 | 518.95 | 278.60 |
| ASIR | Andean Latin America | Both | 1990 | 305.03 | 407.61 | 226.12 |
| ASIR | Andean Latin America | Both | 2021 | 366.04 | 490.27 | 270.52 |
| ASIR | Central Latin America | Male | 1990 | 573.43 | 784.87 | 413.26 |
| ASIR | Central Latin America | Male | 2021 | 455.52 | 614.72 | 332.07 |
| ASIR | Central Latin America | Female | 1990 | 760.16 | 1081.48 | 522.51 |
| ASIR | Central Latin America | Female | 2021 | 582.45 | 809.89 | 408.08 |
| ASIR | Central Latin America | Both | 1990 | 672.78 | 936.52 | 474.38 |
| ASIR | Central Latin America | Both | 2021 | 530.32 | 727.43 | 379.00 |
| ASIR | Tropical Latin America | Male | 1990 | 516.18 | 725.41 | 357.03 |
| ASIR | Tropical Latin America | Male | 2021 | 511.43 | 715.62 | 353.07 |
| ASIR | Tropical Latin America | Female | 1990 | 671.74 | 989.98 | 440.92 |
| ASIR | Tropical Latin America | Female | 2021 | 712.81 | 1036.60 | 472.42 |
| ASIR | Tropical Latin America | Both | 1990 | 610.28 | 880.91 | 409.86 |
| ASIR | Tropical Latin America | Both | 2021 | 635.82 | 910.01 | 429.76 |
| ASIR | North Africa and Middle East | Male | 1990 | 319.40 | 419.45 | 235.96 |
| ASIR | North Africa and Middle East | Male | 2021 | 362.40 | 473.65 | 269.91 |
| ASIR | North Africa and Middle East | Female | 1990 | 408.48 | 547.88 | 295.77 |
| ASIR | North Africa and Middle East | Female | 2021 | 595.56 | 808.54 | 423.65 |
| ASIR | North Africa and Middle East | Both | 1990 | 369.15 | 488.72 | 272.38 |
| ASIR | North Africa and Middle East | Both | 2021 | 481.69 | 643.75 | 351.06 |
| ASIR | South Asia | Male | 1990 | 503.73 | 733.34 | 335.23 |
| ASIR | South Asia | Male | 2021 | 567.94 | 822.91 | 381.36 |
| ASIR | South Asia | Female | 1990 | 1250.36 | 1927.96 | 773.12 |
| ASIR | South Asia | Female | 2021 | 1436.22 | 2140.48 | 916.20 |
| ASIR | South Asia | Both | 1990 | 868.35 | 1317.16 | 552.25 |
| ASIR | South Asia | Both | 2021 | 1037.94 | 1530.88 | 672.47 |
| ASIR | Central Sub-Saharan Africa | Male | 1990 | 263.07 | 347.88 | 194.54 |
| ASIR | Central Sub-Saharan Africa | Male | 2021 | 278.03 | 367.44 | 207.54 |
| ASIR | Central Sub-Saharan Africa | Female | 1990 | 342.71 | 465.09 | 245.96 |
| ASIR | Central Sub-Saharan Africa | Female | 2021 | 397.80 | 539.57 | 285.24 |
| ASIR | Central Sub-Saharan Africa | Both | 1990 | 305.38 | 409.12 | 222.47 |
| ASIR | Central Sub-Saharan Africa | Both | 2021 | 354.82 | 474.55 | 257.77 |
| ASIR | Eastern Sub-Saharan Africa | Male | 1990 | 331.27 | 443.03 | 245.90 |
| ASIR | Eastern Sub-Saharan Africa | Male | 2021 | 334.65 | 442.51 | 249.58 |
| ASIR | Eastern Sub-Saharan Africa | Female | 1990 | 428.39 | 587.23 | 309.35 |
| ASIR | Eastern Sub-Saharan Africa | Female | 2021 | 469.60 | 637.96 | 338.57 |
| ASIR | Eastern Sub-Saharan Africa | Both | 1990 | 384.18 | 518.70 | 282.99 |
| ASIR | Eastern Sub-Saharan Africa | Both | 2021 | 412.98 | 554.87 | 303.45 |
| ASIR | Southern Sub-Saharan Africa | Male | 1990 | 249.32 | 334.93 | 181.16 |
| ASIR | Southern Sub-Saharan Africa | Male | 2021 | 231.48 | 308.98 | 169.37 |
| ASIR | Southern Sub-Saharan Africa | Female | 1990 | 250.14 | 344.43 | 177.32 |
| ASIR | Southern Sub-Saharan Africa | Female | 2021 | 227.67 | 309.22 | 164.57 |
| ASIR | Southern Sub-Saharan Africa | Both | 1990 | 253.57 | 345.63 | 182.32 |
| ASIR | Southern Sub-Saharan Africa | Both | 2021 | 233.11 | 314.14 | 169.69 |
| ASIR | High-middle SDI | Male | 1990 | 618.55 | 849.26 | 441.80 |
| ASIR | High-middle SDI | Male | 2021 | 700.44 | 964.84 | 495.48 |
| ASIR | High-middle SDI | Female | 1990 | 983.73 | 1396.74 | 672.61 |
| ASIR | High-middle SDI | Female | 2021 | 987.60 | 1373.04 | 684.81 |
| ASIR | High-middle SDI | Both | 1990 | 869.35 | 1223.11 | 602.71 |
| ASIR | High-middle SDI | Both | 2021 | 886.87 | 1230.27 | 620.29 |
| ASIR | Western Sub-Saharan Africa | Male | 1990 | 280.97 | 376.52 | 206.12 |
| ASIR | Western Sub-Saharan Africa | Male | 2021 | 323.45 | 432.48 | 238.29 |
| ASIR | Western Sub-Saharan Africa | Female | 1990 | 441.60 | 618.94 | 308.17 |
| ASIR | Western Sub-Saharan Africa | Female | 2021 | 522.99 | 725.38 | 368.76 |
| ASIR | Western Sub-Saharan Africa | Both | 1990 | 371.26 | 511.41 | 264.15 |
| ASIR | Western Sub-Saharan Africa | Both | 2021 | 430.39 | 586.52 | 308.66 |
| ASIR | Middle SDI | Male | 1990 | 379.69 | 530.78 | 265.82 |
| ASIR | Middle SDI | Male | 2021 | 531.22 | 746.86 | 366.75 |
| ASIR | Middle SDI | Female | 1990 | 635.49 | 928.88 | 423.11 |
| ASIR | Middle SDI | Female | 2021 | 883.29 | 1278.17 | 587.80 |
| ASIR | Middle SDI | Both | 1990 | 528.70 | 759.54 | 358.47 |
| ASIR | Middle SDI | Both | 2021 | 738.37 | 1059.06 | 498.75 |
| ASIR | High SDI | Male | 1990 | 886.41 | 1217.19 | 622.95 |
| ASIR | High SDI | Male | 2021 | 1123.40 | 1540.52 | 794.43 |
| ASIR | High SDI | Female | 1990 | 1680.70 | 2317.47 | 1163.56 |
| ASIR | High SDI | Female | 2021 | 1836.20 | 2523.31 | 1282.76 |
| ASIR | High SDI | Both | 1990 | 1414.23 | 1940.30 | 985.61 |
| ASIR | High SDI | Both | 2021 | 1545.45 | 2115.88 | 1090.09 |
| ASIR | Low-middle SDI | Male | 1990 | 415.33 | 584.54 | 287.35 |
| ASIR | Low-middle SDI | Male | 2021 | 452.16 | 630.47 | 316.59 |
| ASIR | Low-middle SDI | Female | 1990 | 853.99 | 1281.51 | 549.98 |
| ASIR | Low-middle SDI | Female | 2021 | 968.50 | 1413.60 | 637.90 |
| ASIR | Low-middle SDI | Both | 1990 | 639.26 | 938.94 | 422.93 |
| ASIR | Low-middle SDI | Both | 2021 | 737.22 | 1059.25 | 497.52 |
| ASIR | Low SDI | Male | 1990 | 359.15 | 487.54 | 258.90 |
| ASIR | Low SDI | Male | 2021 | 402.47 | 546.84 | 291.43 |
| ASIR | Low SDI | Female | 1990 | 613.63 | 889.40 | 416.42 |
| ASIR | Low SDI | Female | 2021 | 747.32 | 1075.00 | 511.68 |
| ASIR | Low SDI | Both | 1990 | 491.52 | 692.28 | 342.02 |
| ASIR | Low SDI | Both | 2021 | 586.53 | 827.22 | 409.01 |
| ASPR | Global | Male | 1990 | 1055.61 | 1288.51 | 870.71 |
| ASPR | Global | Male | 2021 | 1341.32 | 1625.07 | 1104.87 |
| ASPR | Global | Female | 1990 | 2331.91 | 2863.52 | 1902.55 |
| ASPR | Global | Female | 2021 | 2517.15 | 3071.04 | 2058.34 |
| ASPR | Global | Both | 1990 | 1853.96 | 2266.47 | 1519.75 |
| ASPR | Global | Both | 2021 | 2037.39 | 2475.71 | 1670.75 |
| ASPR | East Asia | Male | 1990 | 489.80 | 607.06 | 399.73 |
| ASPR | East Asia | Male | 2021 | 868.70 | 1083.96 | 701.59 |
| ASPR | East Asia | Female | 1990 | 909.35 | 1151.41 | 725.89 |
| ASPR | East Asia | Female | 2021 | 1582.23 | 1989.38 | 1254.71 |
| ASPR | East Asia | Both | 1990 | 742.51 | 932.88 | 597.07 |
| ASPR | East Asia | Both | 2021 | 1304.52 | 1634.52 | 1040.54 |
| ASPR | Southeast Asia | Male | 1990 | 416.70 | 498.06 | 350.96 |
| ASPR | Southeast Asia | Male | 2021 | 557.88 | 688.94 | 458.34 |
| ASPR | Southeast Asia | Female | 1990 | 946.73 | 1191.04 | 761.87 |
| ASPR | Southeast Asia | Female | 2021 | 1161.10 | 1442.43 | 937.73 |
| ASPR | Southeast Asia | Both | 1990 | 727.02 | 900.01 | 593.55 |
| ASPR | Southeast Asia | Both | 2021 | 924.33 | 1142.79 | 751.65 |
| ASPR | Oceania | Male | 1990 | 203.72 | 233.65 | 177.34 |
| ASPR | Oceania | Male | 2021 | 248.89 | 284.92 | 217.44 |
| ASPR | Oceania | Female | 1990 | 942.83 | 1202.12 | 743.34 |
| ASPR | Oceania | Female | 2021 | 1517.11 | 1882.74 | 1208.85 |
| ASPR | Oceania | Both | 1990 | 592.37 | 738.14 | 477.91 |
| ASPR | Oceania | Both | 2021 | 899.28 | 1100.56 | 730.28 |
| ASPR | Central Asia | Male | 1990 | 399.79 | 461.53 | 348.74 |
| ASPR | Central Asia | Male | 2021 | 380.69 | 442.21 | 328.11 |
| ASPR | Central Asia | Female | 1990 | 465.77 | 551.06 | 394.56 |
| ASPR | Central Asia | Female | 2021 | 472.82 | 562.77 | 398.58 |
| ASPR | Central Asia | Both | 1990 | 447.92 | 523.57 | 384.73 |
| ASPR | Central Asia | Both | 2021 | 442.37 | 520.55 | 377.71 |
| ASPR | Central Europe | Male | 1990 | 1177.22 | 1424.70 | 975.07 |
| ASPR | Central Europe | Male | 2021 | 1344.50 | 1647.30 | 1096.03 |
| ASPR | Central Europe | Female | 1990 | 2458.41 | 3044.94 | 1975.25 |
| ASPR | Central Europe | Female | 2021 | 1887.03 | 2319.25 | 1536.64 |
| ASPR | Central Europe | Both | 1990 | 2024.84 | 2487.36 | 1642.17 |
| ASPR | Central Europe | Both | 2021 | 1722.46 | 2108.11 | 1408.01 |
| ASPR | Eastern Europe | Male | 1990 | 827.87 | 985.32 | 704.03 |
| ASPR | Eastern Europe | Male | 2021 | 903.15 | 1100.08 | 750.42 |
| ASPR | Eastern Europe | Female | 1990 | 958.05 | 1158.36 | 804.34 |
| ASPR | Eastern Europe | Female | 2021 | 950.18 | 1161.63 | 786.60 |
| ASPR | Eastern Europe | Both | 1990 | 941.07 | 1132.68 | 793.96 |
| ASPR | Eastern Europe | Both | 2021 | 955.04 | 1164.99 | 793.14 |
| ASPR | High-income Asia Pacific | Male | 1990 | 1889.19 | 2344.18 | 1533.15 |
| ASPR | High-income Asia Pacific | Male | 2021 | 2184.46 | 2661.58 | 1779.70 |
| ASPR | High-income Asia Pacific | Female | 1990 | 2550.44 | 3126.95 | 2076.23 |
| ASPR | High-income Asia Pacific | Female | 2021 | 2648.85 | 3195.15 | 2185.11 |
| ASPR | High-income Asia Pacific | Both | 1990 | 2338.74 | 2872.17 | 1901.59 |
| ASPR | High-income Asia Pacific | Both | 2021 | 2484.10 | 3004.15 | 2044.57 |
| ASPR | Western Europe | Male | 1990 | 2107.79 | 2555.48 | 1734.00 |
| ASPR | Western Europe | Male | 2021 | 2591.11 | 3113.89 | 2146.81 |
| ASPR | Western Europe | Female | 1990 | 4868.02 | 5950.16 | 3984.78 |
| ASPR | Western Europe | Female | 2021 | 5272.89 | 6337.86 | 4359.70 |
| ASPR | Western Europe | Both | 1990 | 3933.30 | 4775.21 | 3233.22 |
| ASPR | Western Europe | Both | 2021 | 4167.22 | 4990.36 | 3462.12 |
| ASPR | Australasia | Male | 1990 | 2127.31 | 2573.73 | 1746.59 |
| ASPR | Australasia | Male | 2021 | 3658.95 | 4384.45 | 3037.90 |
| ASPR | Australasia | Female | 1990 | 4410.38 | 5337.18 | 3616.14 |
| ASPR | Australasia | Female | 2021 | 6146.14 | 7320.51 | 5117.00 |
| ASPR | Australasia | Both | 1990 | 3587.46 | 4320.72 | 2959.91 |
| ASPR | Australasia | Both | 2021 | 5061.83 | 6019.44 | 4234.31 |
| ASPR | Southern Latin America | Male | 1990 | 1067.67 | 1246.36 | 914.46 |
| ASPR | Southern Latin America | Male | 2021 | 1254.91 | 1472.34 | 1069.32 |
| ASPR | Southern Latin America | Female | 1990 | 2002.27 | 2420.57 | 1646.73 |
| ASPR | Southern Latin America | Female | 2021 | 2372.69 | 2843.34 | 1961.90 |
| ASPR | Southern Latin America | Both | 1990 | 1646.69 | 1965.71 | 1375.21 |
| ASPR | Southern Latin America | Both | 2021 | 1952.88 | 2318.25 | 1635.25 |
| ASPR | High-income North America | Male | 1990 | 1858.91 | 2329.45 | 1491.54 |
| ASPR | High-income North America | Male | 2021 | 2963.48 | 3672.66 | 2371.35 |
| ASPR | High-income North America | Female | 1990 | 3444.22 | 4327.00 | 2735.53 |
| ASPR | High-income North America | Female | 2021 | 5545.11 | 6909.85 | 4428.17 |
| ASPR | High-income North America | Both | 1990 | 2859.19 | 3576.99 | 2283.71 |
| ASPR | High-income North America | Both | 2021 | 4419.63 | 5483.00 | 3540.43 |
| ASPR | Caribbean | Male | 1990 | 565.42 | 665.32 | 480.83 |
| ASPR | Caribbean | Male | 2021 | 812.87 | 974.80 | 678.14 |
| ASPR | Caribbean | Female | 1990 | 1261.22 | 1546.44 | 1030.61 |
| ASPR | Caribbean | Female | 2021 | 1919.87 | 2330.59 | 1561.28 |
| ASPR | Caribbean | Both | 1990 | 943.14 | 1140.36 | 782.15 |
| ASPR | Caribbean | Both | 2021 | 1447.19 | 1745.84 | 1188.21 |
| ASPR | Andean Latin America | Male | 1990 | 476.04 | 542.51 | 418.29 |
| ASPR | Andean Latin America | Male | 2021 | 587.05 | 692.48 | 503.91 |
| ASPR | Andean Latin America | Female | 1990 | 527.30 | 618.83 | 452.29 |
| ASPR | Andean Latin America | Female | 2021 | 689.75 | 825.00 | 578.11 |
| ASPR | Andean Latin America | Both | 1990 | 505.68 | 585.62 | 439.89 |
| ASPR | Andean Latin America | Both | 2021 | 646.91 | 766.98 | 548.89 |
| ASPR | Central Latin America | Male | 1990 | 901.69 | 1073.86 | 767.14 |
| ASPR | Central Latin America | Male | 2021 | 742.49 | 890.27 | 626.04 |
| ASPR | Central Latin America | Female | 1990 | 1320.28 | 1648.23 | 1064.60 |
| ASPR | Central Latin America | Female | 2021 | 1055.36 | 1296.49 | 860.37 |
| ASPR | Central Latin America | Both | 1990 | 1123.60 | 1376.00 | 927.54 |
| ASPR | Central Latin America | Both | 2021 | 924.21 | 1122.89 | 765.20 |
| ASPR | Tropical Latin America | Male | 1990 | 795.58 | 963.46 | 665.14 |
| ASPR | Tropical Latin America | Male | 2021 | 873.64 | 1055.75 | 730.42 |
| ASPR | Tropical Latin America | Female | 1990 | 1156.59 | 1458.39 | 929.16 |
| ASPR | Tropical Latin America | Female | 2021 | 1370.30 | 1712.34 | 1098.76 |
| ASPR | Tropical Latin America | Both | 1990 | 1010.04 | 1252.81 | 824.90 |
| ASPR | Tropical Latin America | Both | 2021 | 1179.26 | 1455.69 | 961.55 |
| ASPR | North Africa and Middle East | Male | 1990 | 467.87 | 540.17 | 408.71 |
| ASPR | North Africa and Middle East | Male | 2021 | 617.08 | 765.95 | 515.37 |
| ASPR | North Africa and Middle East | Female | 1990 | 638.92 | 765.26 | 540.02 |
| ASPR | North Africa and Middle East | Female | 2021 | 982.95 | 1194.06 | 811.36 |
| ASPR | North Africa and Middle East | Both | 1990 | 560.81 | 660.71 | 481.83 |
| ASPR | North Africa and Middle East | Both | 2021 | 804.89 | 983.91 | 669.47 |
| ASPR | South Asia | Male | 1990 | 555.67 | 714.22 | 436.37 |
| ASPR | South Asia | Male | 2021 | 758.04 | 958.64 | 601.51 |
| ASPR | South Asia | Female | 1990 | 1617.83 | 2161.71 | 1216.40 |
| ASPR | South Asia | Female | 2021 | 2214.47 | 2883.50 | 1704.02 |
| ASPR | South Asia | Both | 1990 | 1073.78 | 1418.56 | 818.03 |
| ASPR | South Asia | Both | 2021 | 1544.17 | 1993.79 | 1198.20 |
| ASPR | Central Sub-Saharan Africa | Male | 1990 | 278.23 | 333.65 | 233.45 |
| ASPR | Central Sub-Saharan Africa | Male | 2021 | 354.23 | 432.75 | 294.68 |
| ASPR | Central Sub-Saharan Africa | Female | 1990 | 467.32 | 568.95 | 384.83 |
| ASPR | Central Sub-Saharan Africa | Female | 2021 | 590.69 | 718.92 | 482.57 |
| ASPR | Central Sub-Saharan Africa | Both | 1990 | 378.38 | 457.12 | 314.77 |
| ASPR | Central Sub-Saharan Africa | Both | 2021 | 504.68 | 613.66 | 414.99 |
| ASPR | Eastern Sub-Saharan Africa | Male | 1990 | 303.29 | 366.81 | 250.18 |
| ASPR | Eastern Sub-Saharan Africa | Male | 2021 | 388.74 | 479.08 | 320.14 |
| ASPR | Eastern Sub-Saharan Africa | Female | 1990 | 511.85 | 629.34 | 419.38 |
| ASPR | Eastern Sub-Saharan Africa | Female | 2021 | 660.97 | 820.16 | 536.85 |
| ASPR | Eastern Sub-Saharan Africa | Both | 1990 | 414.49 | 506.51 | 341.11 |
| ASPR | Eastern Sub-Saharan Africa | Both | 2021 | 545.27 | 675.34 | 445.87 |
| ASPR | Southern Sub-Saharan Africa | Male | 1990 | 401.13 | 456.86 | 351.01 |
| ASPR | Southern Sub-Saharan Africa | Male | 2021 | 285.59 | 333.09 | 244.20 |
| ASPR | Southern Sub-Saharan Africa | Female | 1990 | 477.34 | 559.13 | 410.38 |
| ASPR | Southern Sub-Saharan Africa | Female | 2021 | 324.96 | 388.69 | 273.28 |
| ASPR | Southern Sub-Saharan Africa | Both | 1990 | 453.18 | 525.01 | 392.33 |
| ASPR | Southern Sub-Saharan Africa | Both | 2021 | 315.66 | 373.41 | 267.46 |
| ASPR | High-middle SDI | Male | 1990 | 1049.94 | 1273.73 | 872.27 |
| ASPR | High-middle SDI | Male | 2021 | 1172.40 | 1422.85 | 969.27 |
| ASPR | High-middle SDI | Female | 1990 | 1916.33 | 2358.26 | 1560.41 |
| ASPR | High-middle SDI | Female | 2021 | 1997.07 | 2435.71 | 1635.87 |
| ASPR | High-middle SDI | Both | 1990 | 1628.54 | 1990.63 | 1334.82 |
| ASPR | High-middle SDI | Both | 2021 | 1692.48 | 2056.85 | 1392.19 |
| ASPR | Western Sub-Saharan Africa | Male | 1990 | 318.07 | 385.18 | 263.28 |
| ASPR | Western Sub-Saharan Africa | Male | 2021 | 401.27 | 485.59 | 333.11 |
| ASPR | Western Sub-Saharan Africa | Female | 1990 | 594.28 | 745.53 | 478.32 |
| ASPR | Western Sub-Saharan Africa | Female | 2021 | 767.39 | 953.75 | 617.06 |
| ASPR | Western Sub-Saharan Africa | Both | 1990 | 472.11 | 584.23 | 383.74 |
| ASPR | Western Sub-Saharan Africa | Both | 2021 | 597.49 | 735.14 | 486.18 |
| ASPR | Middle SDI | Male | 1990 | 537.67 | 652.55 | 447.99 |
| ASPR | Middle SDI | Male | 2021 | 760.90 | 937.42 | 621.32 |
| ASPR | Middle SDI | Female | 1990 | 1007.23 | 1274.51 | 805.37 |
| ASPR | Middle SDI | Female | 2021 | 1479.95 | 1870.64 | 1174.21 |
| ASPR | Middle SDI | Both | 1990 | 808.52 | 1009.57 | 656.99 |
| ASPR | Middle SDI | Both | 2021 | 1180.98 | 1477.90 | 947.82 |
| ASPR | High SDI | Male | 1990 | 1862.71 | 2284.97 | 1523.29 |
| ASPR | High SDI | Male | 2021 | 2523.61 | 3066.61 | 2061.35 |
| ASPR | High SDI | Female | 1990 | 3794.75 | 4654.68 | 3101.61 |
| ASPR | High SDI | Female | 2021 | 4529.09 | 5507.16 | 3704.11 |
| ASPR | High SDI | Both | 1990 | 3128.04 | 3827.30 | 2563.50 |
| ASPR | High SDI | Both | 2021 | 3690.83 | 4474.90 | 3029.71 |
| ASPR | Low-middle SDI | Male | 1990 | 489.40 | 608.63 | 398.39 |
| ASPR | Low-middle SDI | Male | 2021 | 621.79 | 768.91 | 506.93 |
| ASPR | Low-middle SDI | Female | 1990 | 1154.92 | 1513.87 | 891.22 |
| ASPR | Low-middle SDI | Female | 2021 | 1508.94 | 1933.88 | 1178.36 |
| ASPR | Low-middle SDI | Both | 1990 | 828.48 | 1068.99 | 650.87 |
| ASPR | Low-middle SDI | Both | 2021 | 1111.07 | 1407.87 | 879.41 |
| ASPR | Low SDI | Male | 1990 | 378.87 | 468.34 | 308.35 |
| ASPR | Low SDI | Male | 2021 | 509.47 | 635.06 | 413.33 |
| ASPR | Low SDI | Female | 1990 | 795.25 | 1020.77 | 622.65 |
| ASPR | Low SDI | Female | 2021 | 1131.65 | 1445.46 | 890.76 |
| ASPR | Low SDI | Both | 1990 | 594.27 | 752.67 | 473.24 |
| ASPR | Low SDI | Both | 2021 | 841.42 | 1063.20 | 668.81 |
| ASYR | Global | Male | 1990 | 131.88 | 181.68 | 90.33 |
| ASYR | Global | Male | 2021 | 124.50 | 174.31 | 84.35 |
| ASYR | Global | Female | 1990 | 261.27 | 363.53 | 178.38 |
| ASYR | Global | Female | 2021 | 228.33 | 319.51 | 154.29 |
| ASYR | Global | Both | 1990 | 212.08 | 293.83 | 144.85 |
| ASYR | Global | Both | 2021 | 185.49 | 259.43 | 125.69 |
| ASYR | East Asia | Male | 1990 | 95.93 | 135.34 | 64.61 |
| ASYR | East Asia | Male | 2021 | 79.68 | 117.02 | 51.55 |
| ASYR | East Asia | Female | 1990 | 171.95 | 241.00 | 115.35 |
| ASYR | East Asia | Female | 2021 | 131.75 | 190.44 | 85.72 |
| ASYR | East Asia | Both | 1990 | 141.55 | 198.40 | 95.08 |
| ASYR | East Asia | Both | 2021 | 111.44 | 161.70 | 72.39 |
| ASYR | Southeast Asia | Male | 1990 | 90.70 | 125.00 | 61.24 |
| ASYR | Southeast Asia | Male | 2021 | 85.26 | 119.27 | 57.91 |
| ASYR | Southeast Asia | Female | 1990 | 201.98 | 283.01 | 135.31 |
| ASYR | Southeast Asia | Female | 2021 | 172.08 | 239.08 | 115.68 |
| ASYR | Southeast Asia | Both | 1990 | 155.58 | 216.43 | 104.78 |
| ASYR | Southeast Asia | Both | 2021 | 137.83 | 191.29 | 93.01 |
| ASYR | Oceania | Male | 1990 | 46.77 | 66.27 | 30.75 |
| ASYR | Oceania | Male | 2021 | 52.15 | 73.53 | 34.24 |
| ASYR | Oceania | Female | 1990 | 215.12 | 308.48 | 140.90 |
| ASYR | Oceania | Female | 2021 | 319.35 | 449.07 | 209.43 |
| ASYR | Oceania | Both | 1990 | 134.92 | 190.67 | 89.32 |
| ASYR | Oceania | Both | 2021 | 188.75 | 262.72 | 125.43 |
| ASYR | Central Asia | Male | 1990 | 74.54 | 101.07 | 51.07 |
| ASYR | Central Asia | Male | 2021 | 56.90 | 77.98 | 38.89 |
| ASYR | Central Asia | Female | 1990 | 83.71 | 113.37 | 57.78 |
| ASYR | Central Asia | Female | 2021 | 65.79 | 89.04 | 44.90 |
| ASYR | Central Asia | Both | 1990 | 81.51 | 109.58 | 56.48 |
| ASYR | Central Asia | Both | 2021 | 63.12 | 85.56 | 43.62 |
| ASYR | Central Europe | Male | 1990 | 174.70 | 241.48 | 118.41 |
| ASYR | Central Europe | Male | 2021 | 114.53 | 165.82 | 75.02 |
| ASYR | Central Europe | Female | 1990 | 340.22 | 471.44 | 229.50 |
| ASYR | Central Europe | Female | 2021 | 149.06 | 214.27 | 97.73 |
| ASYR | Central Europe | Both | 1990 | 284.45 | 393.28 | 191.89 |
| ASYR | Central Europe | Both | 2021 | 139.39 | 200.32 | 91.47 |
| ASYR | Eastern Europe | Male | 1990 | 119.86 | 162.47 | 82.90 |
| ASYR | Eastern Europe | Male | 2021 | 87.30 | 123.04 | 58.25 |
| ASYR | Eastern Europe | Female | 1990 | 133.34 | 181.86 | 92.59 |
| ASYR | Eastern Europe | Female | 2021 | 84.48 | 118.15 | 57.30 |
| ASYR | Eastern Europe | Both | 1990 | 132.48 | 179.19 | 91.67 |
| ASYR | Eastern Europe | Both | 2021 | 87.57 | 122.49 | 59.11 |
| ASYR | High-income Asia Pacific | Male | 1990 | 151.00 | 212.03 | 101.78 |
| ASYR | High-income Asia Pacific | Male | 2021 | 147.87 | 212.03 | 96.39 |
| ASYR | High-income Asia Pacific | Female | 1990 | 206.26 | 289.28 | 140.11 |
| ASYR | High-income Asia Pacific | Female | 2021 | 176.16 | 251.22 | 115.50 |
| ASYR | High-income Asia Pacific | Both | 1990 | 188.18 | 263.25 | 127.37 |
| ASYR | High-income Asia Pacific | Both | 2021 | 166.39 | 237.09 | 108.88 |
| ASYR | Western Europe | Male | 1990 | 173.64 | 243.41 | 116.62 |
| ASYR | Western Europe | Male | 2021 | 181.87 | 260.23 | 120.88 |
| ASYR | Western Europe | Female | 1990 | 395.84 | 554.94 | 266.71 |
| ASYR | Western Europe | Female | 2021 | 362.04 | 515.50 | 239.89 |
| ASYR | Western Europe | Both | 1990 | 320.55 | 448.29 | 216.54 |
| ASYR | Western Europe | Both | 2021 | 287.85 | 409.67 | 191.46 |
| ASYR | Australasia | Male | 1990 | 156.84 | 222.36 | 103.53 |
| ASYR | Australasia | Male | 2021 | 264.68 | 375.76 | 176.50 |
| ASYR | Australasia | Female | 1990 | 313.87 | 448.52 | 208.43 |
| ASYR | Australasia | Female | 2021 | 434.03 | 618.46 | 289.16 |
| ASYR | Australasia | Both | 1990 | 257.31 | 366.27 | 171.46 |
| ASYR | Australasia | Both | 2021 | 360.23 | 512.81 | 241.05 |
| ASYR | Southern Latin America | Male | 1990 | 179.79 | 245.29 | 123.27 |
| ASYR | Southern Latin America | Male | 2021 | 123.65 | 168.13 | 85.46 |
| ASYR | Southern Latin America | Female | 1990 | 333.34 | 458.99 | 227.78 |
| ASYR | Southern Latin America | Female | 2021 | 236.44 | 324.73 | 160.94 |
| ASYR | Southern Latin America | Both | 1990 | 274.85 | 375.65 | 188.63 |
| ASYR | Southern Latin America | Both | 2021 | 193.89 | 265.05 | 133.19 |
| ASYR | High-income North America | Male | 1990 | 137.79 | 197.20 | 90.34 |
| ASYR | High-income North America | Male | 2021 | 203.36 | 291.87 | 132.44 |
| ASYR | High-income North America | Female | 1990 | 246.21 | 356.19 | 161.14 |
| ASYR | High-income North America | Female | 2021 | 366.12 | 526.23 | 235.91 |
| ASYR | High-income North America | Both | 1990 | 206.13 | 297.29 | 135.34 |
| ASYR | High-income North America | Both | 2021 | 295.10 | 423.80 | 190.75 |
| ASYR | Caribbean | Male | 1990 | 95.55 | 131.19 | 64.90 |
| ASYR | Caribbean | Male | 2021 | 98.09 | 136.76 | 65.95 |
| ASYR | Caribbean | Female | 1990 | 197.50 | 273.39 | 132.11 |
| ASYR | Caribbean | Female | 2021 | 208.96 | 296.16 | 138.90 |
| ASYR | Caribbean | Both | 1990 | 150.93 | 207.41 | 102.16 |
| ASYR | Caribbean | Both | 2021 | 161.55 | 226.10 | 108.36 |
| ASYR | Andean Latin America | Male | 1990 | 108.21 | 149.31 | 73.08 |
| ASYR | Andean Latin America | Male | 2021 | 82.73 | 114.01 | 56.68 |
| ASYR | Andean Latin America | Female | 1990 | 117.83 | 162.61 | 79.56 |
| ASYR | Andean Latin America | Female | 2021 | 95.47 | 131.29 | 65.07 |
| ASYR | Andean Latin America | Both | 1990 | 113.87 | 154.04 | 77.70 |
| ASYR | Andean Latin America | Both | 2021 | 90.20 | 123.14 | 62.50 |
| ASYR | Central Latin America | Male | 1990 | 183.37 | 250.01 | 126.63 |
| ASYR | Central Latin America | Male | 2021 | 105.87 | 145.06 | 72.82 |
| ASYR | Central Latin America | Female | 1990 | 260.78 | 363.07 | 176.69 |
| ASYR | Central Latin America | Female | 2021 | 145.22 | 200.05 | 98.90 |
| ASYR | Central Latin America | Both | 1990 | 224.47 | 310.03 | 153.00 |
| ASYR | Central Latin America | Both | 2021 | 128.79 | 177.04 | 88.53 |
| ASYR | Tropical Latin America | Male | 1990 | 161.60 | 221.44 | 110.65 |
| ASYR | Tropical Latin America | Male | 2021 | 125.43 | 171.65 | 85.99 |
| ASYR | Tropical Latin America | Female | 1990 | 229.01 | 320.68 | 155.09 |
| ASYR | Tropical Latin America | Female | 2021 | 190.66 | 265.47 | 129.59 |
| ASYR | Tropical Latin America | Both | 1990 | 201.72 | 279.94 | 137.48 |
| ASYR | Tropical Latin America | Both | 2021 | 165.60 | 228.85 | 113.45 |
| ASYR | North Africa and Middle East | Male | 1990 | 96.74 | 130.54 | 66.69 |
| ASYR | North Africa and Middle East | Male | 2021 | 74.54 | 103.51 | 51.18 |
| ASYR | North Africa and Middle East | Female | 1990 | 128.37 | 175.16 | 87.42 |
| ASYR | North Africa and Middle East | Female | 2021 | 113.19 | 155.60 | 76.58 |
| ASYR | North Africa and Middle East | Both | 1990 | 113.99 | 153.29 | 78.07 |
| ASYR | North Africa and Middle East | Both | 2021 | 94.43 | 129.97 | 64.41 |
| ASYR | South Asia | Male | 1990 | 134.68 | 193.48 | 88.53 |
| ASYR | South Asia | Male | 2021 | 135.88 | 191.12 | 90.49 |
| ASYR | South Asia | Female | 1990 | 380.27 | 554.32 | 245.33 |
| ASYR | South Asia | Female | 2021 | 380.76 | 538.77 | 250.61 |
| ASYR | South Asia | Both | 1990 | 254.41 | 368.20 | 165.22 |
| ASYR | South Asia | Both | 2021 | 267.74 | 377.24 | 176.84 |
| ASYR | Central Sub-Saharan Africa | Male | 1990 | 73.49 | 104.69 | 47.96 |
| ASYR | Central Sub-Saharan Africa | Male | 2021 | 80.45 | 114.37 | 52.87 |
| ASYR | Central Sub-Saharan Africa | Female | 1990 | 121.14 | 171.14 | 79.86 |
| ASYR | Central Sub-Saharan Africa | Female | 2021 | 131.96 | 185.14 | 87.43 |
| ASYR | Central Sub-Saharan Africa | Both | 1990 | 98.70 | 138.78 | 66.13 |
| ASYR | Central Sub-Saharan Africa | Both | 2021 | 113.17 | 157.26 | 76.43 |
| ASYR | Eastern Sub-Saharan Africa | Male | 1990 | 78.79 | 110.58 | 53.16 |
| ASYR | Eastern Sub-Saharan Africa | Male | 2021 | 86.00 | 119.96 | 57.28 |
| ASYR | Eastern Sub-Saharan Africa | Female | 1990 | 129.72 | 180.79 | 87.40 |
| ASYR | Eastern Sub-Saharan Africa | Female | 2021 | 141.35 | 196.03 | 95.02 |
| ASYR | Eastern Sub-Saharan Africa | Both | 1990 | 105.87 | 147.10 | 71.40 |
| ASYR | Eastern Sub-Saharan Africa | Both | 2021 | 117.81 | 163.47 | 79.12 |
| ASYR | Southern Sub-Saharan Africa | Male | 1990 | 85.71 | 116.10 | 58.30 |
| ASYR | Southern Sub-Saharan Africa | Male | 2021 | 52.88 | 72.03 | 36.15 |
| ASYR | Southern Sub-Saharan Africa | Female | 1990 | 99.59 | 134.93 | 68.14 |
| ASYR | Southern Sub-Saharan Africa | Female | 2021 | 59.17 | 80.89 | 40.07 |
| ASYR | Southern Sub-Saharan Africa | Both | 1990 | 95.35 | 128.43 | 65.42 |
| ASYR | Southern Sub-Saharan Africa | Both | 2021 | 57.80 | 78.64 | 39.51 |
| ASYR | High-middle SDI | Male | 1990 | 131.14 | 179.52 | 90.05 |
| ASYR | High-middle SDI | Male | 2021 | 97.47 | 139.17 | 64.72 |
| ASYR | High-middle SDI | Female | 1990 | 219.68 | 304.35 | 150.43 |
| ASYR | High-middle SDI | Female | 2021 | 155.42 | 220.62 | 103.54 |
| ASYR | High-middle SDI | Both | 1990 | 190.08 | 262.46 | 130.06 |
| ASYR | High-middle SDI | Both | 2021 | 134.07 | 190.24 | 89.42 |
| ASYR | Western Sub-Saharan Africa | Male | 1990 | 78.16 | 108.50 | 52.45 |
| ASYR | Western Sub-Saharan Africa | Male | 2021 | 84.31 | 116.68 | 56.69 |
| ASYR | Western Sub-Saharan Africa | Female | 1990 | 141.49 | 198.65 | 94.71 |
| ASYR | Western Sub-Saharan Africa | Female | 2021 | 153.54 | 213.57 | 102.84 |
| ASYR | Western Sub-Saharan Africa | Both | 1990 | 113.44 | 158.44 | 76.07 |
| ASYR | Western Sub-Saharan Africa | Both | 2021 | 121.38 | 168.11 | 81.55 |
| ASYR | Middle SDI | Male | 1990 | 112.26 | 156.17 | 76.17 |
| ASYR | Middle SDI | Male | 2021 | 94.39 | 132.56 | 63.94 |
| ASYR | Middle SDI | Female | 1990 | 206.49 | 288.84 | 138.38 |
| ASYR | Middle SDI | Female | 2021 | 180.41 | 253.46 | 121.07 |
| ASYR | Middle SDI | Both | 1990 | 166.47 | 232.23 | 111.91 |
| ASYR | Middle SDI | Both | 2021 | 144.38 | 202.80 | 97.29 |
| ASYR | High SDI | Male | 1990 | 156.78 | 220.43 | 105.38 |
| ASYR | High SDI | Male | 2021 | 177.29 | 253.82 | 116.87 |
| ASYR | High SDI | Female | 1990 | 313.13 | 438.21 | 211.88 |
| ASYR | High SDI | Female | 2021 | 308.23 | 440.40 | 203.02 |
| ASYR | High SDI | Both | 1990 | 259.21 | 362.45 | 175.35 |
| ASYR | High SDI | Both | 2021 | 253.65 | 362.28 | 167.45 |
| ASYR | Low-middle SDI | Male | 1990 | 117.72 | 166.08 | 78.67 |
| ASYR | Low-middle SDI | Male | 2021 | 115.15 | 160.57 | 77.33 |
| ASYR | Low-middle SDI | Female | 1990 | 272.43 | 392.10 | 177.80 |
| ASYR | Low-middle SDI | Female | 2021 | 273.07 | 384.56 | 180.91 |
| ASYR | Low-middle SDI | Both | 1990 | 196.44 | 280.03 | 129.40 |
| ASYR | Low-middle SDI | Both | 2021 | 202.08 | 282.71 | 134.82 |
| ASYR | Low SDI | Male | 1990 | 97.91 | 137.78 | 65.63 |
| ASYR | Low SDI | Male | 2021 | 107.97 | 151.46 | 72.55 |
| ASYR | Low SDI | Female | 1990 | 200.35 | 283.69 | 131.84 |
| ASYR | Low SDI | Female | 2021 | 230.09 | 323.14 | 153.07 |
| ASYR | Low SDI | Both | 1990 | 150.82 | 212.57 | 100.11 |
| ASYR | Low SDI | Both | 2021 | 173.10 | 242.18 | 115.28 |

Abbreviations: ASIR, age-standardized incidence rate; ASPR, age-standardized prevalence rate; ASYR, age-standardized years lived with disability rate; HFs, Hip fractures; SDI, sociodemographic index; GBD, Global Burden of Disease Study; UI, uncertainty interval.

**Table S2. The results of new cases, prevalence count, and YLDs for HFs (≥55 years) across global, 5 SDI regions, and 21 GBD regions**

| **measure** | **location** | **sex** | **year** | **value** | **95% UI_upper** | **95% UI_lower** |
| --- | --- | --- | --- | --- | --- | --- |
| new cases | Andean Latin America | Both | 1990 | 9523 | 12753 | 7046 |
| new cases | Andean Latin America | Both | 2021 | 34935 | 46832 | 25795 |
| new cases | Andean Latin America | Female | 1990 | 4859 | 6503 | 3573 |
| new cases | Andean Latin America | Female | 2021 | 19556 | 26537 | 14246 |
| new cases | Andean Latin America | Male | 1990 | 4665 | 6297 | 3438 |
| new cases | Andean Latin America | Male | 2021 | 15379 | 20570 | 11417 |
| new cases | Australasia | Both | 1990 | 65187 | 88033 | 46051 |
| new cases | Australasia | Both | 2021 | 262375 | 346575 | 189442 |
| new cases | Australasia | Female | 1990 | 49055 | 66860 | 34435 |
| new cases | Australasia | Female | 2021 | 178755 | 237847 | 127741 |
| new cases | Australasia | Male | 1990 | 16132 | 21684 | 11556 |
| new cases | Australasia | Male | 2021 | 83620 | 111077 | 59919 |
| new cases | Caribbean | Both | 1990 | 24093 | 32826 | 17227 |
| new cases | Caribbean | Both | 2021 | 86758 | 116458 | 62421 |
| new cases | Caribbean | Female | 1990 | 16532 | 22877 | 11544 |
| new cases | Caribbean | Female | 2021 | 63412 | 85372 | 44958 |
| new cases | Caribbean | Male | 1990 | 7561 | 10180 | 5594 |
| new cases | Caribbean | Male | 2021 | 23346 | 31366 | 16944 |
| new cases | Central Asia | Both | 1990 | 19091 | 25892 | 14052 |
| new cases | Central Asia | Both | 2021 | 35079 | 47286 | 25834 |
| new cases | Central Asia | Female | 1990 | 10928 | 14960 | 7936 |
| new cases | Central Asia | Female | 2021 | 19495 | 26569 | 14117 |
| new cases | Central Asia | Male | 1990 | 8163 | 11177 | 5996 |
| new cases | Central Asia | Male | 2021 | 15584 | 21137 | 11489 |
| new cases | Central Europe | Both | 1990 | 322635 | 446617 | 228741 |
| new cases | Central Europe | Both | 2021 | 437950 | 605906 | 309188 |
| new cases | Central Europe | Female | 1990 | 229157 | 319862 | 160797 |
| new cases | Central Europe | Female | 2021 | 287131 | 394296 | 200724 |
| new cases | Central Europe | Male | 1990 | 93478 | 128553 | 67263 |
| new cases | Central Europe | Male | 2021 | 150819 | 209794 | 106896 |
| new cases | Central Latin America | Both | 1990 | 80092 | 111834 | 56339 |
| new cases | Central Latin America | Both | 2021 | 212004 | 290933 | 151439 |
| new cases | Central Latin America | Female | 1990 | 46143 | 66082 | 31583 |
| new cases | Central Latin America | Female | 2021 | 128853 | 179133 | 90265 |
| new cases | Central Latin America | Male | 1990 | 33949 | 46503 | 24423 |
| new cases | Central Latin America | Male | 2021 | 83151 | 112230 | 60539 |
| new cases | Central Sub-Saharan Africa | Both | 1990 | 8422 | 11295 | 6153 |
| new cases | Central Sub-Saharan Africa | Both | 2021 | 22331 | 29921 | 16201 |
| new cases | Central Sub-Saharan Africa | Female | 1990 | 4754 | 6474 | 3410 |
| new cases | Central Sub-Saharan Africa | Female | 2021 | 14122 | 19234 | 10107 |
| new cases | Central Sub-Saharan Africa | Male | 1990 | 3668 | 4848 | 2720 |
| new cases | Central Sub-Saharan Africa | Male | 2021 | 8209 | 10867 | 6135 |
| new cases | East Asia | Both | 1990 | 507068 | 751413 | 334944 |
| new cases | East Asia | Both | 2021 | 2611289 | 3806885 | 1730048 |
| new cases | East Asia | Female | 1990 | 318084 | 476329 | 206834 |
| new cases | East Asia | Female | 2021 | 1664581 | 2440804 | 1091617 |
| new cases | East Asia | Male | 1990 | 188984 | 273670 | 126538 |
| new cases | East Asia | Male | 2021 | 946708 | 1363803 | 637343 |
| new cases | Eastern Europe | Both | 1990 | 250588 | 349564 | 176734 |
| new cases | Eastern Europe | Both | 2021 | 330075 | 456447 | 232574 |
| new cases | Eastern Europe | Female | 1990 | 156607 | 220552 | 109486 |
| new cases | Eastern Europe | Female | 2021 | 192418 | 268074 | 135367 |
| new cases | Eastern Europe | Male | 1990 | 93980 | 129651 | 66917 |
| new cases | Eastern Europe | Male | 2021 | 137656 | 189677 | 96790 |
| new cases | Eastern Sub-Saharan Africa | Both | 1990 | 37442 | 50557 | 27629 |
| new cases | Eastern Sub-Saharan Africa | Both | 2021 | 84926 | 114071 | 62313 |
| new cases | Eastern Sub-Saharan Africa | Female | 1990 | 20183 | 27754 | 14590 |
| new cases | Eastern Sub-Saharan Africa | Female | 2021 | 51098 | 69628 | 36716 |
| new cases | Eastern Sub-Saharan Africa | Male | 1990 | 17260 | 23163 | 12761 |
| new cases | Eastern Sub-Saharan Africa | Male | 2021 | 33829 | 44740 | 25224 |
| new cases | Global | Both | 1990 | 5247600 | 7338866 | 3646909 |
| new cases | Global | Both | 2021 | 14099256 | 19487520 | 9860477 |
| new cases | Global | Female | 1990 | 3770164 | 5315715 | 2591506 |
| new cases | Global | Female | 2021 | 9721359 | 13483764 | 6743037 |
| new cases | Global | Male | 1990 | 1477436 | 2034049 | 1053798 |
| new cases | Global | Male | 2021 | 4377898 | 6036521 | 3105629 |
| new cases | High SDI | Both | 1990 | 2661249 | 3656847 | 1851963 |
| new cases | High SDI | Both | 2021 | 6469340 | 8792492 | 4589724 |
| new cases | High SDI | Female | 1990 | 2038504 | 2805578 | 1414170 |
| new cases | High SDI | Female | 2021 | 4577217 | 6207043 | 3235527 |
| new cases | High SDI | Male | 1990 | 622745 | 859365 | 436050 |
| new cases | High SDI | Male | 2021 | 1892123 | 2594912 | 1338087 |
| new cases | High-income Asia Pacific | Both | 1990 | 271212 | 394945 | 179033 |
| new cases | High-income Asia Pacific | Both | 2021 | 912551 | 1270355 | 632025 |
| new cases | High-income Asia Pacific | Female | 1990 | 177395 | 259053 | 116412 |
| new cases | High-income Asia Pacific | Female | 2021 | 597334 | 829338 | 412729 |
| new cases | High-income Asia Pacific | Male | 1990 | 93817 | 136783 | 62559 |
| new cases | High-income Asia Pacific | Male | 2021 | 315217 | 443015 | 216141 |
| new cases | High-income North America | Both | 1990 | 715537 | 1054934 | 465215 |
| new cases | High-income North America | Both | 2021 | 2070979 | 2955195 | 1372755 |
| new cases | High-income North America | Female | 1990 | 521657 | 772073 | 336818 |
| new cases | High-income North America | Female | 2021 | 1420755 | 2030750 | 939213 |
| new cases | High-income North America | Male | 1990 | 193879 | 283996 | 128221 |
| new cases | High-income North America | Male | 2021 | 650224 | 925475 | 435961 |
| new cases | High-middle SDI | Both | 1990 | 1226641 | 1727506 | 851790 |
| new cases | High-middle SDI | Both | 2021 | 2899320 | 4028639 | 2026564 |
| new cases | High-middle SDI | Female | 1990 | 857973 | 1220216 | 586745 |
| new cases | High-middle SDI | Female | 2021 | 1952599 | 2711456 | 1354870 |
| new cases | High-middle SDI | Male | 1990 | 368667 | 505085 | 263791 |
| new cases | High-middle SDI | Male | 2021 | 946721 | 1308272 | 668647 |
| new cases | Low SDI | Both | 1990 | 140672 | 197141 | 98390 |
| new cases | Low SDI | Both | 2021 | 375714 | 531651 | 261576 |
| new cases | Low SDI | Female | 1990 | 84676 | 122710 | 57488 |
| new cases | Low SDI | Female | 2021 | 245883 | 355182 | 167677 |
| new cases | Low SDI | Male | 1990 | 55996 | 75704 | 40568 |
| new cases | Low SDI | Male | 2021 | 129831 | 176140 | 94010 |
| new cases | Low-middle SDI | Both | 1990 | 511498 | 751485 | 338548 |
| new cases | Low-middle SDI | Both | 2021 | 1460164 | 2102850 | 984054 |
| new cases | Low-middle SDI | Female | 1990 | 335616 | 504941 | 215465 |
| new cases | Low-middle SDI | Female | 2021 | 1025495 | 1502531 | 673038 |
| new cases | Low-middle SDI | Male | 1990 | 175882 | 247009 | 121874 |
| new cases | Low-middle SDI | Male | 2021 | 434669 | 606015 | 304430 |
| new cases | Middle SDI | Both | 1990 | 699606 | 1005922 | 474982 |
| new cases | Middle SDI | Both | 2021 | 2880940 | 4142451 | 1943137 |
| new cases | Middle SDI | Female | 1990 | 447646 | 656066 | 297653 |
| new cases | Middle SDI | Female | 2021 | 1910578 | 2770737 | 1269466 |
| new cases | Middle SDI | Male | 1990 | 251960 | 351516 | 176658 |
| new cases | Middle SDI | Male | 2021 | 970362 | 1367060 | 668381 |
| new cases | North Africa and Middle East | Both | 1990 | 84808 | 111900 | 62471 |
| new cases | North Africa and Middle East | Both | 2021 | 287930 | 384785 | 209832 |
| new cases | North Africa and Middle East | Female | 1990 | 44407 | 59448 | 32139 |
| new cases | North Africa and Middle East | Female | 2021 | 170076 | 231483 | 120801 |
| new cases | North Africa and Middle East | Male | 1990 | 40400 | 53132 | 29706 |
| new cases | North Africa and Middle East | Male | 2021 | 117854 | 154063 | 87746 |
| new cases | Oceania | Both | 1990 | 1264 | 1767 | 886 |
| new cases | Oceania | Both | 2021 | 4718 | 6572 | 3294 |
| new cases | Oceania | Female | 1990 | 939 | 1368 | 626 |
| new cases | Oceania | Female | 2021 | 3797 | 5436 | 2543 |
| new cases | Oceania | Male | 1990 | 324 | 422 | 244 |
| new cases | Oceania | Male | 2021 | 922 | 1193 | 695 |
| new cases | South Asia | Both | 1990 | 619440 | 940460 | 394162 |
| new cases | South Asia | Both | 2021 | 2089040 | 3092356 | 1347962 |
| new cases | South Asia | Female | 1990 | 421581 | 652612 | 259375 |
| new cases | South Asia | Female | 2021 | 1521839 | 2279030 | 966433 |
| new cases | South Asia | Male | 1990 | 197859 | 287759 | 132299 |
| new cases | South Asia | Male | 2021 | 567200 | 823191 | 380477 |
| new cases | Southeast Asia | Both | 1990 | 158931 | 222206 | 110730 |
| new cases | Southeast Asia | Both | 2021 | 505512 | 702845 | 354723 |
| new cases | Southeast Asia | Female | 1990 | 108415 | 154504 | 73702 |
| new cases | Southeast Asia | Female | 2021 | 346154 | 486865 | 238665 |
| new cases | Southeast Asia | Male | 1990 | 50517 | 68144 | 36442 |
| new cases | Southeast Asia | Male | 2021 | 159358 | 217213 | 114850 |
| new cases | Southern Latin America | Both | 1990 | 50158 | 67365 | 36266 |
| new cases | Southern Latin America | Both | 2021 | 124482 | 164133 | 91117 |
| new cases | Southern Latin America | Female | 1990 | 36105 | 49028 | 25720 |
| new cases | Southern Latin America | Female | 2021 | 93907 | 125068 | 67830 |
| new cases | Southern Latin America | Male | 1990 | 14053 | 18457 | 10359 |
| new cases | Southern Latin America | Male | 2021 | 30575 | 40156 | 22494 |
| new cases | Southern Sub-Saharan Africa | Both | 1990 | 9895 | 13468 | 7101 |
| new cases | Southern Sub-Saharan Africa | Both | 2021 | 18921 | 25502 | 13721 |
| new cases | Southern Sub-Saharan Africa | Female | 1990 | 5684 | 7825 | 4023 |
| new cases | Southern Sub-Saharan Africa | Female | 2021 | 11082 | 15070 | 7987 |
| new cases | Southern Sub-Saharan Africa | Male | 1990 | 4211 | 5665 | 3046 |
| new cases | Southern Sub-Saharan Africa | Male | 2021 | 7839 | 10485 | 5701 |
| new cases | Tropical Latin America | Both | 1990 | 76705 | 110497 | 51670 |
| new cases | Tropical Latin America | Both | 2021 | 264880 | 379265 | 179134 |
| new cases | Tropical Latin America | Female | 1990 | 45492 | 67057 | 29878 |
| new cases | Tropical Latin America | Female | 2021 | 172772 | 251169 | 114515 |
| new cases | Tropical Latin America | Male | 1990 | 31213 | 43836 | 21622 |
| new cases | Tropical Latin America | Male | 2021 | 92107 | 128870 | 63663 |
| new cases | Western Europe | Both | 1990 | 1892099 | 2573328 | 1330211 |
| new cases | Western Europe | Both | 2021 | 3597979 | 4758674 | 2608372 |
| new cases | Western Europe | Female | 1990 | 1525841 | 2081171 | 1066670 |
| new cases | Western Europe | Female | 2021 | 2698713 | 3560140 | 1947386 |
| new cases | Western Europe | Male | 1990 | 366258 | 498295 | 260191 |
| new cases | Western Europe | Male | 2021 | 899267 | 1200998 | 651916 |
| new cases | Western Sub-Saharan Africa | Both | 1990 | 43411 | 59601 | 30947 |
| new cases | Western Sub-Saharan Africa | Both | 2021 | 104543 | 142549 | 74935 |
| new cases | Western Sub-Saharan Africa | Female | 1990 | 26345 | 36903 | 18391 |
| new cases | Western Sub-Saharan Africa | Female | 2021 | 65508 | 91120 | 46062 |
| new cases | Western Sub-Saharan Africa | Male | 1990 | 17066 | 22811 | 12518 |
| new cases | Western Sub-Saharan Africa | Male | 2021 | 39035 | 52020 | 28789 |
| prevalence count | Andean Latin America | Both | 1990 | 15846 | 18299 | 13823 |
| prevalence count | Andean Latin America | Both | 2021 | 61800 | 73261 | 52463 |
| prevalence count | Andean Latin America | Female | 1990 | 8477 | 9930 | 7286 |
| prevalence count | Andean Latin America | Female | 2021 | 35177 | 42073 | 29482 |
| prevalence count | Andean Latin America | Male | 1990 | 7369 | 8370 | 6497 |
| prevalence count | Andean Latin America | Male | 2021 | 26623 | 31422 | 22868 |
| prevalence count | Australasia | Both | 1990 | 137797 | 166824 | 113356 |
| prevalence count | Australasia | Both | 2021 | 530064 | 628390 | 444510 |
| prevalence count | Australasia | Female | 1990 | 104482 | 126633 | 85628 |
| prevalence count | Australasia | Female | 2021 | 364817 | 431292 | 305464 |
| prevalence count | Australasia | Male | 1990 | 33315 | 40398 | 27397 |
| prevalence count | Australasia | Male | 2021 | 165246 | 198272 | 137162 |
| prevalence count | Caribbean | Both | 1990 | 36994 | 44822 | 30678 |
| prevalence count | Caribbean | Both | 2021 | 135440 | 163317 | 111190 |
| prevalence count | Caribbean | Female | 1990 | 26270 | 32331 | 21430 |
| prevalence count | Caribbean | Female | 2021 | 101927 | 123471 | 82979 |
| prevalence count | Caribbean | Male | 1990 | 10724 | 12586 | 9167 |
| prevalence count | Caribbean | Male | 2021 | 33513 | 40173 | 27994 |
| prevalence count | Central Asia | Both | 1990 | 34116 | 39672 | 29442 |
| prevalence count | Central Asia | Both | 2021 | 56366 | 65602 | 48508 |
| prevalence count | Central Asia | Female | 1990 | 21771 | 25734 | 18458 |
| prevalence count | Central Asia | Female | 2021 | 34475 | 40751 | 29168 |
| prevalence count | Central Asia | Male | 1990 | 12345 | 14034 | 10909 |
| prevalence count | Central Asia | Male | 2021 | 21890 | 24997 | 19108 |
| prevalence count | Central Europe | Both | 1990 | 469058 | 579002 | 380616 |
| prevalence count | Central Europe | Both | 2021 | 700051 | 857514 | 571661 |
| prevalence count | Central Europe | Female | 1990 | 349731 | 436268 | 280415 |
| prevalence count | Central Europe | Female | 2021 | 486868 | 597515 | 396617 |
| prevalence count | Central Europe | Male | 1990 | 119328 | 143721 | 99470 |
| prevalence count | Central Europe | Male | 2021 | 213184 | 261562 | 173650 |
| prevalence count | Central Latin America | Both | 1990 | 135383 | 165652 | 112092 |
| prevalence count | Central Latin America | Both | 2021 | 369393 | 448653 | 305995 |
| prevalence count | Central Latin America | Female | 1990 | 81320 | 101761 | 65570 |
| prevalence count | Central Latin America | Female | 2021 | 232992 | 286145 | 189916 |
| prevalence count | Central Latin America | Male | 1990 | 54063 | 64138 | 46218 |
| prevalence count | Central Latin America | Male | 2021 | 136401 | 163453 | 115199 |
| prevalence count | Central Sub-Saharan Africa | Both | 1990 | 10572 | 12625 | 8934 |
| prevalence count | Central Sub-Saharan Africa | Both | 2021 | 32809 | 39830 | 27259 |
| prevalence count | Central Sub-Saharan Africa | Female | 1990 | 6573 | 7930 | 5497 |
| prevalence count | Central Sub-Saharan Africa | Female | 2021 | 21356 | 25906 | 17597 |
| prevalence count | Central Sub-Saharan Africa | Male | 1990 | 3999 | 4737 | 3406 |
| prevalence count | Central Sub-Saharan Africa | Male | 2021 | 11453 | 14024 | 9598 |
| prevalence count | East Asia | Both | 1990 | 873591 | 1091211 | 711404 |
| prevalence count | East Asia | Both | 2021 | 4301831 | 5400795 | 3441844 |
| prevalence count | East Asia | Female | 1990 | 572189 | 725052 | 460098 |
| prevalence count | East Asia | Female | 2021 | 2924451 | 3683396 | 2319869 |
| prevalence count | East Asia | Male | 1990 | 301402 | 367264 | 250544 |
| prevalence count | East Asia | Male | 2021 | 1377381 | 1715877 | 1119912 |
| prevalence count | Eastern Europe | Both | 1990 | 446507 | 537028 | 377486 |
| prevalence count | Eastern Europe | Both | 2021 | 595166 | 725586 | 493713 |
| prevalence count | Eastern Europe | Female | 1990 | 307186 | 372781 | 257589 |
| prevalence count | Eastern Europe | Female | 2021 | 389488 | 476033 | 321733 |
| prevalence count | Eastern Europe | Male | 1990 | 139321 | 163834 | 119550 |
| prevalence count | Eastern Europe | Male | 2021 | 205678 | 249342 | 171373 |
| prevalence count | Eastern Sub-Saharan Africa | Both | 1990 | 39204 | 47833 | 32451 |
| prevalence count | Eastern Sub-Saharan Africa | Both | 2021 | 112922 | 140695 | 92552 |
| prevalence count | Eastern Sub-Saharan Africa | Female | 1990 | 23601 | 29005 | 19438 |
| prevalence count | Eastern Sub-Saharan Africa | Female | 2021 | 72029 | 89741 | 58599 |
| prevalence count | Eastern Sub-Saharan Africa | Male | 1990 | 15603 | 18808 | 12933 |
| prevalence count | Eastern Sub-Saharan Africa | Male | 2021 | 40893 | 50881 | 33664 |
| prevalence count | Global | Both | 1990 | 10307585 | 12673120 | 8429708 |
| prevalence count | Global | Both | 2021 | 28145529 | 34274013 | 23040888 |
| prevalence count | Global | Female | 1990 | 7613231 | 9404134 | 6193147 |
| prevalence count | Global | Female | 2021 | 20012856 | 24404765 | 16363891 |
| prevalence count | Global | Male | 1990 | 2694354 | 3278060 | 2235706 |
| prevalence count | Global | Male | 2021 | 8132673 | 9868510 | 6701916 |
| prevalence count | High SDI | Both | 1990 | 5954124 | 7312688 | 4873277 |
| prevalence count | High SDI | Both | 2021 | 15101660 | 18209992 | 12451449 |
| prevalence count | High SDI | Female | 1990 | 4580329 | 5619337 | 3749145 |
| prevalence count | High SDI | Female | 2021 | 10844268 | 13038264 | 8953021 |
| prevalence count | High SDI | Male | 1990 | 1373795 | 1690928 | 1123306 |
| prevalence count | High SDI | Male | 2021 | 4257392 | 5175728 | 3477149 |
| prevalence count | High-income Asia Pacific | Both | 1990 | 750623 | 923661 | 610741 |
| prevalence count | High-income Asia Pacific | Both | 2021 | 2425740 | 2912698 | 2006254 |
| prevalence count | High-income Asia Pacific | Female | 1990 | 488152 | 599875 | 397536 |
| prevalence count | High-income Asia Pacific | Female | 2021 | 1591070 | 1897502 | 1322742 |
| prevalence count | High-income Asia Pacific | Male | 1990 | 262471 | 325272 | 213632 |
| prevalence count | High-income Asia Pacific | Male | 2021 | 834670 | 1016032 | 680602 |
| prevalence count | High-income North America | Both | 1990 | 1789004 | 2245457 | 1426972 |
| prevalence count | High-income North America | Both | 2021 | 5338784 | 6616887 | 4278919 |
| prevalence count | High-income North America | Female | 1990 | 1343338 | 1686112 | 1069114 |
| prevalence count | High-income North America | Female | 2021 | 3799813 | 4711934 | 3046331 |
| prevalence count | High-income North America | Male | 1990 | 445667 | 561970 | 356893 |
| prevalence count | High-income North America | Male | 2021 | 1538971 | 1911305 | 1229498 |
| prevalence count | High-middle SDI | Both | 1990 | 2383832 | 2926855 | 1955579 |
| prevalence count | High-middle SDI | Both | 2021 | 5581743 | 6788679 | 4589324 |
| prevalence count | High-middle SDI | Female | 1990 | 1721174 | 2131920 | 1399787 |
| prevalence count | High-middle SDI | Female | 2021 | 3946432 | 4806118 | 3234088 |
| prevalence count | High-middle SDI | Male | 1990 | 662659 | 798641 | 555406 |
| prevalence count | High-middle SDI | Male | 2021 | 1635311 | 1983386 | 1354524 |
| prevalence count | Low SDI | Both | 1990 | 169750 | 214023 | 136289 |
| prevalence count | Low SDI | Both | 2021 | 541354 | 685497 | 431403 |
| prevalence count | Low SDI | Female | 1990 | 109754 | 140868 | 86284 |
| prevalence count | Low SDI | Female | 2021 | 372486 | 477353 | 293248 |
| prevalence count | Low SDI | Male | 1990 | 59996 | 73639 | 49417 |
| prevalence count | Low SDI | Male | 2021 | 168868 | 210391 | 137782 |
| prevalence count | Low-middle SDI | Both | 1990 | 672252 | 864118 | 531476 |
| prevalence count | Low-middle SDI | Both | 2021 | 2210088 | 2806313 | 1751808 |
| prevalence count | Low-middle SDI | Female | 1990 | 459063 | 602120 | 354812 |
| prevalence count | Low-middle SDI | Female | 2021 | 1602022 | 2062198 | 1249039 |
| prevalence count | Low-middle SDI | Male | 1990 | 213189 | 262540 | 175587 |
| prevalence count | Low-middle SDI | Male | 2021 | 608066 | 748793 | 499233 |
| prevalence count | Middle SDI | Both | 1990 | 1114898 | 1386180 | 914202 |
| prevalence count | Middle SDI | Both | 2021 | 4686671 | 5870815 | 3768010 |
| prevalence count | Middle SDI | Female | 1990 | 733316 | 929424 | 588544 |
| prevalence count | Middle SDI | Female | 2021 | 3230304 | 4089250 | 2562325 |
| prevalence count | Middle SDI | Male | 1990 | 381582 | 457260 | 322431 |
| prevalence count | Middle SDI | Male | 2021 | 1456367 | 1789403 | 1195518 |
| prevalence count | North Africa and Middle East | Both | 1990 | 135222 | 157890 | 117503 |
| prevalence count | North Africa and Middle East | Both | 2021 | 499258 | 613453 | 416635 |
| prevalence count | North Africa and Middle East | Female | 1990 | 72770 | 86410 | 62199 |
| prevalence count | North Africa and Middle East | Female | 2021 | 286862 | 348274 | 238041 |
| prevalence count | North Africa and Middle East | Male | 1990 | 62452 | 71775 | 54930 |
| prevalence count | North Africa and Middle East | Male | 2021 | 212396 | 267092 | 176882 |
| prevalence count | Oceania | Both | 1990 | 1822 | 2218 | 1499 |
| prevalence count | Oceania | Both | 2021 | 7174 | 8734 | 5906 |
| prevalence count | Oceania | Female | 1990 | 1386 | 1745 | 1102 |
| prevalence count | Oceania | Female | 2021 | 5806 | 7221 | 4647 |
| prevalence count | Oceania | Male | 1990 | 435 | 492 | 384 |
| prevalence count | Oceania | Male | 2021 | 1368 | 1543 | 1214 |
| prevalence count | South Asia | Both | 1990 | 779783 | 1028078 | 596604 |
| prevalence count | South Asia | Both | 2021 | 3121752 | 4052709 | 2418553 |
| prevalence count | South Asia | Female | 1990 | 555537 | 744212 | 417418 |
| prevalence count | South Asia | Female | 2021 | 2357729 | 3089627 | 1808188 |
| prevalence count | South Asia | Male | 1990 | 224245 | 285443 | 178058 |
| prevalence count | South Asia | Male | 2021 | 764023 | 964660 | 609393 |
| prevalence count | Southeast Asia | Both | 1990 | 241524 | 297211 | 199029 |
| prevalence count | Southeast Asia | Both | 2021 | 817900 | 1012267 | 665901 |
| prevalence count | Southeast Asia | Female | 1990 | 169584 | 213066 | 137137 |
| prevalence count | Southeast Asia | Female | 2021 | 572807 | 712671 | 462366 |
| prevalence count | Southeast Asia | Male | 1990 | 71940 | 85045 | 61354 |
| prevalence count | Southeast Asia | Male | 2021 | 245093 | 303454 | 202077 |
| prevalence count | Southern Latin America | Both | 1990 | 120900 | 144468 | 100959 |
| prevalence count | Southern Latin America | Both | 2021 | 301385 | 357857 | 252376 |
| prevalence count | Southern Latin America | Female | 1990 | 86063 | 104274 | 70687 |
| prevalence count | Southern Latin America | Female | 2021 | 221555 | 264744 | 183651 |
| prevalence count | Southern Latin America | Male | 1990 | 34837 | 40542 | 29955 |
| prevalence count | Southern Latin America | Male | 2021 | 79830 | 93707 | 68061 |
| prevalence count | Southern Sub-Saharan Africa | Both | 1990 | 18272 | 21016 | 15929 |
| prevalence count | Southern Sub-Saharan Africa | Both | 2021 | 26602 | 31239 | 22713 |
| prevalence count | Southern Sub-Saharan Africa | Female | 1990 | 11130 | 12974 | 9618 |
| prevalence count | Southern Sub-Saharan Africa | Female | 2021 | 16354 | 19476 | 13825 |
| prevalence count | Southern Sub-Saharan Africa | Male | 1990 | 7142 | 8070 | 6294 |
| prevalence count | Southern Sub-Saharan Africa | Male | 2021 | 10247 | 11846 | 8845 |
| prevalence count | Tropical Latin America | Both | 1990 | 130234 | 161179 | 106975 |
| prevalence count | Tropical Latin America | Both | 2021 | 490767 | 605678 | 400289 |
| prevalence count | Tropical Latin America | Female | 1990 | 79822 | 100820 | 64289 |
| prevalence count | Tropical Latin America | Female | 2021 | 331032 | 413458 | 265392 |
| prevalence count | Tropical Latin America | Male | 1990 | 50412 | 60688 | 42491 |
| prevalence count | Tropical Latin America | Male | 2021 | 159735 | 192525 | 134048 |
| prevalence count | Western Europe | Both | 1990 | 4086381 | 4978814 | 3357415 |
| prevalence count | Western Europe | Both | 2021 | 8076315 | 9585736 | 6758264 |
| prevalence count | Western Europe | Female | 1990 | 3268711 | 3992394 | 2684638 |
| prevalence count | Western Europe | Female | 2021 | 6071376 | 7187935 | 5081512 |
| prevalence count | Western Europe | Male | 1990 | 817669 | 994558 | 672810 |
| prevalence count | Western Europe | Male | 2021 | 2004939 | 2407382 | 1663775 |
| prevalence count | Western Sub-Saharan Africa | Both | 1990 | 54754 | 67573 | 44823 |
| prevalence count | Western Sub-Saharan Africa | Both | 2021 | 144012 | 176638 | 118023 |
| prevalence count | Western Sub-Saharan Africa | Female | 1990 | 35138 | 44217 | 28355 |
| prevalence count | Western Sub-Saharan Africa | Female | 2021 | 94874 | 117954 | 76594 |
| prevalence count | Western Sub-Saharan Africa | Male | 1990 | 19616 | 23512 | 16432 |
| prevalence count | Western Sub-Saharan Africa | Male | 2021 | 49138 | 58948 | 41203 |
| YLDs | Andean Latin America | Both | 1990 | 3586 | 4850 | 2446 |
| YLDs | Andean Latin America | Both | 2021 | 8632 | 11788 | 5981 |
| YLDs | Andean Latin America | Female | 1990 | 1902 | 2626 | 1282 |
| YLDs | Andean Latin America | Female | 2021 | 4870 | 6700 | 3318 |
| YLDs | Andean Latin America | Male | 1990 | 1685 | 2328 | 1135 |
| YLDs | Andean Latin America | Male | 2021 | 3762 | 5189 | 2578 |
| YLDs | Australasia | Both | 1990 | 9847 | 14036 | 6551 |
| YLDs | Australasia | Both | 2021 | 37839 | 53798 | 25378 |
| YLDs | Australasia | Female | 1990 | 7429 | 10619 | 4940 |
| YLDs | Australasia | Female | 2021 | 25888 | 36809 | 17307 |
| YLDs | Australasia | Male | 1990 | 2418 | 3418 | 1595 |
| YLDs | Australasia | Male | 2021 | 11951 | 16972 | 7973 |
| YLDs | Caribbean | Both | 1990 | 5958 | 8188 | 4036 |
| YLDs | Caribbean | Both | 2021 | 15077 | 21085 | 10110 |
| YLDs | Caribbean | Female | 1990 | 4129 | 5720 | 2762 |
| YLDs | Caribbean | Female | 2021 | 11008 | 15553 | 7317 |
| YLDs | Caribbean | Male | 1990 | 1829 | 2511 | 1244 |
| YLDs | Caribbean | Male | 2021 | 4070 | 5671 | 2740 |
| YLDs | Central Asia | Both | 1990 | 6234 | 8356 | 4324 |
| YLDs | Central Asia | Both | 2021 | 8114 | 10960 | 5631 |
| YLDs | Central Asia | Female | 1990 | 3917 | 5299 | 2706 |
| YLDs | Central Asia | Female | 2021 | 4825 | 6516 | 3296 |
| YLDs | Central Asia | Male | 1990 | 2317 | 3116 | 1593 |
| YLDs | Central Asia | Male | 2021 | 3289 | 4492 | 2263 |
| YLDs | Central Europe | Both | 1990 | 66442 | 91880 | 44878 |
| YLDs | Central Europe | Both | 2021 | 56442 | 81070 | 37034 |
| YLDs | Central Europe | Female | 1990 | 48580 | 67410 | 32771 |
| YLDs | Central Europe | Female | 2021 | 38268 | 54995 | 25070 |
| YLDs | Central Europe | Male | 1990 | 17862 | 24619 | 12150 |
| YLDs | Central Europe | Male | 2021 | 18174 | 26274 | 11920 |
| YLDs | Central Latin America | Both | 1990 | 27261 | 37653 | 18621 |
| YLDs | Central Latin America | Both | 2021 | 51590 | 70922 | 35506 |
| YLDs | Central Latin America | Female | 1990 | 16190 | 22563 | 10985 |
| YLDs | Central Latin America | Female | 2021 | 32078 | 44191 | 21855 |
| YLDs | Central Latin America | Male | 1990 | 11071 | 15068 | 7667 |
| YLDs | Central Latin America | Male | 2021 | 19512 | 26740 | 13435 |
| YLDs | Central Sub-Saharan Africa | Both | 1990 | 2820 | 3969 | 1895 |
| YLDs | Central Sub-Saharan Africa | Both | 2021 | 7484 | 10425 | 5064 |
| YLDs | Central Sub-Saharan Africa | Female | 1990 | 1743 | 2476 | 1145 |
| YLDs | Central Sub-Saharan Africa | Female | 2021 | 4838 | 6820 | 3190 |
| YLDs | Central Sub-Saharan Africa | Male | 1990 | 1077 | 1535 | 703 |
| YLDs | Central Sub-Saharan Africa | Male | 2021 | 2646 | 3805 | 1730 |
| YLDs | East Asia | Both | 1990 | 169160 | 236362 | 114498 |
| YLDs | East Asia | Both | 2021 | 367024 | 530793 | 239022 |
| YLDs | East Asia | Female | 1990 | 109482 | 153291 | 73792 |
| YLDs | East Asia | Female | 2021 | 243413 | 351408 | 158587 |
| YLDs | East Asia | Male | 1990 | 59678 | 83538 | 40667 |
| YLDs | East Asia | Male | 2021 | 123611 | 180027 | 80575 |
| YLDs | Eastern Europe | Both | 1990 | 62924 | 85081 | 43599 |
| YLDs | Eastern Europe | Both | 2021 | 54505 | 76207 | 36807 |
| YLDs | Eastern Europe | Female | 1990 | 42715 | 58372 | 29658 |
| YLDs | Eastern Europe | Female | 2021 | 34490 | 48272 | 23373 |
| YLDs | Eastern Europe | Male | 1990 | 20209 | 27234 | 14039 |
| YLDs | Eastern Europe | Male | 2021 | 20016 | 28135 | 13394 |
| YLDs | Eastern Sub-Saharan Africa | Both | 1990 | 10244 | 14241 | 6933 |
| YLDs | Eastern Sub-Saharan Africa | Both | 2021 | 24777 | 34451 | 16639 |
| YLDs | Eastern Sub-Saharan Africa | Female | 1990 | 6107 | 8532 | 4129 |
| YLDs | Eastern Sub-Saharan Africa | Female | 2021 | 15597 | 21671 | 10496 |
| YLDs | Eastern Sub-Saharan Africa | Male | 1990 | 4137 | 5820 | 2798 |
| YLDs | Eastern Sub-Saharan Africa | Male | 2021 | 9181 | 12872 | 6098 |
| YLDs | Global | Both | 1990 | 1218576 | 1687207 | 834435 |
| YLDs | Global | Both | 2021 | 2578437 | 3605325 | 1747997 |
| YLDs | Global | Female | 1990 | 868561 | 1209340 | 593585 |
| YLDs | Global | Female | 2021 | 1813157 | 2537139 | 1224875 |
| YLDs | Global | Male | 1990 | 350015 | 479741 | 241494 |
| YLDs | Global | Male | 2021 | 765280 | 1069245 | 520122 |
| YLDs | High SDI | Both | 1990 | 492925 | 689494 | 333552 |
| YLDs | High SDI | Both | 2021 | 1039811 | 1483692 | 688489 |
| YLDs | High SDI | Female | 1990 | 377433 | 528248 | 255587 |
| YLDs | High SDI | Female | 2021 | 741080 | 1056576 | 490703 |
| YLDs | High SDI | Male | 1990 | 115492 | 161905 | 77719 |
| YLDs | High SDI | Male | 2021 | 298730 | 427794 | 197006 |
| YLDs | High-income Asia Pacific | Both | 1990 | 60774 | 84768 | 41228 |
| YLDs | High-income Asia Pacific | Both | 2021 | 162804 | 231850 | 106827 |
| YLDs | High-income Asia Pacific | Female | 1990 | 39535 | 55388 | 26899 |
| YLDs | High-income Asia Pacific | Female | 2021 | 106359 | 151269 | 70032 |
| YLDs | High-income Asia Pacific | Male | 1990 | 21239 | 29678 | 14363 |
| YLDs | High-income Asia Pacific | Male | 2021 | 56445 | 80953 | 36854 |
| YLDs | High-income North America | Both | 1990 | 128835 | 185937 | 84618 |
| YLDs | High-income North America | Both | 2021 | 356407 | 511706 | 230495 |
| YLDs | High-income North America | Female | 1990 | 95938 | 138808 | 62911 |
| YLDs | High-income North America | Female | 2021 | 251040 | 360323 | 162141 |
| YLDs | High-income North America | Male | 1990 | 32896 | 47010 | 21550 |
| YLDs | High-income North America | Male | 2021 | 105367 | 151258 | 68592 |
| YLDs | High-middle SDI | Both | 1990 | 285520 | 393499 | 196002 |
| YLDs | High-middle SDI | Both | 2021 | 442506 | 627776 | 295258 |
| YLDs | High-middle SDI | Female | 1990 | 199885 | 277011 | 137068 |
| YLDs | High-middle SDI | Female | 2021 | 306628 | 435294 | 204325 |
| YLDs | High-middle SDI | Male | 1990 | 85635 | 116430 | 59178 |
| YLDs | High-middle SDI | Male | 2021 | 135878 | 193589 | 90496 |
| YLDs | Low SDI | Both | 1990 | 43857 | 61749 | 29273 |
| YLDs | Low SDI | Both | 2021 | 112729 | 157964 | 75211 |
| YLDs | Low SDI | Female | 1990 | 28080 | 39801 | 18551 |
| YLDs | Low SDI | Female | 2021 | 76456 | 107602 | 50859 |
| YLDs | Low SDI | Male | 1990 | 15778 | 22111 | 10642 |
| YLDs | Low SDI | Male | 2021 | 36273 | 50963 | 24479 |
| YLDs | Low-middle SDI | Both | 1990 | 161624 | 230028 | 106771 |
| YLDs | Low-middle SDI | Both | 2021 | 405923 | 568845 | 271058 |
| YLDs | Low-middle SDI | Female | 1990 | 109714 | 158062 | 71592 |
| YLDs | Low-middle SDI | Female | 2021 | 292336 | 412894 | 193535 |
| YLDs | Low-middle SDI | Male | 1990 | 51910 | 72889 | 34958 |
| YLDs | Low-middle SDI | Male | 2021 | 113587 | 158300 | 76613 |
| YLDs | Middle SDI | Both | 1990 | 232982 | 324321 | 157362 |
| YLDs | Middle SDI | Both | 2021 | 575315 | 807799 | 388326 |
| YLDs | Middle SDI | Female | 1990 | 152225 | 212903 | 102345 |
| YLDs | Middle SDI | Female | 2021 | 395131 | 555324 | 265176 |
| YLDs | Middle SDI | Male | 1990 | 80757 | 111736 | 55162 |
| YLDs | Middle SDI | Male | 2021 | 180184 | 252095 | 122641 |
| YLDs | North Africa and Middle East | Both | 1990 | 27820 | 37336 | 19138 |
| YLDs | North Africa and Middle East | Both | 2021 | 59593 | 82074 | 40878 |
| YLDs | North Africa and Middle East | Female | 1990 | 14775 | 20138 | 10108 |
| YLDs | North Africa and Middle East | Female | 2021 | 33614 | 46157 | 22866 |
| YLDs | North Africa and Middle East | Male | 1990 | 13045 | 17614 | 9011 |
| YLDs | North Africa and Middle East | Male | 2021 | 25979 | 36236 | 17863 |
| YLDs | Oceania | Both | 1990 | 427 | 603 | 283 |
| YLDs | Oceania | Both | 2021 | 1542 | 2153 | 1030 |
| YLDs | Oceania | Female | 1990 | 324 | 468 | 212 |
| YLDs | Oceania | Female | 2021 | 1246 | 1764 | 816 |
| YLDs | Oceania | Male | 1990 | 102 | 146 | 67 |
| YLDs | Oceania | Male | 2021 | 295 | 419 | 193 |
| YLDs | South Asia | Both | 1990 | 187500 | 270936 | 122162 |
| YLDs | South Asia | Both | 2021 | 547883 | 774163 | 361589 |
| YLDs | South Asia | Female | 1990 | 132370 | 193283 | 85408 |
| YLDs | South Asia | Female | 2021 | 409448 | 581146 | 268965 |
| YLDs | South Asia | Male | 1990 | 55130 | 78798 | 36547 |
| YLDs | South Asia | Male | 2021 | 138436 | 194787 | 92615 |
| YLDs | Southeast Asia | Both | 1990 | 52547 | 72928 | 35502 |
| YLDs | Southeast Asia | Both | 2021 | 124305 | 172708 | 83901 |
| YLDs | Southeast Asia | Female | 1990 | 36690 | 51389 | 24618 |
| YLDs | Southeast Asia | Female | 2021 | 86081 | 119691 | 57861 |
| YLDs | Southeast Asia | Male | 1990 | 15857 | 21729 | 10753 |
| YLDs | Southeast Asia | Male | 2021 | 38224 | 53689 | 25987 |
| YLDs | Southern Latin America | Both | 1990 | 20227 | 27657 | 13899 |
| YLDs | Southern Latin America | Both | 2021 | 29900 | 40893 | 20536 |
| YLDs | Southern Latin America | Female | 1990 | 14344 | 19763 | 9808 |
| YLDs | Southern Latin America | Female | 2021 | 22030 | 30262 | 15000 |
| YLDs | Southern Latin America | Male | 1990 | 5884 | 8028 | 4037 |
| YLDs | Southern Latin America | Male | 2021 | 7870 | 10704 | 5441 |
| YLDs | Southern Sub-Saharan Africa | Both | 1990 | 3871 | 5202 | 2663 |
| YLDs | Southern Sub-Saharan Africa | Both | 2021 | 4901 | 6658 | 3359 |
| YLDs | Southern Sub-Saharan Africa | Female | 1990 | 2333 | 3156 | 1599 |
| YLDs | Southern Sub-Saharan Africa | Female | 2021 | 2991 | 4084 | 2026 |
| YLDs | Southern Sub-Saharan Africa | Male | 1990 | 1539 | 2080 | 1047 |
| YLDs | Southern Sub-Saharan Africa | Male | 2021 | 1911 | 2596 | 1309 |
| YLDs | Tropical Latin America | Both | 1990 | 26229 | 36377 | 17917 |
| YLDs | Tropical Latin America | Both | 2021 | 69066 | 95456 | 47351 |
| YLDs | Tropical Latin America | Female | 1990 | 15910 | 22301 | 10783 |
| YLDs | Tropical Latin America | Female | 2021 | 46066 | 64137 | 31307 |
| YLDs | Tropical Latin America | Male | 1990 | 10319 | 14106 | 7093 |
| YLDs | Tropical Latin America | Male | 2021 | 23001 | 31429 | 15813 |
| YLDs | Western Europe | Both | 1990 | 332512 | 465277 | 224695 |
| YLDs | Western Europe | Both | 2021 | 560823 | 796929 | 374931 |
| YLDs | Western Europe | Female | 1990 | 265695 | 372488 | 179394 |
| YLDs | Western Europe | Female | 2021 | 419778 | 596132 | 280296 |
| YLDs | Western Europe | Male | 1990 | 66817 | 93492 | 44927 |
| YLDs | Western Europe | Male | 2021 | 141045 | 201906 | 93969 |
| YLDs | Western Sub-Saharan Africa | Both | 1990 | 13357 | 18646 | 8982 |
| YLDs | Western Sub-Saharan Africa | Both | 2021 | 29729 | 41135 | 20044 |
| YLDs | Western Sub-Saharan Africa | Female | 1990 | 8453 | 11887 | 5666 |
| YLDs | Western Sub-Saharan Africa | Female | 2021 | 19232 | 26774 | 12908 |
| YLDs | Western Sub-Saharan Africa | Male | 1990 | 4904 | 6768 | 3305 |
| YLDs | Western Sub-Saharan Africa | Male | 2021 | 10498 | 14506 | 7082 |

Abbreviations: YLDs, years lived with disability; HFs, Hip fractures; SDI, sociodemographic index; GBD, Global Burden of Disease Study; UI, uncertainty interval.

**Table S3. The results of ASIR, ASPR, and ASYR for HFs (≥55 years) across 204 countries and territories**

| **measure** | **location** | **sex** | **year** | **value** | **95% UI_upper** | **95% UI_lower** |
| --- | --- | --- | --- | --- | --- | --- |
| ASIR | China | Both | 2021 | 844.93 | 1232.02 | 557.46 |
| ASIR | Taiwan (Province of China) | Both | 2021 | 372.63 | 503.17 | 269.36 |
| ASIR | Cambodia | Both | 2021 | 769.12 | 1066.23 | 536.06 |
| ASIR | Democratic People's Republic of Korea | Both | 2021 | 225.34 | 296.81 | 167.45 |
| ASIR | Malaysia | Both | 2021 | 479.23 | 651.18 | 342.86 |
| ASIR | Indonesia | Both | 2021 | 547.05 | 790.99 | 365.63 |
| ASIR | Lao People's Democratic Republic | Both | 2021 | 339.37 | 458.73 | 246.54 |
| ASIR | Maldives | Both | 2021 | 498.75 | 686.23 | 347.57 |
| ASIR | Myanmar | Both | 2021 | 559.63 | 766.40 | 397.83 |
| ASIR | Thailand | Both | 2021 | 394.89 | 541.55 | 281.68 |
| ASIR | Philippines | Both | 2021 | 339.28 | 480.15 | 231.91 |
| ASIR | Timor-Leste | Both | 2021 | 362.67 | 489.43 | 263.37 |
| ASIR | Sri Lanka | Both | 2021 | 798.83 | 1089.61 | 564.68 |
| ASIR | Viet Nam | Both | 2021 | 1039.00 | 1454.72 | 712.98 |
| ASIR | Kiribati | Both | 2021 | 184.65 | 246.97 | 133.75 |
| ASIR | Fiji | Both | 2021 | 271.10 | 362.74 | 198.57 |
| ASIR | Marshall Islands | Both | 2021 | 357.24 | 478.20 | 258.83 |
| ASIR | Micronesia (Federated States of) | Both | 2021 | 450.70 | 599.97 | 328.32 |
| ASIR | Solomon Islands | Both | 2021 | 858.13 | 1218.67 | 580.41 |
| ASIR | Papua New Guinea | Both | 2021 | 734.84 | 1039.35 | 498.18 |
| ASIR | Tonga | Both | 2021 | 320.28 | 430.82 | 230.53 |
| ASIR | Samoa | Both | 2021 | 421.20 | 566.08 | 301.94 |
| ASIR | Armenia | Both | 2021 | 309.68 | 415.46 | 228.75 |
| ASIR | Vanuatu | Both | 2021 | 292.67 | 393.96 | 212.77 |
| ASIR | Azerbaijan | Both | 2021 | 206.54 | 283.41 | 150.04 |
| ASIR | Kyrgyzstan | Both | 2021 | 224.18 | 296.78 | 166.59 |
| ASIR | Georgia | Both | 2021 | 448.96 | 610.11 | 324.92 |
| ASIR | Mongolia | Both | 2021 | 281.50 | 381.58 | 205.17 |
| ASIR | Kazakhstan | Both | 2021 | 370.11 | 502.59 | 266.98 |
| ASIR | Tajikistan | Both | 2021 | 199.92 | 271.17 | 146.84 |
| ASIR | Turkmenistan | Both | 2021 | 176.19 | 247.57 | 126.17 |
| ASIR | Albania | Both | 2021 | 488.63 | 710.10 | 338.81 |
| ASIR | Uzbekistan | Both | 2021 | 241.32 | 320.15 | 178.16 |
| ASIR | Czechia | Both | 2021 | 1535.11 | 2118.79 | 1075.25 |
| ASIR | Bosnia and Herzegovina | Both | 2021 | 613.98 | 857.97 | 433.03 |
| ASIR | Hungary | Both | 2021 | 1555.98 | 2120.32 | 1098.41 |
| ASIR | Bulgaria | Both | 2021 | 522.13 | 743.20 | 372.90 |
| ASIR | Croatia | Both | 2021 | 2511.16 | 3430.68 | 1746.66 |
| ASIR | North Macedonia | Both | 2021 | 864.58 | 1198.52 | 610.76 |
| ASIR | Romania | Both | 2021 | 559.57 | 777.93 | 399.94 |
| ASIR | Montenegro | Both | 2021 | 851.00 | 1169.06 | 602.79 |
| ASIR | Serbia | Both | 2021 | 775.80 | 1069.15 | 546.77 |
| ASIR | Poland | Both | 2021 | 1047.11 | 1523.13 | 689.85 |
| ASIR | Slovakia | Both | 2021 | 1338.34 | 1846.48 | 934.00 |
| ASIR | Belarus | Both | 2021 | 589.72 | 804.52 | 423.73 |
| ASIR | Slovenia | Both | 2021 | 2329.67 | 3196.13 | 1622.94 |
| ASIR | Estonia | Both | 2021 | 545.74 | 745.31 | 388.55 |
| ASIR | Latvia | Both | 2021 | 638.17 | 868.93 | 454.14 |
| ASIR | Russian Federation | Both | 2021 | 566.63 | 794.16 | 395.70 |
| ASIR | Lithuania | Both | 2021 | 803.64 | 1095.95 | 570.58 |
| ASIR | Ukraine | Both | 2021 | 401.50 | 562.23 | 279.53 |
| ASIR | Republic of Moldova | Both | 2021 | 378.31 | 509.09 | 277.18 |
| ASIR | Japan | Both | 2021 | 737.17 | 1062.59 | 487.01 |
| ASIR | Brunei Darussalam | Both | 2021 | 929.76 | 1229.20 | 679.93 |
| ASIR | Republic of Korea | Both | 2021 | 1725.89 | 2320.68 | 1233.55 |
| ASIR | Singapore | Both | 2021 | 609.01 | 813.15 | 435.80 |
| ASIR | New Zealand | Both | 2021 | 2169.13 | 3081.35 | 1445.32 |
| ASIR | Australia | Both | 2021 | 2506.20 | 3318.79 | 1801.36 |
| ASIR | Andorra | Both | 2021 | 3432.18 | 4652.60 | 2390.22 |
| ASIR | Cyprus | Both | 2021 | 2254.63 | 3006.19 | 1617.17 |
| ASIR | Denmark | Both | 2021 | 1790.21 | 2381.91 | 1295.42 |
| ASIR | Austria | Both | 2021 | 1927.01 | 2593.96 | 1367.61 |
| ASIR | Belgium | Both | 2021 | 2583.58 | 3478.75 | 1821.03 |
| ASIR | Finland | Both | 2021 | 2368.07 | 3187.17 | 1683.19 |
| ASIR | France | Both | 2021 | 2408.80 | 3237.89 | 1732.44 |
| ASIR | Greece | Both | 2021 | 649.61 | 870.59 | 465.00 |
| ASIR | Iceland | Both | 2021 | 1455.29 | 1934.71 | 1047.93 |
| ASIR | Ireland | Both | 2021 | 1312.58 | 1749.72 | 938.85 |
| ASIR | Luxembourg | Both | 2021 | 2092.95 | 2786.59 | 1484.75 |
| ASIR | Germany | Both | 2021 | 1866.39 | 2508.01 | 1336.12 |
| ASIR | Israel | Both | 2021 | 920.45 | 1214.88 | 664.05 |
| ASIR | Italy | Both | 2021 | 1589.31 | 2280.72 | 1042.80 |
| ASIR | Malta | Both | 2021 | 1365.38 | 1840.50 | 968.25 |
| ASIR | Spain | Both | 2021 | 1056.43 | 1425.41 | 753.13 |
| ASIR | Netherlands | Both | 2021 | 2810.69 | 3713.90 | 2011.12 |
| ASIR | Norway | Both | 2021 | 2877.33 | 4177.22 | 1871.62 |
| ASIR | Sweden | Both | 2021 | 2091.01 | 3040.08 | 1351.02 |
| ASIR | Portugal | Both | 2021 | 955.35 | 1279.30 | 682.42 |
| ASIR | Argentina | Both | 2021 | 589.32 | 782.69 | 429.10 |
| ASIR | Switzerland | Both | 2021 | 2728.77 | 3622.15 | 1946.43 |
| ASIR | Chile | Both | 2021 | 1235.58 | 1646.61 | 887.56 |
| ASIR | United Kingdom | Both | 2021 | 1478.13 | 2096.91 | 985.01 |
| ASIR | Canada | Both | 2021 | 2279.67 | 3056.34 | 1624.20 |
| ASIR | United States of America | Both | 2021 | 1628.51 | 2384.44 | 1046.74 |
| ASIR | Uruguay | Both | 2021 | 928.06 | 1236.83 | 669.26 |
| ASIR | Bahamas | Both | 2021 | 393.54 | 527.10 | 287.52 |
| ASIR | Antigua and Barbuda | Both | 2021 | 453.44 | 644.32 | 320.35 |
| ASIR | Dominican Republic | Both | 2021 | 369.59 | 494.95 | 272.37 |
| ASIR | Barbados | Both | 2021 | 394.23 | 530.91 | 285.48 |
| ASIR | Grenada | Both | 2021 | 468.01 | 642.97 | 338.73 |
| ASIR | Belize | Both | 2021 | 375.27 | 508.62 | 273.97 |
| ASIR | Cuba | Both | 2021 | 1671.13 | 2266.72 | 1173.73 |
| ASIR | Guyana | Both | 2021 | 530.85 | 712.48 | 388.06 |
| ASIR | Dominica | Both | 2021 | 282.79 | 382.13 | 209.09 |
| ASIR | Saint Lucia | Both | 2021 | 338.55 | 456.93 | 248.18 |
| ASIR | Saint Vincent and the Grenadines | Both | 2021 | 386.46 | 522.99 | 282.84 |
| ASIR | Haiti | Both | 2021 | 362.12 | 503.78 | 260.42 |
| ASIR | Suriname | Both | 2021 | 365.31 | 494.08 | 265.55 |
| ASIR | Jamaica | Both | 2021 | 289.69 | 392.95 | 209.76 |
| ASIR | Bolivia (Plurinational State of) | Both | 2021 | 439.63 | 593.30 | 321.97 |
| ASIR | Ecuador | Both | 2021 | 452.15 | 601.03 | 334.23 |
| ASIR | Trinidad and Tobago | Both | 2021 | 253.83 | 338.18 | 187.30 |
| ASIR | Peru | Both | 2021 | 311.81 | 425.16 | 227.50 |
| ASIR | Costa Rica | Both | 2021 | 771.07 | 1042.77 | 548.34 |
| ASIR | Colombia | Both | 2021 | 334.95 | 448.08 | 247.00 |
| ASIR | El Salvador | Both | 2021 | 567.62 | 759.07 | 414.21 |
| ASIR | Guatemala | Both | 2021 | 583.48 | 786.78 | 424.94 |
| ASIR | Nicaragua | Both | 2021 | 684.76 | 926.37 | 488.54 |
| ASIR | Honduras | Both | 2021 | 399.09 | 542.34 | 288.86 |
| ASIR | Panama | Both | 2021 | 312.40 | 420.18 | 228.11 |
| ASIR | Paraguay | Both | 2021 | 454.42 | 605.35 | 331.95 |
| ASIR | Venezuela (Bolivarian Republic of) | Both | 2021 | 452.24 | 604.77 | 330.12 |
| ASIR | Mexico | Both | 2021 | 639.74 | 912.42 | 435.20 |
| ASIR | Brazil | Both | 2021 | 639.80 | 917.05 | 431.42 |
| ASIR | Egypt | Both | 2021 | 292.32 | 391.21 | 214.80 |
| ASIR | Algeria | Both | 2021 | 372.00 | 495.70 | 271.88 |
| ASIR | Iran (Islamic Republic of) | Both | 2021 | 422.50 | 587.64 | 293.83 |
| ASIR | Iraq | Both | 2021 | 324.71 | 445.78 | 235.44 |
| ASIR | Lebanon | Both | 2021 | 353.47 | 481.07 | 253.39 |
| ASIR | Bahrain | Both | 2021 | 313.67 | 415.88 | 230.87 |
| ASIR | Jordan | Both | 2021 | 285.21 | 379.94 | 209.87 |
| ASIR | Libya | Both | 2021 | 952.93 | 1657.02 | 568.48 |
| ASIR | Kuwait | Both | 2021 | 297.48 | 389.95 | 221.13 |
| ASIR | Qatar | Both | 2021 | 578.25 | 773.92 | 416.82 |
| ASIR | Morocco | Both | 2021 | 542.83 | 751.39 | 383.34 |
| ASIR | Saudi Arabia | Both | 2021 | 1052.74 | 1438.97 | 740.66 |
| ASIR | Palestine | Both | 2021 | 409.73 | 555.30 | 291.81 |
| ASIR | Oman | Both | 2021 | 932.83 | 1259.22 | 670.91 |
| ASIR | Syrian Arab Republic | Both | 2021 | 329.50 | 436.43 | 242.92 |
| ASIR | United Arab Emirates | Both | 2021 | 314.79 | 421.87 | 231.71 |
| ASIR | Tunisia | Both | 2021 | 385.36 | 515.96 | 278.84 |
| ASIR | Yemen | Both | 2021 | 401.18 | 565.59 | 288.02 |
| ASIR | Turkey | Both | 2021 | 611.67 | 839.87 | 425.31 |
| ASIR | Afghanistan | Both | 2021 | 481.59 | 854.02 | 298.07 |
| ASIR | Bangladesh | Both | 2021 | 166.39 | 222.29 | 123.36 |
| ASIR | Bhutan | Both | 2021 | 897.72 | 1261.78 | 615.30 |
| ASIR | Pakistan | Both | 2021 | 251.22 | 344.02 | 179.14 |
| ASIR | India | Both | 2021 | 1219.96 | 1806.16 | 784.79 |
| ASIR | Nepal | Both | 2021 | 675.11 | 973.00 | 454.39 |
| ASIR | Angola | Both | 2021 | 361.33 | 487.48 | 262.75 |
| ASIR | Congo | Both | 2021 | 358.21 | 481.58 | 262.29 |
| ASIR | Democratic Republic of the Congo | Both | 2021 | 350.82 | 472.24 | 253.85 |
| ASIR | Central African Republic | Both | 2021 | 301.56 | 401.05 | 223.98 |
| ASIR | Equatorial Guinea | Both | 2021 | 405.58 | 548.53 | 293.16 |
| ASIR | Burundi | Both | 2021 | 330.19 | 438.83 | 243.41 |
| ASIR | Gabon | Both | 2021 | 443.29 | 597.81 | 319.36 |
| ASIR | Comoros | Both | 2021 | 359.70 | 478.27 | 267.34 |
| ASIR | Djibouti | Both | 2021 | 379.77 | 505.31 | 280.87 |
| ASIR | Kenya | Both | 2021 | 505.09 | 700.72 | 355.79 |
| ASIR | Eritrea | Both | 2021 | 406.14 | 544.63 | 298.23 |
| ASIR | Madagascar | Both | 2021 | 267.51 | 360.80 | 197.76 |
| ASIR | Ethiopia | Both | 2021 | 449.88 | 623.36 | 317.15 |
| ASIR | Malawi | Both | 2021 | 375.69 | 505.84 | 275.11 |
| ASIR | Rwanda | Both | 2021 | 385.48 | 512.47 | 281.69 |
| ASIR | Mauritius | Both | 2021 | 223.46 | 298.55 | 163.42 |
| ASIR | Seychelles | Both | 2021 | 276.51 | 370.17 | 202.14 |
| ASIR | Mozambique | Both | 2021 | 406.24 | 537.78 | 299.97 |
| ASIR | Somalia | Both | 2021 | 348.06 | 464.91 | 258.76 |
| ASIR | Zambia | Both | 2021 | 447.34 | 597.81 | 327.69 |
| ASIR | United Republic of Tanzania | Both | 2021 | 382.27 | 515.57 | 279.04 |
| ASIR | Uganda | Both | 2021 | 388.91 | 517.21 | 285.37 |
| ASIR | Botswana | Both | 2021 | 284.41 | 379.71 | 209.39 |
| ASIR | South Africa | Both | 2021 | 219.37 | 298.87 | 157.41 |
| ASIR | Lesotho | Both | 2021 | 277.12 | 364.69 | 205.72 |
| ASIR | Eswatini | Both | 2021 | 244.48 | 324.25 | 182.13 |
| ASIR | Namibia | Both | 2021 | 266.63 | 351.95 | 197.37 |
| ASIR | Zimbabwe | Both | 2021 | 339.56 | 453.79 | 249.51 |
| ASIR | Benin | Both | 2021 | 398.65 | 534.14 | 290.53 |
| ASIR | Burkina Faso | Both | 2021 | 465.68 | 616.99 | 343.07 |
| ASIR | Cameroon | Both | 2021 | 423.78 | 570.45 | 307.13 |
| ASIR | Cabo Verde | Both | 2021 | 453.74 | 609.22 | 330.14 |
| ASIR | Gambia | Both | 2021 | 450.67 | 607.84 | 325.83 |
| ASIR | Chad | Both | 2021 | 346.07 | 460.42 | 256.39 |
| ASIR | Coted'Ivoire | Both | 2021 | 430.20 | 572.06 | 316.08 |
| ASIR | Ghana | Both | 2021 | 425.31 | 574.94 | 304.43 |
| ASIR | Guinea | Both | 2021 | 357.00 | 475.70 | 264.09 |
| ASIR | Guinea-Bissau | Both | 2021 | 397.94 | 531.74 | 290.54 |
| ASIR | Mali | Both | 2021 | 439.09 | 599.12 | 314.05 |
| ASIR | Mauritania | Both | 2021 | 407.70 | 544.86 | 296.83 |
| ASIR | Liberia | Both | 2021 | 368.09 | 499.94 | 263.79 |
| ASIR | Niger | Both | 2021 | 435.07 | 591.87 | 312.59 |
| ASIR | Senegal | Both | 2021 | 412.52 | 557.59 | 298.48 |
| ASIR | Nigeria | Both | 2021 | 445.22 | 631.81 | 305.30 |
| ASIR | Sao Tome and Principe | Both | 2021 | 595.77 | 820.67 | 418.00 |
| ASIR | Sierra Leone | Both | 2021 | 348.41 | 469.97 | 253.69 |
| ASIR | Togo | Both | 2021 | 404.41 | 545.68 | 295.13 |
| ASIR | Bermuda | Both | 2021 | 458.76 | 620.80 | 328.50 |
| ASIR | American Samoa | Both | 2021 | 427.16 | 572.42 | 308.34 |
| ASIR | Cook Islands | Both | 2021 | 349.50 | 475.96 | 250.83 |
| ASIR | Greenland | Both | 2021 | 2386.60 | 3238.58 | 1691.64 |
| ASIR | Guam | Both | 2021 | 296.57 | 400.74 | 212.75 |
| ASIR | Monaco | Both | 2021 | 1055.30 | 1438.38 | 741.17 |
| ASIR | Palau | Both | 2021 | 799.68 | 1089.44 | 564.59 |
| ASIR | Nauru | Both | 2021 | 488.13 | 668.09 | 346.45 |
| ASIR | Puerto Rico | Both | 2021 | 544.37 | 747.27 | 385.18 |
| ASIR | Niue | Both | 2021 | 415.72 | 562.00 | 297.29 |
| ASIR | Saint Kitts and Nevis | Both | 2021 | 512.23 | 691.99 | 371.95 |
| ASIR | Northern Mariana Islands | Both | 2021 | 732.33 | 999.73 | 523.48 |
| ASIR | Tuvalu | Both | 2021 | 391.02 | 525.66 | 282.80 |
| ASIR | San Marino | Both | 2021 | 1181.11 | 1610.57 | 830.88 |
| ASIR | United States Virgin Islands | Both | 2021 | 511.38 | 691.91 | 369.78 |
| ASIR | Tokelau | Both | 2021 | 395.98 | 539.44 | 283.33 |
| ASIR | South Sudan | Both | 2021 | 317.15 | 422.04 | 235.51 |
| ASIR | Sudan | Both | 2021 | 306.69 | 403.88 | 228.08 |
| ASPR | China | Both | 2021 | 1335.45 | 1675.35 | 1063.52 |
| ASPR | Taiwan (Province of China) | Both | 2021 | 741.26 | 894.19 | 611.86 |
| ASPR | Cambodia | Both | 2021 | 1215.73 | 1685.68 | 927.52 |
| ASPR | Democratic People's Republic of Korea | Both | 2021 | 425.06 | 490.44 | 366.96 |
| ASPR | Malaysia | Both | 2021 | 739.98 | 904.89 | 605.09 |
| ASPR | Indonesia | Both | 2021 | 798.57 | 1013.78 | 632.42 |
| ASPR | Lao People's Democratic Republic | Both | 2021 | 504.16 | 607.71 | 418.51 |
| ASPR | Maldives | Both | 2021 | 765.12 | 939.01 | 615.87 |
| ASPR | Myanmar | Both | 2021 | 921.66 | 1135.73 | 748.53 |
| ASPR | Thailand | Both | 2021 | 802.63 | 970.76 | 670.17 |
| ASPR | Philippines | Both | 2021 | 551.21 | 691.25 | 446.41 |
| ASPR | Timor-Leste | Both | 2021 | 699.38 | 1016.36 | 505.38 |
| ASPR | Sri Lanka | Both | 2021 | 1322.74 | 1635.07 | 1066.14 |
| ASPR | Viet Nam | Both | 2021 | 1565.30 | 1943.78 | 1256.93 |
| ASPR | Kiribati | Both | 2021 | 233.95 | 281.35 | 193.67 |
| ASPR | Fiji | Both | 2021 | 357.35 | 431.25 | 295.42 |
| ASPR | Marshall Islands | Both | 2021 | 450.05 | 548.74 | 368.63 |
| ASPR | Micronesia (Federated States of) | Both | 2021 | 551.47 | 667.08 | 451.86 |
| ASPR | Solomon Islands | Both | 2021 | 1187.99 | 1495.47 | 935.35 |
| ASPR | Papua New Guinea | Both | 2021 | 1089.07 | 1337.44 | 877.04 |
| ASPR | Tonga | Both | 2021 | 496.70 | 593.82 | 410.11 |
| ASPR | Samoa | Both | 2021 | 660.53 | 800.59 | 542.50 |
| ASPR | Armenia | Both | 2021 | 535.52 | 669.98 | 441.38 |
| ASPR | Vanuatu | Both | 2021 | 387.57 | 464.49 | 321.56 |
| ASPR | Azerbaijan | Both | 2021 | 298.22 | 354.25 | 251.69 |
| ASPR | Kyrgyzstan | Both | 2021 | 399.80 | 462.81 | 346.56 |
| ASPR | Georgia | Both | 2021 | 800.76 | 961.14 | 666.63 |
| ASPR | Mongolia | Both | 2021 | 490.69 | 580.32 | 415.73 |
| ASPR | Kazakhstan | Both | 2021 | 594.80 | 706.39 | 503.51 |
| ASPR | Tajikistan | Both | 2021 | 307.94 | 367.38 | 261.33 |
| ASPR | Turkmenistan | Both | 2021 | 271.41 | 319.71 | 231.59 |
| ASPR | Albania | Both | 2021 | 756.17 | 900.14 | 636.79 |
| ASPR | Uzbekistan | Both | 2021 | 301.20 | 354.45 | 256.09 |
| ASPR | Czechia | Both | 2021 | 2394.21 | 2925.60 | 1946.88 |
| ASPR | Bosnia and Herzegovina | Both | 2021 | 962.50 | 1169.23 | 787.37 |
| ASPR | Hungary | Both | 2021 | 2400.86 | 2990.59 | 1921.46 |
| ASPR | Bulgaria | Both | 2021 | 792.25 | 952.16 | 661.63 |
| ASPR | Croatia | Both | 2021 | 3552.24 | 4349.33 | 2877.37 |
| ASPR | North Macedonia | Both | 2021 | 1079.14 | 1322.07 | 875.58 |
| ASPR | Romania | Both | 2021 | 984.79 | 1183.75 | 817.67 |
| ASPR | Montenegro | Both | 2021 | 1269.34 | 1569.35 | 1034.48 |
| ASPR | Serbia | Both | 2021 | 1091.91 | 1335.95 | 891.52 |
| ASPR | Poland | Both | 2021 | 1743.38 | 2181.48 | 1399.03 |
| ASPR | Slovakia | Both | 2021 | 2148.77 | 2625.11 | 1744.22 |
| ASPR | Belarus | Both | 2021 | 1049.42 | 1268.37 | 864.75 |
| ASPR | Slovenia | Both | 2021 | 3763.04 | 4550.10 | 3074.43 |
| ASPR | Estonia | Both | 2021 | 1030.71 | 1244.08 | 852.33 |
| ASPR | Latvia | Both | 2021 | 1119.67 | 1345.47 | 922.03 |
| ASPR | Russian Federation | Both | 2021 | 995.54 | 1218.24 | 824.02 |
| ASPR | Lithuania | Both | 2021 | 1487.56 | 1787.53 | 1222.83 |
| ASPR | Ukraine | Both | 2021 | 779.55 | 953.15 | 648.11 |
| ASPR | Republic of Moldova | Both | 2021 | 681.60 | 816.49 | 569.49 |
| ASPR | Japan | Both | 2021 | 2098.57 | 2558.53 | 1709.41 |
| ASPR | Brunei Darussalam | Both | 2021 | 2051.39 | 2438.29 | 1712.26 |
| ASPR | Republic of Korea | Both | 2021 | 4304.02 | 5140.05 | 3580.40 |
| ASPR | Singapore | Both | 2021 | 1894.18 | 2257.65 | 1587.10 |
| ASPR | New Zealand | Both | 2021 | 4519.36 | 5480.64 | 3701.67 |
| ASPR | Australia | Both | 2021 | 5157.76 | 6120.96 | 4320.54 |
| ASPR | Andorra | Both | 2021 | 7377.84 | 8749.68 | 6169.25 |
| ASPR | Cyprus | Both | 2021 | 4188.82 | 5011.39 | 3462.20 |
| ASPR | Denmark | Both | 2021 | 3688.85 | 4383.62 | 3081.44 |
| ASPR | Austria | Both | 2021 | 4605.33 | 5513.96 | 3807.12 |
| ASPR | Belgium | Both | 2021 | 6265.82 | 7477.08 | 5200.26 |
| ASPR | Finland | Both | 2021 | 5764.37 | 6861.93 | 4782.69 |
| ASPR | France | Both | 2021 | 5622.39 | 6669.57 | 4691.42 |
| ASPR | Greece | Both | 2021 | 1908.88 | 2266.47 | 1591.28 |
| ASPR | Iceland | Both | 2021 | 3512.94 | 4180.46 | 2920.50 |
| ASPR | Ireland | Both | 2021 | 3222.98 | 3833.18 | 2675.96 |
| ASPR | Luxembourg | Both | 2021 | 4762.51 | 5653.65 | 3981.13 |
| ASPR | Germany | Both | 2021 | 4235.20 | 5049.73 | 3489.24 |
| ASPR | Israel | Both | 2021 | 2260.28 | 2652.26 | 1900.51 |
| ASPR | Italy | Both | 2021 | 3909.52 | 4787.23 | 3173.80 |
| ASPR | Malta | Both | 2021 | 3395.74 | 4063.49 | 2827.00 |
| ASPR | Spain | Both | 2021 | 2900.25 | 3480.61 | 2402.65 |
| ASPR | Netherlands | Both | 2021 | 5438.80 | 6478.15 | 4510.72 |
| ASPR | Norway | Both | 2021 | 5636.81 | 6877.64 | 4591.57 |
| ASPR | Sweden | Both | 2021 | 4463.96 | 5482.77 | 3600.52 |
| ASPR | Portugal | Both | 2021 | 2596.65 | 3108.51 | 2147.92 |
| ASPR | Argentina | Both | 2021 | 1492.84 | 1774.66 | 1255.67 |
| ASPR | Switzerland | Both | 2021 | 5934.09 | 7027.53 | 4974.57 |
| ASPR | Chile | Both | 2021 | 2902.16 | 3474.06 | 2396.01 |
| ASPR | United Kingdom | Both | 2021 | 3421.28 | 4207.46 | 2767.26 |
| ASPR | Canada | Both | 2021 | 5039.09 | 6014.90 | 4186.80 |
| ASPR | United States of America | Both | 2021 | 4333.33 | 5414.28 | 3444.66 |
| ASPR | Uruguay | Both | 2021 | 2205.44 | 2623.15 | 1838.98 |
| ASPR | Bahamas | Both | 2021 | 651.71 | 783.34 | 540.64 |
| ASPR | Antigua and Barbuda | Both | 2021 | 582.22 | 696.78 | 488.65 |
| ASPR | Dominican Republic | Both | 2021 | 561.37 | 662.27 | 476.61 |
| ASPR | Barbados | Both | 2021 | 580.10 | 701.35 | 479.96 |
| ASPR | Grenada | Both | 2021 | 631.25 | 786.87 | 514.91 |
| ASPR | Belize | Both | 2021 | 590.70 | 698.21 | 503.90 |
| ASPR | Cuba | Both | 2021 | 2525.33 | 3067.23 | 2048.71 |
| ASPR | Guyana | Both | 2021 | 716.13 | 864.48 | 591.83 |
| ASPR | Dominica | Both | 2021 | 434.14 | 519.19 | 365.56 |
| ASPR | Saint Lucia | Both | 2021 | 508.70 | 599.96 | 434.18 |
| ASPR | Saint Vincent and the Grenadines | Both | 2021 | 539.88 | 639.12 | 454.03 |
| ASPR | Haiti | Both | 2021 | 543.71 | 828.70 | 396.25 |
| ASPR | Suriname | Both | 2021 | 595.82 | 716.82 | 500.53 |
| ASPR | Jamaica | Both | 2021 | 479.78 | 572.62 | 403.92 |
| ASPR | Bolivia (Plurinational State of) | Both | 2021 | 649.04 | 771.78 | 545.51 |
| ASPR | Ecuador | Both | 2021 | 745.48 | 878.82 | 633.52 |
| ASPR | Trinidad and Tobago | Both | 2021 | 422.47 | 496.41 | 359.10 |
| ASPR | Peru | Both | 2021 | 599.17 | 717.00 | 503.78 |
| ASPR | Costa Rica | Both | 2021 | 1315.29 | 1580.24 | 1084.83 |
| ASPR | Colombia | Both | 2021 | 673.54 | 799.24 | 568.61 |
| ASPR | El Salvador | Both | 2021 | 1111.79 | 1463.71 | 877.82 |
| ASPR | Guatemala | Both | 2021 | 968.76 | 1221.73 | 788.15 |
| ASPR | Nicaragua | Both | 2021 | 1261.91 | 1690.31 | 976.60 |
| ASPR | Honduras | Both | 2021 | 576.79 | 676.40 | 493.08 |
| ASPR | Panama | Both | 2021 | 590.67 | 692.62 | 501.78 |
| ASPR | Paraguay | Both | 2021 | 802.03 | 953.42 | 673.15 |
| ASPR | Venezuela (Bolivarian Republic of) | Both | 2021 | 776.73 | 922.28 | 649.07 |
| ASPR | Mexico | Both | 2021 | 1064.83 | 1322.77 | 866.18 |
| ASPR | Brazil | Both | 2021 | 1187.52 | 1466.96 | 967.61 |
| ASPR | Egypt | Both | 2021 | 436.96 | 514.07 | 374.61 |
| ASPR | Algeria | Both | 2021 | 644.56 | 750.20 | 553.83 |
| ASPR | Iran (Islamic Republic of) | Both | 2021 | 781.92 | 969.39 | 646.12 |
| ASPR | Iraq | Both | 2021 | 738.42 | 1129.76 | 518.05 |
| ASPR | Lebanon | Both | 2021 | 753.14 | 1181.64 | 535.39 |
| ASPR | Bahrain | Both | 2021 | 433.96 | 515.70 | 364.57 |
| ASPR | Jordan | Both | 2021 | 477.96 | 569.48 | 402.73 |
| ASPR | Libya | Both | 2021 | 969.80 | 1177.49 | 810.35 |
| ASPR | Kuwait | Both | 2021 | 545.09 | 642.75 | 462.49 |
| ASPR | Qatar | Both | 2021 | 776.72 | 930.27 | 647.40 |
| ASPR | Morocco | Both | 2021 | 814.68 | 993.65 | 667.70 |
| ASPR | Saudi Arabia | Both | 2021 | 1788.89 | 2163.67 | 1466.48 |
| ASPR | Palestine | Both | 2021 | 756.26 | 1074.10 | 566.71 |
| ASPR | Oman | Both | 2021 | 1134.82 | 1386.09 | 928.05 |
| ASPR | Syrian Arab Republic | Both | 2021 | 588.13 | 804.37 | 455.20 |
| ASPR | United Arab Emirates | Both | 2021 | 584.55 | 665.38 | 511.84 |
| ASPR | Tunisia | Both | 2021 | 658.91 | 782.26 | 556.11 |
| ASPR | Yemen | Both | 2021 | 560.76 | 672.64 | 475.69 |
| ASPR | Turkey | Both | 2021 | 998.96 | 1220.68 | 814.89 |
| ASPR | Afghanistan | Both | 2021 | 735.89 | 1454.73 | 422.77 |
| ASPR | Bangladesh | Both | 2021 | 277.02 | 329.76 | 233.97 |
| ASPR | Bhutan | Both | 2021 | 1327.37 | 1674.46 | 1045.84 |
| ASPR | Pakistan | Both | 2021 | 333.70 | 406.10 | 275.11 |
| ASPR | India | Both | 2021 | 1813.54 | 2350.54 | 1402.08 |
| ASPR | Nepal | Both | 2021 | 1080.16 | 1366.70 | 855.53 |
| ASPR | Angola | Both | 2021 | 543.08 | 698.43 | 434.80 |
| ASPR | Congo | Both | 2021 | 470.37 | 573.88 | 386.35 |
| ASPR | Democratic Republic of the Congo | Both | 2021 | 498.18 | 598.71 | 410.77 |
| ASPR | Central African Republic | Both | 2021 | 346.39 | 420.12 | 284.80 |
| ASPR | Equatorial Guinea | Both | 2021 | 549.09 | 669.41 | 447.90 |
| ASPR | Burundi | Both | 2021 | 473.43 | 592.90 | 386.00 |
| ASPR | Gabon | Both | 2021 | 593.26 | 726.36 | 484.98 |
| ASPR | Comoros | Both | 2021 | 483.57 | 577.11 | 407.43 |
| ASPR | Djibouti | Both | 2021 | 488.33 | 586.58 | 408.02 |
| ASPR | Kenya | Both | 2021 | 639.46 | 793.59 | 518.83 |
| ASPR | Eritrea | Both | 2021 | 633.88 | 925.60 | 457.08 |
| ASPR | Madagascar | Both | 2021 | 347.67 | 419.41 | 288.89 |
| ASPR | Ethiopia | Both | 2021 | 579.50 | 719.32 | 471.45 |
| ASPR | Malawi | Both | 2021 | 496.36 | 612.24 | 405.28 |
| ASPR | Rwanda | Both | 2021 | 643.00 | 892.86 | 490.92 |
| ASPR | Mauritius | Both | 2021 | 391.49 | 469.54 | 326.31 |
| ASPR | Seychelles | Both | 2021 | 438.20 | 525.83 | 365.04 |
| ASPR | Mozambique | Both | 2021 | 504.87 | 654.07 | 398.98 |
| ASPR | Somalia | Both | 2021 | 379.57 | 473.11 | 314.01 |
| ASPR | Zambia | Both | 2021 | 540.73 | 660.84 | 442.74 |
| ASPR | United Republic of Tanzania | Both | 2021 | 519.24 | 635.14 | 425.69 |
| ASPR | Uganda | Both | 2021 | 555.13 | 697.63 | 448.12 |
| ASPR | Botswana | Both | 2021 | 378.88 | 461.57 | 312.21 |
| ASPR | South Africa | Both | 2021 | 305.98 | 361.56 | 259.66 |
| ASPR | Lesotho | Both | 2021 | 308.83 | 372.31 | 255.91 |
| ASPR | Eswatini | Both | 2021 | 301.59 | 358.88 | 251.67 |
| ASPR | Namibia | Both | 2021 | 387.58 | 469.24 | 322.85 |
| ASPR | Zimbabwe | Both | 2021 | 399.27 | 483.25 | 327.29 |
| ASPR | Benin | Both | 2021 | 554.07 | 668.68 | 458.62 |
| ASPR | Burkina Faso | Both | 2021 | 597.35 | 722.75 | 496.53 |
| ASPR | Cameroon | Both | 2021 | 583.67 | 719.12 | 472.27 |
| ASPR | Cabo Verde | Both | 2021 | 649.20 | 787.83 | 531.91 |
| ASPR | Gambia | Both | 2021 | 609.11 | 757.27 | 492.23 |
| ASPR | Chad | Both | 2021 | 454.19 | 559.52 | 372.72 |
| ASPR | Coted'Ivoire | Both | 2021 | 563.57 | 681.76 | 465.11 |
| ASPR | Ghana | Both | 2021 | 589.52 | 726.59 | 480.39 |
| ASPR | Guinea | Both | 2021 | 463.26 | 556.66 | 385.08 |
| ASPR | Guinea-Bissau | Both | 2021 | 486.88 | 592.24 | 398.28 |
| ASPR | Mali | Both | 2021 | 606.58 | 744.92 | 492.74 |
| ASPR | Mauritania | Both | 2021 | 580.29 | 701.61 | 481.19 |
| ASPR | Liberia | Both | 2021 | 526.54 | 654.53 | 426.83 |
| ASPR | Niger | Both | 2021 | 594.78 | 731.44 | 483.72 |
| ASPR | Senegal | Both | 2021 | 584.06 | 716.82 | 476.09 |
| ASPR | Nigeria | Both | 2021 | 631.36 | 794.04 | 504.84 |
| ASPR | Sao Tome and Principe | Both | 2021 | 826.14 | 1028.30 | 662.94 |
| ASPR | Sierra Leone | Both | 2021 | 485.34 | 587.28 | 399.57 |
| ASPR | Togo | Both | 2021 | 561.92 | 682.13 | 464.45 |
| ASPR | Bermuda | Both | 2021 | 733.90 | 887.69 | 606.97 |
| ASPR | American Samoa | Both | 2021 | 577.51 | 698.08 | 471.64 |
| ASPR | Cook Islands | Both | 2021 | 575.97 | 691.33 | 472.57 |
| ASPR | Greenland | Both | 2021 | 5102.25 | 6207.41 | 4164.08 |
| ASPR | Guam | Both | 2021 | 479.70 | 581.12 | 394.97 |
| ASPR | Monaco | Both | 2021 | 2475.40 | 2981.11 | 2031.25 |
| ASPR | Palau | Both | 2021 | 1072.55 | 1330.68 | 865.66 |
| ASPR | Nauru | Both | 2021 | 580.65 | 711.67 | 470.80 |
| ASPR | Puerto Rico | Both | 2021 | 1052.83 | 1286.87 | 864.08 |
| ASPR | Niue | Both | 2021 | 578.21 | 706.69 | 470.93 |
| ASPR | Saint Kitts and Nevis | Both | 2021 | 673.21 | 809.98 | 556.93 |
| ASPR | Northern Mariana Islands | Both | 2021 | 1066.50 | 1280.25 | 874.79 |
| ASPR | Tuvalu | Both | 2021 | 538.36 | 649.44 | 444.53 |
| ASPR | San Marino | Both | 2021 | 2929.55 | 3517.72 | 2413.49 |
| ASPR | United States Virgin Islands | Both | 2021 | 706.06 | 847.61 | 587.87 |
| ASPR | Tokelau | Both | 2021 | 547.41 | 662.18 | 447.96 |
| ASPR | South Sudan | Both | 2021 | 379.62 | 470.87 | 312.18 |
| ASPR | Sudan | Both | 2021 | 500.36 | 592.97 | 427.35 |
| ASYR | China | Both | 2021 | 113.90 | 165.51 | 73.89 |
| ASYR | Taiwan (Province of China) | Both | 2021 | 53.88 | 77.38 | 35.04 |
| ASYR | Cambodia | Both | 2021 | 235.50 | 356.89 | 150.14 |
| ASYR | Democratic People's Republic of Korea | Both | 2021 | 64.17 | 89.06 | 43.20 |
| ASYR | Malaysia | Both | 2021 | 100.49 | 140.50 | 67.18 |
| ASYR | Indonesia | Both | 2021 | 145.24 | 204.57 | 96.76 |
| ASYR | Lao People's Democratic Republic | Both | 2021 | 107.71 | 149.88 | 70.72 |
| ASYR | Maldives | Both | 2021 | 91.01 | 127.99 | 59.92 |
| ASYR | Myanmar | Both | 2021 | 178.37 | 249.12 | 119.21 |
| ASYR | Thailand | Both | 2021 | 85.19 | 118.24 | 57.17 |
| ASYR | Philippines | Both | 2021 | 100.58 | 139.99 | 67.93 |
| ASYR | Timor-Leste | Both | 2021 | 143.66 | 227.00 | 86.26 |
| ASYR | Sri Lanka | Both | 2021 | 152.39 | 212.54 | 102.63 |
| ASYR | Viet Nam | Both | 2021 | 206.55 | 290.27 | 137.23 |
| ASYR | Kiribati | Both | 2021 | 55.99 | 80.02 | 36.18 |
| ASYR | Fiji | Both | 2021 | 68.61 | 96.30 | 44.92 |
| ASYR | Marshall Islands | Both | 2021 | 96.11 | 135.23 | 63.25 |
| ASYR | Micronesia (Federated States of) | Both | 2021 | 111.82 | 156.73 | 74.06 |
| ASYR | Solomon Islands | Both | 2021 | 261.69 | 371.51 | 171.16 |
| ASYR | Papua New Guinea | Both | 2021 | 236.07 | 330.05 | 155.55 |
| ASYR | Tonga | Both | 2021 | 83.15 | 114.92 | 55.74 |
| ASYR | Samoa | Both | 2021 | 114.35 | 158.42 | 76.09 |
| ASYR | Armenia | Both | 2021 | 60.71 | 85.97 | 40.50 |
| ASYR | Vanuatu | Both | 2021 | 84.87 | 119.88 | 55.94 |
| ASYR | Azerbaijan | Both | 2021 | 44.56 | 62.59 | 29.79 |
| ASYR | Kyrgyzstan | Both | 2021 | 57.67 | 79.44 | 39.01 |
| ASYR | Georgia | Both | 2021 | 106.42 | 147.62 | 72.05 |
| ASYR | Mongolia | Both | 2021 | 83.27 | 115.73 | 55.77 |
| ASYR | Kazakhstan | Both | 2021 | 74.77 | 102.73 | 50.51 |
| ASYR | Tajikistan | Both | 2021 | 58.41 | 82.61 | 38.39 |
| ASYR | Turkmenistan | Both | 2021 | 44.26 | 62.15 | 28.80 |
| ASYR | Albania | Both | 2021 | 70.28 | 99.76 | 46.44 |
| ASYR | Uzbekistan | Both | 2021 | 50.38 | 70.38 | 33.32 |
| ASYR | Czechia | Both | 2021 | 184.95 | 269.22 | 120.43 |
| ASYR | Bosnia and Herzegovina | Both | 2021 | 88.90 | 126.21 | 59.30 |
| ASYR | Hungary | Both | 2021 | 187.01 | 271.93 | 119.97 |
| ASYR | Bulgaria | Both | 2021 | 86.41 | 120.75 | 57.83 |
| ASYR | Croatia | Both | 2021 | 283.45 | 413.33 | 180.81 |
| ASYR | North Macedonia | Both | 2021 | 112.24 | 161.22 | 73.66 |
| ASYR | Romania | Both | 2021 | 87.68 | 124.59 | 58.20 |
| ASYR | Montenegro | Both | 2021 | 100.65 | 147.17 | 65.24 |
| ASYR | Serbia | Both | 2021 | 90.91 | 132.98 | 58.64 |
| ASYR | Poland | Both | 2021 | 137.25 | 199.84 | 88.57 |
| ASYR | Slovakia | Both | 2021 | 165.59 | 238.72 | 107.29 |
| ASYR | Belarus | Both | 2021 | 83.45 | 119.39 | 54.55 |
| ASYR | Slovenia | Both | 2021 | 287.12 | 414.78 | 186.64 |
| ASYR | Estonia | Both | 2021 | 75.99 | 109.61 | 49.64 |
| ASYR | Latvia | Both | 2021 | 99.26 | 141.37 | 66.55 |
| ASYR | Russian Federation | Both | 2021 | 87.23 | 123.23 | 58.83 |
| ASYR | Lithuania | Both | 2021 | 135.13 | 188.83 | 89.12 |
| ASYR | Ukraine | Both | 2021 | 86.31 | 120.67 | 58.38 |
| ASYR | Republic of Moldova | Both | 2021 | 75.23 | 104.69 | 50.56 |
| ASYR | Japan | Both | 2021 | 139.78 | 200.54 | 91.10 |
| ASYR | Brunei Darussalam | Both | 2021 | 259.77 | 355.34 | 179.18 |
| ASYR | Republic of Korea | Both | 2021 | 293.15 | 416.52 | 194.14 |
| ASYR | Singapore | Both | 2021 | 124.57 | 177.86 | 83.03 |
| ASYR | New Zealand | Both | 2021 | 323.35 | 465.94 | 210.61 |
| ASYR | Australia | Both | 2021 | 366.74 | 519.90 | 246.19 |
| ASYR | Andorra | Both | 2021 | 519.08 | 740.57 | 347.60 |
| ASYR | Cyprus | Both | 2021 | 308.76 | 443.15 | 204.16 |
| ASYR | Denmark | Both | 2021 | 264.78 | 377.55 | 177.34 |
| ASYR | Austria | Both | 2021 | 317.03 | 452.26 | 209.25 |
| ASYR | Belgium | Both | 2021 | 428.74 | 607.82 | 285.60 |
| ASYR | Finland | Both | 2021 | 394.37 | 560.28 | 260.94 |
| ASYR | France | Both | 2021 | 388.16 | 550.36 | 258.10 |
| ASYR | Greece | Both | 2021 | 126.32 | 178.84 | 83.11 |
| ASYR | Iceland | Both | 2021 | 242.27 | 341.91 | 162.36 |
| ASYR | Ireland | Both | 2021 | 221.30 | 313.99 | 146.03 |
| ASYR | Luxembourg | Both | 2021 | 332.01 | 473.60 | 221.32 |
| ASYR | Germany | Both | 2021 | 294.26 | 418.85 | 195.28 |
| ASYR | Israel | Both | 2021 | 154.78 | 219.40 | 103.44 |
| ASYR | Italy | Both | 2021 | 266.70 | 382.95 | 173.61 |
| ASYR | Malta | Both | 2021 | 232.50 | 332.24 | 154.01 |
| ASYR | Spain | Both | 2021 | 193.70 | 273.62 | 128.47 |
| ASYR | Netherlands | Both | 2021 | 394.23 | 561.86 | 264.43 |
| ASYR | Norway | Both | 2021 | 406.10 | 588.61 | 264.26 |
| ASYR | Sweden | Both | 2021 | 315.18 | 456.98 | 204.40 |
| ASYR | Portugal | Both | 2021 | 174.21 | 247.67 | 116.55 |
| ASYR | Argentina | Both | 2021 | 177.99 | 241.67 | 123.27 |
| ASYR | Switzerland | Both | 2021 | 416.25 | 588.04 | 278.29 |
| ASYR | Chile | Both | 2021 | 222.80 | 314.18 | 148.66 |
| ASYR | United Kingdom | Both | 2021 | 237.10 | 340.24 | 153.74 |
| ASYR | Canada | Both | 2021 | 352.76 | 500.95 | 235.37 |
| ASYR | United States of America | Both | 2021 | 287.22 | 414.31 | 184.82 |
| ASYR | Uruguay | Both | 2021 | 219.38 | 300.50 | 151.74 |
| ASYR | Bahamas | Both | 2021 | 96.69 | 134.42 | 64.63 |
| ASYR | Antigua and Barbuda | Both | 2021 | 77.23 | 109.16 | 51.17 |
| ASYR | Dominican Republic | Both | 2021 | 96.09 | 132.91 | 64.69 |
| ASYR | Barbados | Both | 2021 | 73.10 | 102.72 | 48.53 |
| ASYR | Grenada | Both | 2021 | 100.25 | 142.59 | 66.49 |
| ASYR | Belize | Both | 2021 | 95.75 | 133.02 | 64.62 |
| ASYR | Cuba | Both | 2021 | 252.70 | 356.19 | 165.42 |
| ASYR | Guyana | Both | 2021 | 142.40 | 198.07 | 94.60 |
| ASYR | Dominica | Both | 2021 | 74.89 | 105.25 | 49.44 |
| ASYR | Saint Lucia | Both | 2021 | 75.56 | 104.76 | 50.90 |
| ASYR | Saint Vincent and the Grenadines | Both | 2021 | 89.67 | 124.63 | 59.65 |
| ASYR | Haiti | Both | 2021 | 134.25 | 234.71 | 81.02 |
| ASYR | Suriname | Both | 2021 | 107.48 | 147.95 | 72.30 |
| ASYR | Jamaica | Both | 2021 | 67.85 | 94.10 | 45.75 |
| ASYR | Bolivia (Plurinational State of) | Both | 2021 | 123.12 | 171.39 | 82.66 |
| ASYR | Ecuador | Both | 2021 | 109.70 | 150.69 | 75.05 |
| ASYR | Trinidad and Tobago | Both | 2021 | 63.01 | 87.12 | 42.47 |
| ASYR | Peru | Both | 2021 | 72.95 | 101.73 | 49.53 |
| ASYR | Costa Rica | Both | 2021 | 134.62 | 186.50 | 90.79 |
| ASYR | Colombia | Both | 2021 | 77.79 | 106.88 | 52.78 |
| ASYR | El Salvador | Both | 2021 | 151.21 | 218.13 | 99.33 |
| ASYR | Guatemala | Both | 2021 | 173.06 | 247.02 | 115.29 |
| ASYR | Nicaragua | Both | 2021 | 184.40 | 277.39 | 120.34 |
| ASYR | Honduras | Both | 2021 | 112.63 | 156.49 | 76.38 |
| ASYR | Panama | Both | 2021 | 70.31 | 96.06 | 47.81 |
| ASYR | Paraguay | Both | 2021 | 119.15 | 164.45 | 80.65 |
| ASYR | Venezuela (Bolivarian Republic of) | Both | 2021 | 112.62 | 155.12 | 76.34 |
| ASYR | Mexico | Both | 2021 | 153.13 | 211.82 | 104.16 |
| ASYR | Brazil | Both | 2021 | 166.62 | 230.36 | 114.10 |
| ASYR | Egypt | Both | 2021 | 65.93 | 90.83 | 44.40 |
| ASYR | Algeria | Both | 2021 | 79.19 | 109.27 | 53.91 |
| ASYR | Iran (Islamic Republic of) | Both | 2021 | 76.29 | 106.71 | 51.82 |
| ASYR | Iraq | Both | 2021 | 90.74 | 144.19 | 56.01 |
| ASYR | Lebanon | Both | 2021 | 60.45 | 98.80 | 36.87 |
| ASYR | Bahrain | Both | 2021 | 41.46 | 58.88 | 27.34 |
| ASYR | Jordan | Both | 2021 | 49.80 | 69.35 | 33.17 |
| ASYR | Libya | Both | 2021 | 130.03 | 186.26 | 84.36 |
| ASYR | Kuwait | Both | 2021 | 40.20 | 57.47 | 26.52 |
| ASYR | Qatar | Both | 2021 | 63.41 | 92.90 | 41.39 |
| ASYR | Morocco | Both | 2021 | 127.98 | 178.05 | 85.96 |
| ASYR | Saudi Arabia | Both | 2021 | 186.61 | 259.83 | 124.94 |
| ASYR | Palestine | Both | 2021 | 94.13 | 141.69 | 59.37 |
| ASYR | Oman | Both | 2021 | 116.69 | 167.56 | 76.57 |
| ASYR | Syrian Arab Republic | Both | 2021 | 70.04 | 104.22 | 45.64 |
| ASYR | United Arab Emirates | Both | 2021 | 72.46 | 98.92 | 49.25 |
| ASYR | Tunisia | Both | 2021 | 69.24 | 96.14 | 46.65 |
| ASYR | Yemen | Both | 2021 | 110.29 | 152.52 | 74.40 |
| ASYR | Turkey | Both | 2021 | 100.76 | 141.25 | 66.09 |
| ASYR | Afghanistan | Both | 2021 | 165.47 | 313.68 | 82.82 |
| ASYR | Bangladesh | Both | 2021 | 47.74 | 67.41 | 31.75 |
| ASYR | Bhutan | Both | 2021 | 236.70 | 335.57 | 155.73 |
| ASYR | Pakistan | Both | 2021 | 69.45 | 96.99 | 46.33 |
| ASYR | India | Both | 2021 | 313.14 | 442.22 | 206.21 |
| ASYR | Nepal | Both | 2021 | 203.56 | 286.52 | 133.66 |
| ASYR | Angola | Both | 2021 | 119.72 | 172.76 | 77.54 |
| ASYR | Congo | Both | 2021 | 99.71 | 142.08 | 65.89 |
| ASYR | Democratic Republic of the Congo | Both | 2021 | 113.05 | 158.26 | 75.27 |
| ASYR | Central African Republic | Both | 2021 | 92.73 | 131.96 | 60.13 |
| ASYR | Equatorial Guinea | Both | 2021 | 98.89 | 138.29 | 65.35 |
| ASYR | Burundi | Both | 2021 | 115.30 | 165.17 | 75.35 |
| ASYR | Gabon | Both | 2021 | 112.99 | 159.20 | 75.23 |
| ASYR | Comoros | Both | 2021 | 105.52 | 146.87 | 70.75 |
| ASYR | Djibouti | Both | 2021 | 104.18 | 146.60 | 69.38 |
| ASYR | Kenya | Both | 2021 | 128.70 | 178.92 | 86.41 |
| ASYR | Eritrea | Both | 2021 | 152.12 | 244.60 | 90.81 |
| ASYR | Madagascar | Both | 2021 | 80.40 | 114.48 | 52.42 |
| ASYR | Ethiopia | Both | 2021 | 122.62 | 170.94 | 81.69 |
| ASYR | Malawi | Both | 2021 | 110.66 | 157.27 | 73.10 |
| ASYR | Rwanda | Both | 2021 | 140.24 | 212.85 | 88.43 |
| ASYR | Mauritius | Both | 2021 | 51.86 | 73.24 | 34.83 |
| ASYR | Seychelles | Both | 2021 | 62.88 | 88.34 | 42.23 |
| ASYR | Mozambique | Both | 2021 | 119.50 | 172.80 | 77.68 |
| ASYR | Somalia | Both | 2021 | 99.38 | 142.14 | 64.96 |
| ASYR | Zambia | Both | 2021 | 117.68 | 166.46 | 77.80 |
| ASYR | United Republic of Tanzania | Both | 2021 | 110.93 | 156.19 | 73.69 |
| ASYR | Uganda | Both | 2021 | 119.40 | 170.42 | 78.30 |
| ASYR | Botswana | Both | 2021 | 74.98 | 106.98 | 49.55 |
| ASYR | South Africa | Both | 2021 | 53.34 | 72.58 | 36.50 |
| ASYR | Lesotho | Both | 2021 | 72.62 | 103.26 | 47.26 |
| ASYR | Eswatini | Both | 2021 | 64.42 | 91.25 | 41.76 |
| ASYR | Namibia | Both | 2021 | 74.30 | 104.35 | 48.85 |
| ASYR | Zimbabwe | Both | 2021 | 90.95 | 127.96 | 59.22 |
| ASYR | Benin | Both | 2021 | 120.76 | 170.42 | 80.76 |
| ASYR | Burkina Faso | Both | 2021 | 137.47 | 191.95 | 91.71 |
| ASYR | Cameroon | Both | 2021 | 123.47 | 174.32 | 81.63 |
| ASYR | Cabo Verde | Both | 2021 | 101.29 | 141.86 | 67.46 |
| ASYR | Gambia | Both | 2021 | 127.46 | 180.77 | 84.23 |
| ASYR | Chad | Both | 2021 | 110.27 | 155.05 | 72.89 |
| ASYR | Coted'Ivoire | Both | 2021 | 118.31 | 166.32 | 79.08 |
| ASYR | Ghana | Both | 2021 | 120.38 | 170.77 | 78.71 |
| ASYR | Guinea | Both | 2021 | 108.50 | 151.30 | 72.38 |
| ASYR | Guinea-Bissau | Both | 2021 | 118.88 | 169.47 | 77.91 |
| ASYR | Mali | Both | 2021 | 135.36 | 192.58 | 89.34 |
| ASYR | Mauritania | Both | 2021 | 108.84 | 151.77 | 71.81 |
| ASYR | Liberia | Both | 2021 | 108.80 | 152.99 | 72.20 |
| ASYR | Niger | Both | 2021 | 140.25 | 197.96 | 91.85 |
| ASYR | Senegal | Both | 2021 | 122.44 | 172.37 | 81.13 |
| ASYR | Nigeria | Both | 2021 | 119.63 | 166.89 | 79.55 |
| ASYR | Sao Tome and Principe | Both | 2021 | 152.18 | 214.01 | 101.08 |
| ASYR | Sierra Leone | Both | 2021 | 107.45 | 150.79 | 71.01 |
| ASYR | Togo | Both | 2021 | 120.49 | 169.38 | 80.02 |
| ASYR | Bermuda | Both | 2021 | 56.76 | 82.44 | 36.63 |
| ASYR | American Samoa | Both | 2021 | 97.53 | 136.27 | 65.04 |
| ASYR | Cook Islands | Both | 2021 | 61.40 | 86.05 | 41.14 |
| ASYR | Greenland | Both | 2021 | 576.35 | 797.67 | 391.84 |
| ASYR | Guam | Both | 2021 | 63.68 | 89.02 | 42.30 |
| ASYR | Monaco | Both | 2021 | 172.39 | 246.37 | 113.80 |
| ASYR | Palau | Both | 2021 | 169.80 | 239.01 | 113.23 |
| ASYR | Nauru | Both | 2021 | 115.88 | 163.52 | 76.37 |
| ASYR | Puerto Rico | Both | 2021 | 83.70 | 120.44 | 55.36 |
| ASYR | Niue | Both | 2021 | 91.74 | 127.86 | 60.62 |
| ASYR | Saint Kitts and Nevis | Both | 2021 | 103.43 | 143.63 | 69.28 |
| ASYR | Northern Mariana Islands | Both | 2021 | 144.73 | 199.03 | 96.97 |
| ASYR | Tuvalu | Both | 2021 | 104.58 | 145.23 | 69.46 |
| ASYR | San Marino | Both | 2021 | 200.39 | 283.39 | 132.17 |
| ASYR | United States Virgin Islands | Both | 2021 | 94.41 | 132.97 | 63.04 |
| ASYR | Tokelau | Both | 2021 | 90.10 | 126.30 | 59.92 |
| ASYR | South Sudan | Both | 2021 | 85.03 | 121.09 | 55.00 |
| ASYR | Sudan | Both | 2021 | 88.63 | 123.20 | 60.50 |

Abbreviations: ASIR, age-standardized incidence rate; ASPR, age-standardized prevalence rate; ASYR, age-standardized years lived with disability rate; HFs, Hip fractures; UI, uncertainty interval.

**Table S4. The results of new cases, prevalence count, and YLDs for HFs (≥55 years) across 204 countries and territories**

| **measure** | **location** | **sex** | **year** | **value** | **95% UI_upper** | **95% UI_lower** |
| --- | --- | --- | --- | --- | --- | --- |
| new cases | Afghanistan | Both | 2021 | 5184 | 9386 | 3153 |
| new cases | Albania | Both | 2021 | 3703 | 5412 | 2561 |
| new cases | Algeria | Both | 2021 | 18215 | 24373 | 13276 |
| new cases | American Samoa | Both | 2021 | 27 | 36 | 19 |
| new cases | Andorra | Both | 2021 | 1020 | 1373 | 715 |
| new cases | Angola | Both | 2021 | 4739 | 6419 | 3445 |
| new cases | Antigua and Barbuda | Both | 2021 | 69 | 99 | 48 |
| new cases | Argentina | Both | 2021 | 58173 | 77207 | 42368 |
| new cases | Armenia | Both | 2021 | 2331 | 3132 | 1721 |
| new cases | Australia | Both | 2021 | 227650 | 299981 | 164330 |
| new cases | Austria | Both | 2021 | 72498 | 96743 | 51812 |
| new cases | Azerbaijan | Both | 2021 | 3190 | 4435 | 2297 |
| new cases | Bahamas | Both | 2021 | 225 | 302 | 164 |
| new cases | Bahrain | Both | 2021 | 262 | 350 | 192 |
| new cases | Bangladesh | Both | 2021 | 33926 | 45507 | 25043 |
| new cases | Barbados | Both | 2021 | 350 | 471 | 253 |
| new cases | Belarus | Both | 2021 | 16859 | 23030 | 12107 |
| new cases | Belgium | Both | 2021 | 127620 | 169913 | 90775 |
| new cases | Belize | Both | 2021 | 162 | 221 | 118 |
| new cases | Benin | Both | 2021 | 2666 | 3579 | 1941 |
| new cases | Bermuda | Both | 2021 | 123 | 166 | 88 |
| new cases | Bhutan | Both | 2021 | 818 | 1153 | 559 |
| new cases | Bolivia (Plurinational State of) | Both | 2021 | 5847 | 7929 | 4272 |
| new cases | Bosnia and Herzegovina | Both | 2021 | 6736 | 9422 | 4749 |
| new cases | Botswana | Both | 2021 | 537 | 718 | 395 |
| new cases | Brazil | Both | 2021 | 260747 | 373887 | 175921 |
| new cases | Brunei Darussalam | Both | 2021 | 373 | 498 | 271 |
| new cases | Bulgaria | Both | 2021 | 13224 | 18733 | 9481 |
| new cases | Burkina Faso | Both | 2021 | 5700 | 7583 | 4191 |
| new cases | Burundi | Both | 2021 | 1934 | 2582 | 1423 |
| new cases | Cabo Verde | Both | 2021 | 312 | 418 | 227 |
| new cases | Cambodia | Both | 2021 | 11823 | 16577 | 8195 |
| new cases | Cameroon | Both | 2021 | 6416 | 8669 | 4655 |
| new cases | Canada | Both | 2021 | 322623 | 430667 | 230559 |
| new cases | Central African Republic | Both | 2021 | 769 | 1018 | 574 |
| new cases | Chad | Both | 2021 | 2583 | 3446 | 1914 |
| new cases | Chile | Both | 2021 | 55800 | 74314 | 40117 |
| new cases | China | Both | 2021 | 2571815 | 3757528 | 1698408 |
| new cases | Colombia | Both | 2021 | 32209 | 43012 | 23778 |
| new cases | Comoros | Both | 2021 | 240 | 320 | 178 |
| new cases | Congo | Both | 2021 | 1078 | 1451 | 787 |
| new cases | Cook Islands | Both | 2021 | 15 | 21 | 11 |
| new cases | Costa Rica | Both | 2021 | 7440 | 10024 | 5309 |
| new cases | Coted'Ivoire | Both | 2021 | 5611 | 7482 | 4124 |
| new cases | Croatia | Both | 2021 | 43879 | 59875 | 30464 |
| new cases | Cuba | Both | 2021 | 61694 | 83410 | 43456 |
| new cases | Cyprus | Both | 2021 | 7457 | 10003 | 5326 |
| new cases | Czechia | Both | 2021 | 61533 | 84804 | 43142 |
| new cases | Democratic People's Republic of Korea | Both | 2021 | 11428 | 15073 | 8484 |
| new cases | Democratic Republic of the Congo | Both | 2021 | 14949 | 20120 | 10813 |
| new cases | Denmark | Both | 2021 | 42695 | 56609 | 30993 |
| new cases | Djibouti | Both | 2021 | 265 | 354 | 195 |
| new cases | Dominica | Both | 2021 | 37 | 50 | 27 |
| new cases | Dominican Republic | Both | 2021 | 5964 | 7983 | 4395 |
| new cases | Ecuador | Both | 2021 | 11676 | 15545 | 8620 |
| new cases | Egypt | Both | 2021 | 25252 | 34159 | 18319 |
| new cases | El Salvador | Both | 2021 | 6418 | 8558 | 4688 |
| new cases | Equatorial Guinea | Both | 2021 | 231 | 314 | 167 |
| new cases | Eritrea | Both | 2021 | 1231 | 1654 | 901 |
| new cases | Estonia | Both | 2021 | 2731 | 3712 | 1949 |
| new cases | Eswatini | Both | 2021 | 166 | 221 | 124 |
| new cases | Ethiopia | Both | 2021 | 25864 | 35815 | 18244 |
| new cases | Fiji | Both | 2021 | 240 | 323 | 176 |
| new cases | Finland | Both | 2021 | 62183 | 82990 | 44497 |
| new cases | France | Both | 2021 | 776627 | 1030231 | 565236 |
| new cases | Gabon | Both | 2021 | 565 | 766 | 408 |
| new cases | Gambia | Both | 2021 | 574 | 777 | 414 |
| new cases | Georgia | Both | 2021 | 4857 | 6590 | 3518 |
| new cases | Germany | Both | 2021 | 785785 | 1046967 | 565850 |
| new cases | Ghana | Both | 2021 | 8584 | 11667 | 6137 |
| new cases | Greece | Both | 2021 | 33193 | 44041 | 24016 |
| new cases | Greenland | Both | 2021 | 210 | 290 | 147 |
| new cases | Grenada | Both | 2021 | 73 | 101 | 52 |
| new cases | Guam | Both | 2021 | 115 | 155 | 83 |
| new cases | Guatemala | Both | 2021 | 9688 | 13132 | 7026 |
| new cases | Guinea | Both | 2021 | 2765 | 3686 | 2044 |
| new cases | Guinea-Bissau | Both | 2021 | 328 | 439 | 240 |
| new cases | Guyana | Both | 2021 | 451 | 610 | 330 |
| new cases | Haiti | Both | 2021 | 3131 | 4386 | 2235 |
| new cases | Honduras | Both | 2021 | 3675 | 4988 | 2660 |
| new cases | Hungary | Both | 2021 | 57522 | 78265 | 40652 |
| new cases | Iceland | Both | 2021 | 1706 | 2252 | 1238 |
| new cases | India | Both | 2021 | 1992877 | 2962186 | 1276024 |
| new cases | Indonesia | Both | 2021 | 150773 | 218287 | 100825 |
| new cases | Iran (Islamic Republic of) | Both | 2021 | 47537 | 65969 | 33041 |
| new cases | Iraq | Both | 2021 | 10575 | 14619 | 7639 |
| new cases | Ireland | Both | 2021 | 18987 | 25229 | 13610 |
| new cases | Israel | Both | 2021 | 21746 | 28575 | 15765 |
| new cases | Italy | Both | 2021 | 516100 | 734129 | 341645 |
| new cases | Jamaica | Both | 2021 | 1685 | 2271 | 1225 |
| new cases | Japan | Both | 2021 | 632202 | 905127 | 419427 |
| new cases | Jordan | Both | 2021 | 2594 | 3469 | 1901 |
| new cases | Kazakhstan | Both | 2021 | 10251 | 14052 | 7353 |
| new cases | Kenya | Both | 2021 | 13773 | 19061 | 9690 |
| new cases | Kiribati | Both | 2021 | 14 | 19 | 10 |
| new cases | Kuwait | Both | 2021 | 1093 | 1437 | 811 |
| new cases | Kyrgyzstan | Both | 2021 | 1639 | 2181 | 1213 |
| new cases | Lao People's Democratic Republic | Both | 2021 | 2033 | 2757 | 1474 |
| new cases | Latvia | Both | 2021 | 4758 | 6461 | 3385 |
| new cases | Lebanon | Both | 2021 | 3982 | 5419 | 2855 |
| new cases | Lesotho | Both | 2021 | 398 | 526 | 295 |
| new cases | Liberia | Both | 2021 | 914 | 1246 | 654 |
| new cases | Libya | Both | 2021 | 6205 | 10609 | 3767 |
| new cases | Lithuania | Both | 2021 | 8765 | 11900 | 6239 |
| new cases | Luxembourg | Both | 2021 | 4331 | 5732 | 3088 |
| new cases | Madagascar | Both | 2021 | 3337 | 4519 | 2456 |
| new cases | Malawi | Both | 2021 | 3266 | 4416 | 2387 |
| new cases | Malaysia | Both | 2021 | 19696 | 26832 | 14071 |
| new cases | Maldives | Both | 2021 | 224 | 309 | 156 |
| new cases | Mali | Both | 2021 | 4833 | 6615 | 3454 |
| new cases | Malta | Both | 2021 | 2642 | 3544 | 1882 |
| new cases | Marshall Islands | Both | 2021 | 12 | 16 | 9 |
| new cases | Mauritania | Both | 2021 | 1219 | 1632 | 885 |
| new cases | Mauritius | Both | 2021 | 680 | 911 | 496 |
| new cases | Mexico | Both | 2021 | 123855 | 176643 | 84255 |
| new cases | Micronesia (Federated States of) | Both | 2021 | 36 | 48 | 26 |
| new cases | Monaco | Both | 2021 | 221 | 299 | 157 |
| new cases | Mongolia | Both | 2021 | 969 | 1320 | 702 |
| new cases | Montenegro | Both | 2021 | 1350 | 1867 | 952 |
| new cases | Morocco | Both | 2021 | 25974 | 35899 | 18401 |
| new cases | Mozambique | Both | 2021 | 5226 | 6924 | 3863 |
| new cases | Myanmar | Both | 2021 | 36925 | 50813 | 26250 |
| new cases | Namibia | Both | 2021 | 477 | 633 | 353 |
| new cases | Nauru | Both | 2021 | 3 | 4 | 2 |
| new cases | Nepal | Both | 2021 | 22289 | 32159 | 15010 |
| new cases | Netherlands | Both | 2021 | 194044 | 255539 | 139194 |
| new cases | New Zealand | Both | 2021 | 34725 | 49229 | 23157 |
| new cases | Nicaragua | Both | 2021 | 4818 | 6532 | 3437 |
| new cases | Niger | Both | 2021 | 4430 | 6053 | 3190 |
| new cases | Nigeria | Both | 2021 | 49757 | 70456 | 34143 |
| new cases | Niue | Both | 2021 | 1 | 2 | 1 |
| new cases | North Macedonia | Both | 2021 | 4068 | 5701 | 2862 |
| new cases | Northern Mariana Islands | Both | 2021 | 42 | 57 | 30 |
| new cases | Norway | Both | 2021 | 59999 | 86774 | 39078 |
| new cases | Oman | Both | 2021 | 1776 | 2405 | 1274 |
| new cases | Pakistan | Both | 2021 | 39130 | 53489 | 27902 |
| new cases | Palau | Both | 2021 | 20 | 28 | 14 |
| new cases | Palestine | Both | 2021 | 1269 | 1724 | 906 |
| new cases | Panama | Both | 2021 | 2404 | 3226 | 1759 |
| new cases | Papua New Guinea | Both | 2021 | 3461 | 4877 | 2364 |
| new cases | Paraguay | Both | 2021 | 4133 | 5504 | 3019 |
| new cases | Peru | Both | 2021 | 17413 | 23728 | 12705 |
| new cases | Philippines | Both | 2021 | 37409 | 52793 | 25619 |
| new cases | Poland | Both | 2021 | 137598 | 200248 | 90632 |
| new cases | Portugal | Both | 2021 | 48929 | 65001 | 35147 |
| new cases | Puerto Rico | Both | 2021 | 8268 | 11239 | 5897 |
| new cases | Qatar | Both | 2021 | 400 | 537 | 289 |
| new cases | Republic of Korea | Both | 2021 | 271435 | 365854 | 193620 |
| new cases | Republic of Moldova | Both | 2021 | 3994 | 5382 | 2925 |
| new cases | Romania | Both | 2021 | 35869 | 49675 | 25756 |
| new cases | Russian Federation | Both | 2021 | 238033 | 333365 | 165932 |
| new cases | Rwanda | Both | 2021 | 2900 | 3871 | 2117 |
| new cases | Saint Kitts and Nevis | Both | 2021 | 45 | 62 | 33 |
| new cases | Saint Lucia | Both | 2021 | 132 | 179 | 97 |
| new cases | Saint Vincent and the Grenadines | Both | 2021 | 86 | 116 | 62 |
| new cases | Samoa | Both | 2021 | 81 | 109 | 58 |
| new cases | San Marino | Both | 2021 | 208 | 279 | 148 |
| new cases | Sao Tome and Principe | Both | 2021 | 85 | 118 | 60 |
| new cases | Saudi Arabia | Both | 2021 | 21886 | 30009 | 15369 |
| new cases | Senegal | Both | 2021 | 4263 | 5786 | 3079 |
| new cases | Serbia | Both | 2021 | 22945 | 31613 | 16163 |
| new cases | Seychelles | Both | 2021 | 49 | 66 | 36 |
| new cases | Sierra Leone | Both | 2021 | 1723 | 2335 | 1253 |
| new cases | Singapore | Both | 2021 | 8541 | 11423 | 6104 |
| new cases | Slovakia | Both | 2021 | 21939 | 30319 | 15295 |
| new cases | Slovenia | Both | 2021 | 21209 | 28926 | 14827 |
| new cases | Solomon Islands | Both | 2021 | 670 | 954 | 453 |
| new cases | Somalia | Both | 2021 | 2358 | 3162 | 1752 |
| new cases | South Africa | Both | 2021 | 14741 | 20119 | 10528 |
| new cases | South Sudan | Both | 2021 | 1470 | 1962 | 1089 |
| new cases | Spain | Both | 2021 | 221263 | 294671 | 159216 |
| new cases | Sri Lanka | Both | 2021 | 31589 | 43343 | 22266 |
| new cases | Sudan | Both | 2021 | 8119 | 10710 | 6014 |
| new cases | Suriname | Both | 2021 | 360 | 488 | 262 |
| new cases | Sweden | Both | 2021 | 97921 | 141940 | 63403 |
| new cases | Switzerland | Both | 2021 | 109077 | 143516 | 78428 |
| new cases | Syrian Arab Republic | Both | 2021 | 5713 | 7607 | 4198 |
| new cases | Taiwan (Province of China) | Both | 2021 | 28046 | 37841 | 20297 |
| new cases | Tajikistan | Both | 2021 | 1641 | 2247 | 1198 |
| new cases | Thailand | Both | 2021 | 74071 | 101604 | 52867 |
| new cases | Timor-Leste | Both | 2021 | 437 | 592 | 317 |
| new cases | Togo | Both | 2021 | 1780 | 2408 | 1299 |
| new cases | Tokelau | Both | 2021 | 1 | 1 | 1 |
| new cases | Tonga | Both | 2021 | 40 | 54 | 29 |
| new cases | Trinidad and Tobago | Both | 2021 | 809 | 1080 | 596 |
| new cases | Tunisia | Both | 2021 | 7855 | 10537 | 5669 |
| new cases | Turkey | Both | 2021 | 84695 | 116502 | 58861 |
| new cases | Turkmenistan | Both | 2021 | 1067 | 1521 | 755 |
| new cases | Tuvalu | Both | 2021 | 5 | 7 | 4 |
| new cases | Uganda | Both | 2021 | 6939 | 9252 | 5088 |
| new cases | Ukraine | Both | 2021 | 54935 | 76953 | 38199 |
| new cases | United Arab Emirates | Both | 2021 | 1534 | 2083 | 1110 |
| new cases | United Kingdom | Both | 2021 | 388558 | 548180 | 260177 |
| new cases | United Republic of Tanzania | Both | 2021 | 12483 | 16880 | 9098 |
| new cases | United States Virgin Islands | Both | 2021 | 159 | 216 | 115 |
| new cases | United States of America | Both | 2021 | 1748113 | 2555622 | 1124470 |
| new cases | Uruguay | Both | 2021 | 10501 | 13906 | 7609 |
| new cases | Uzbekistan | Both | 2021 | 9135 | 12224 | 6705 |
| new cases | Vanuatu | Both | 2021 | 57 | 77 | 41 |
| new cases | Venezuela (Bolivarian Republic of) | Both | 2021 | 21497 | 28755 | 15711 |
| new cases | Viet Nam | Both | 2021 | 139099 | 194973 | 95640 |
| new cases | Yemen | Both | 2021 | 7540 | 10791 | 5380 |
| new cases | Zambia | Both | 2021 | 3566 | 4784 | 2607 |
| new cases | Zimbabwe | Both | 2021 | 2601 | 3476 | 1912 |
| prevalence count | Afghanistan | Both | 2021 | 8772 | 18104 | 4780 |
| prevalence count | Albania | Both | 2021 | 5810 | 6912 | 4899 |
| prevalence count | Algeria | Both | 2021 | 33609 | 38967 | 29086 |
| prevalence count | American Samoa | Both | 2021 | 37 | 44 | 30 |
| prevalence count | Andorra | Both | 2021 | 2111 | 2488 | 1775 |
| prevalence count | Angola | Both | 2021 | 7735 | 10299 | 6122 |
| prevalence count | Antigua and Barbuda | Both | 2021 | 90 | 108 | 76 |
| prevalence count | Argentina | Both | 2021 | 146468 | 174336 | 123104 |
| prevalence count | Armenia | Both | 2021 | 4105 | 5133 | 3379 |
| prevalence count | Australia | Both | 2021 | 458679 | 542552 | 385318 |
| prevalence count | Austria | Both | 2021 | 168112 | 199960 | 139654 |
| prevalence count | Azerbaijan | Both | 2021 | 4944 | 5768 | 4233 |
| prevalence count | Bahamas | Both | 2021 | 377 | 452 | 314 |
| prevalence count | Bahrain | Both | 2021 | 432 | 502 | 373 |
| prevalence count | Bangladesh | Both | 2021 | 58112 | 69185 | 49171 |
| prevalence count | Barbados | Both | 2021 | 518 | 626 | 428 |
| prevalence count | Belarus | Both | 2021 | 30047 | 36248 | 24761 |
| prevalence count | Belgium | Both | 2021 | 296073 | 350482 | 247523 |
| prevalence count | Belize | Both | 2021 | 257 | 302 | 220 |
| prevalence count | Benin | Both | 2021 | 3673 | 4421 | 3055 |
| prevalence count | Bermuda | Both | 2021 | 195 | 236 | 161 |
| prevalence count | Bhutan | Both | 2021 | 1205 | 1524 | 949 |
| prevalence count | Bolivia (Plurinational State of) | Both | 2021 | 8797 | 10441 | 7430 |
| prevalence count | Bosnia and Herzegovina | Both | 2021 | 10631 | 12880 | 8699 |
| prevalence count | Botswana | Both | 2021 | 725 | 874 | 604 |
| prevalence count | Brazil | Both | 2021 | 483499 | 597151 | 394079 |
| prevalence count | Brunei Darussalam | Both | 2021 | 931 | 1103 | 781 |
| prevalence count | Bulgaria | Both | 2021 | 20418 | 24667 | 17011 |
| prevalence count | Burkina Faso | Both | 2021 | 7175 | 8651 | 5992 |
| prevalence count | Burundi | Both | 2021 | 2868 | 3667 | 2330 |
| prevalence count | Cabo Verde | Both | 2021 | 447 | 540 | 368 |
| prevalence count | Cambodia | Both | 2021 | 20060 | 29606 | 14868 |
| prevalence count | Cameroon | Both | 2021 | 8789 | 10761 | 7176 |
| prevalence count | Canada | Both | 2021 | 702036 | 835103 | 585032 |
| prevalence count | Central African Republic | Both | 2021 | 892 | 1062 | 750 |
| prevalence count | Chad | Both | 2021 | 3425 | 4256 | 2806 |
| prevalence count | Chile | Both | 2021 | 130751 | 156389 | 107995 |
| prevalence count | China | Both | 2021 | 4224272 | 5310803 | 3374913 |
| prevalence count | Colombia | Both | 2021 | 64450 | 76437 | 54378 |
| prevalence count | Comoros | Both | 2021 | 323 | 384 | 273 |
| prevalence count | Congo | Both | 2021 | 1464 | 1773 | 1218 |
| prevalence count | Cook Islands | Both | 2021 | 25 | 30 | 21 |
| prevalence count | Costa Rica | Both | 2021 | 12645 | 15183 | 10418 |
| prevalence count | Coted'Ivoire | Both | 2021 | 7213 | 8665 | 6013 |
| prevalence count | Croatia | Both | 2021 | 62542 | 76463 | 50730 |
| prevalence count | Cuba | Both | 2021 | 92941 | 112748 | 75431 |
| prevalence count | Cyprus | Both | 2021 | 14740 | 17617 | 12220 |
| prevalence count | Czechia | Both | 2021 | 96117 | 117881 | 77998 |
| prevalence count | Democratic People's Republic of Korea | Both | 2021 | 22013 | 25300 | 19106 |
| prevalence count | Democratic Republic of the Congo | Both | 2021 | 21640 | 25724 | 18116 |
| prevalence count | Denmark | Both | 2021 | 86133 | 102212 | 72084 |
| prevalence count | Djibouti | Both | 2021 | 335 | 399 | 283 |
| prevalence count | Dominica | Both | 2021 | 58 | 69 | 49 |
| prevalence count | Dominican Republic | Both | 2021 | 9092 | 10712 | 7730 |
| prevalence count | Ecuador | Both | 2021 | 19712 | 23197 | 16794 |
| prevalence count | Egypt | Both | 2021 | 40535 | 47194 | 35097 |
| prevalence count | El Salvador | Both | 2021 | 12412 | 16175 | 9840 |
| prevalence count | Equatorial Guinea | Both | 2021 | 315 | 383 | 258 |
| prevalence count | Eritrea | Both | 2021 | 2351 | 3759 | 1563 |
| prevalence count | Estonia | Both | 2021 | 5272 | 6353 | 4362 |
| prevalence count | Eswatini | Both | 2021 | 212 | 251 | 180 |
| prevalence count | Ethiopia | Both | 2021 | 33170 | 41355 | 27044 |
| prevalence count | Fiji | Both | 2021 | 331 | 396 | 278 |
| prevalence count | Finland | Both | 2021 | 147717 | 174893 | 123170 |
| prevalence count | France | Both | 2021 | 1699030 | 1995223 | 1429835 |
| prevalence count | Gabon | Both | 2021 | 763 | 925 | 630 |
| prevalence count | Gambia | Both | 2021 | 777 | 966 | 629 |
| prevalence count | Georgia | Both | 2021 | 8699 | 10434 | 7228 |
| prevalence count | Germany | Both | 2021 | 1733823 | 2052109 | 1437257 |
| prevalence count | Ghana | Both | 2021 | 11855 | 14540 | 9733 |
| prevalence count | Greece | Both | 2021 | 91147 | 107691 | 76366 |
| prevalence count | Greenland | Both | 2021 | 475 | 583 | 384 |
| prevalence count | Grenada | Both | 2021 | 103 | 130 | 84 |
| prevalence count | Guam | Both | 2021 | 185 | 224 | 152 |
| prevalence count | Guatemala | Both | 2021 | 16664 | 21111 | 13539 |
| prevalence count | Guinea | Both | 2021 | 3565 | 4268 | 2981 |
| prevalence count | Guinea-Bissau | Both | 2021 | 400 | 481 | 333 |
| prevalence count | Guyana | Both | 2021 | 624 | 749 | 519 |
| prevalence count | Haiti | Both | 2021 | 5339 | 8594 | 3789 |
| prevalence count | Honduras | Both | 2021 | 5455 | 6368 | 4692 |
| prevalence count | Hungary | Both | 2021 | 89417 | 111620 | 71457 |
| prevalence count | Iceland | Both | 2021 | 3978 | 4715 | 3317 |
| prevalence count | India | Both | 2021 | 2973852 | 3877759 | 2294064 |
| prevalence count | Indonesia | Both | 2021 | 225722 | 286575 | 179802 |
| prevalence count | Iran (Islamic Republic of) | Both | 2021 | 90809 | 113024 | 75034 |
| prevalence count | Iraq | Both | 2021 | 27914 | 44785 | 18732 |
| prevalence count | Ireland | Both | 2021 | 46182 | 54859 | 38389 |
| prevalence count | Israel | Both | 2021 | 52016 | 60939 | 43793 |
| prevalence count | Italy | Both | 2021 | 1213604 | 1473089 | 992326 |
| prevalence count | Jamaica | Both | 2021 | 2750 | 3283 | 2309 |
| prevalence count | Japan | Both | 2021 | 1710876 | 2068867 | 1401637 |
| prevalence count | Jordan | Both | 2021 | 4517 | 5344 | 3833 |
| prevalence count | Kazakhstan | Both | 2021 | 16878 | 19855 | 14372 |
| prevalence count | Kenya | Both | 2021 | 17148 | 21178 | 14015 |
| prevalence count | Kiribati | Both | 2021 | 20 | 24 | 17 |
| prevalence count | Kuwait | Both | 2021 | 2044 | 2397 | 1740 |
| prevalence count | Kyrgyzstan | Both | 2021 | 2954 | 3382 | 2583 |
| prevalence count | Lao People's Democratic Republic | Both | 2021 | 3065 | 3670 | 2565 |
| prevalence count | Latvia | Both | 2021 | 8483 | 10207 | 6977 |
| prevalence count | Lebanon | Both | 2021 | 8051 | 12236 | 5842 |
| prevalence count | Lesotho | Both | 2021 | 447 | 534 | 375 |
| prevalence count | Liberia | Both | 2021 | 1326 | 1659 | 1076 |
| prevalence count | Libya | Both | 2021 | 6676 | 8058 | 5616 |
| prevalence count | Lithuania | Both | 2021 | 16465 | 19778 | 13534 |
| prevalence count | Luxembourg | Both | 2021 | 9621 | 11362 | 8071 |
| prevalence count | Madagascar | Both | 2021 | 4373 | 5193 | 3701 |
| prevalence count | Malawi | Both | 2021 | 4237 | 5216 | 3474 |
| prevalence count | Malaysia | Both | 2021 | 30224 | 36954 | 24749 |
| prevalence count | Maldives | Both | 2021 | 344 | 421 | 278 |
| prevalence count | Mali | Both | 2021 | 6612 | 8101 | 5406 |
| prevalence count | Malta | Both | 2021 | 6459 | 7720 | 5387 |
| prevalence count | Marshall Islands | Both | 2021 | 17 | 20 | 14 |
| prevalence count | Mauritania | Both | 2021 | 1742 | 2103 | 1452 |
| prevalence count | Mauritius | Both | 2021 | 1188 | 1422 | 992 |
| prevalence count | Mexico | Both | 2021 | 207181 | 257416 | 168875 |
| prevalence count | Micronesia (Federated States of) | Both | 2021 | 47 | 56 | 39 |
| prevalence count | Monaco | Both | 2021 | 495 | 596 | 407 |
| prevalence count | Mongolia | Both | 2021 | 1735 | 2023 | 1488 |
| prevalence count | Montenegro | Both | 2021 | 2039 | 2521 | 1664 |
| prevalence count | Morocco | Both | 2021 | 40435 | 48987 | 33481 |
| prevalence count | Mozambique | Both | 2021 | 6757 | 9011 | 5302 |
| prevalence count | Myanmar | Both | 2021 | 60934 | 75578 | 49436 |
| prevalence count | Namibia | Both | 2021 | 717 | 867 | 601 |
| prevalence count | Nauru | Both | 2021 | 4 | 4 | 3 |
| prevalence count | Nepal | Both | 2021 | 35790 | 45226 | 28389 |
| prevalence count | Netherlands | Both | 2021 | 370717 | 440272 | 308314 |
| prevalence count | New Zealand | Both | 2021 | 71385 | 86478 | 58496 |
| prevalence count | Nicaragua | Both | 2021 | 9213 | 12621 | 7035 |
| prevalence count | Niger | Both | 2021 | 5935 | 7291 | 4860 |
| prevalence count | Nigeria | Both | 2021 | 70130 | 87967 | 56499 |
| prevalence count | Niue | Both | 2021 | 2 | 2 | 2 |
| prevalence count | North Macedonia | Both | 2021 | 5340 | 6525 | 4364 |
| prevalence count | Northern Mariana Islands | Both | 2021 | 61 | 73 | 51 |
| prevalence count | Norway | Both | 2021 | 113813 | 138040 | 93120 |
| prevalence count | Oman | Both | 2021 | 2380 | 2875 | 1977 |
| prevalence count | Pakistan | Both | 2021 | 52792 | 63758 | 43957 |
| prevalence count | Palau | Both | 2021 | 28 | 34 | 23 |
| prevalence count | Palestine | Both | 2021 | 2716 | 4096 | 1963 |
| prevalence count | Panama | Both | 2021 | 4520 | 5302 | 3834 |
| prevalence count | Papua New Guinea | Both | 2021 | 5337 | 6510 | 4374 |
| prevalence count | Paraguay | Both | 2021 | 7269 | 8624 | 6108 |
| prevalence count | Peru | Both | 2021 | 33291 | 39864 | 27977 |
| prevalence count | Philippines | Both | 2021 | 62232 | 78014 | 50601 |
| prevalence count | Poland | Both | 2021 | 230125 | 287483 | 184532 |
| prevalence count | Portugal | Both | 2021 | 128951 | 153595 | 107110 |
| prevalence count | Puerto Rico | Both | 2021 | 15957 | 19437 | 13094 |
| prevalence count | Qatar | Both | 2021 | 653 | 759 | 560 |
| prevalence count | Republic of Korea | Both | 2021 | 687320 | 821956 | 571544 |
| prevalence count | Republic of Moldova | Both | 2021 | 7171 | 8591 | 5985 |
| prevalence count | Romania | Both | 2021 | 65041 | 78398 | 53892 |
| prevalence count | Russian Federation | Both | 2021 | 419929 | 513225 | 347246 |
| prevalence count | Rwanda | Both | 2021 | 5334 | 7886 | 3923 |
| prevalence count | Saint Kitts and Nevis | Both | 2021 | 60 | 72 | 50 |
| prevalence count | Saint Lucia | Both | 2021 | 202 | 237 | 172 |
| prevalence count | Saint Vincent and the Grenadines | Both | 2021 | 123 | 146 | 104 |
| prevalence count | Samoa | Both | 2021 | 127 | 154 | 105 |
| prevalence count | San Marino | Both | 2021 | 483 | 576 | 399 |
| prevalence count | Sao Tome and Principe | Both | 2021 | 119 | 148 | 96 |
| prevalence count | Saudi Arabia | Both | 2021 | 37660 | 44887 | 31296 |
| prevalence count | Senegal | Both | 2021 | 5997 | 7352 | 4912 |
| prevalence count | Serbia | Both | 2021 | 32524 | 39954 | 26487 |
| prevalence count | Seychelles | Both | 2021 | 78 | 94 | 66 |
| prevalence count | Sierra Leone | Both | 2021 | 2402 | 2911 | 1983 |
| prevalence count | Singapore | Both | 2021 | 26612 | 31726 | 22244 |
| prevalence count | Slovakia | Both | 2021 | 35286 | 43192 | 28610 |
| prevalence count | Slovenia | Both | 2021 | 34572 | 41633 | 28298 |
| prevalence count | Solomon Islands | Both | 2021 | 478 | 600 | 379 |
| prevalence count | Somalia | Both | 2021 | 2588 | 3294 | 2150 |
| prevalence count | South Africa | Both | 2021 | 21385 | 25109 | 18276 |
| prevalence count | South Sudan | Both | 2021 | 1800 | 2277 | 1479 |
| prevalence count | Spain | Both | 2021 | 581267 | 691719 | 484631 |
| prevalence count | Sri Lanka | Both | 2021 | 53282 | 66122 | 42926 |
| prevalence count | Sudan | Both | 2021 | 13670 | 16198 | 11726 |
| prevalence count | Suriname | Both | 2021 | 590 | 710 | 497 |
| prevalence count | Sweden | Both | 2021 | 202369 | 247704 | 163717 |
| prevalence count | Switzerland | Both | 2021 | 227164 | 266531 | 191980 |
| prevalence count | Syrian Arab Republic | Both | 2021 | 11616 | 16548 | 8806 |
| prevalence count | Taiwan (Province of China) | Both | 2021 | 55546 | 66959 | 45788 |
| prevalence count | Tajikistan | Both | 2021 | 2810 | 3317 | 2418 |
| prevalence count | Thailand | Both | 2021 | 149585 | 180703 | 124939 |
| prevalence count | Timor-Leste | Both | 2021 | 889 | 1316 | 635 |
| prevalence count | Togo | Both | 2021 | 2428 | 2926 | 2026 |
| prevalence count | Tokelau | Both | 2021 | 1 | 2 | 1 |
| prevalence count | Tonga | Both | 2021 | 62 | 74 | 52 |
| prevalence count | Trinidad and Tobago | Both | 2021 | 1359 | 1596 | 1157 |
| prevalence count | Tunisia | Both | 2021 | 13613 | 16080 | 11546 |
| prevalence count | Turkey | Both | 2021 | 138456 | 169085 | 113211 |
| prevalence count | Turkmenistan | Both | 2021 | 1682 | 1962 | 1445 |
| prevalence count | Tuvalu | Both | 2021 | 8 | 9 | 6 |
| prevalence count | Uganda | Both | 2021 | 10178 | 13035 | 8178 |
| prevalence count | Ukraine | Both | 2021 | 107798 | 131948 | 89513 |
| prevalence count | United Arab Emirates | Both | 2021 | 3126 | 3501 | 2774 |
| prevalence count | United Kingdom | Both | 2021 | 873202 | 1068350 | 709536 |
| prevalence count | United Republic of Tanzania | Both | 2021 | 17058 | 20792 | 14069 |
| prevalence count | United States Virgin Islands | Both | 2021 | 221 | 267 | 184 |
| prevalence count | United States of America | Both | 2021 | 4636189 | 5790029 | 3685198 |
| prevalence count | Uruguay | Both | 2021 | 24149 | 28701 | 20147 |
| prevalence count | Uzbekistan | Both | 2021 | 12558 | 14541 | 10847 |
| prevalence count | Vanuatu | Both | 2021 | 80 | 95 | 68 |
| prevalence count | Venezuela (Bolivarian Republic of) | Both | 2021 | 36853 | 43610 | 30873 |
| prevalence count | Viet Nam | Both | 2021 | 209155 | 259096 | 168562 |
| prevalence count | Yemen | Both | 2021 | 11108 | 13373 | 9456 |
| prevalence count | Zambia | Both | 2021 | 4303 | 5243 | 3549 |
| prevalence count | Zimbabwe | Both | 2021 | 3115 | 3733 | 2595 |
| YLDs | Afghanistan | Both | 2021 | 1982 | 3865 | 951 |
| YLDs | Albania | Both | 2021 | 539 | 763 | 356 |
| YLDs | Algeria | Both | 2021 | 4118 | 5677 | 2808 |
| YLDs | American Samoa | Both | 2021 | 6 | 9 | 4 |
| YLDs | Andorra | Both | 2021 | 149 | 213 | 100 |
| YLDs | Angola | Both | 2021 | 1733 | 2579 | 1103 |
| YLDs | Antigua and Barbuda | Both | 2021 | 12 | 17 | 8 |
| YLDs | Argentina | Both | 2021 | 17454 | 23705 | 12085 |
| YLDs | Armenia | Both | 2021 | 464 | 658 | 309 |
| YLDs | Australia | Both | 2021 | 32722 | 46311 | 22028 |
| YLDs | Austria | Both | 2021 | 11609 | 16509 | 7702 |
| YLDs | Azerbaijan | Both | 2021 | 739 | 1034 | 493 |
| YLDs | Bahamas | Both | 2021 | 56 | 78 | 37 |
| YLDs | Bahrain | Both | 2021 | 40 | 57 | 26 |
| YLDs | Bangladesh | Both | 2021 | 10048 | 14241 | 6681 |
| YLDs | Barbados | Both | 2021 | 65 | 92 | 43 |
| YLDs | Belarus | Both | 2021 | 2389 | 3420 | 1562 |
| YLDs | Belgium | Both | 2021 | 20371 | 28811 | 13634 |
| YLDs | Belize | Both | 2021 | 42 | 58 | 28 |
| YLDs | Benin | Both | 2021 | 808 | 1146 | 538 |
| YLDs | Bermuda | Both | 2021 | 15 | 22 | 10 |
| YLDs | Bhutan | Both | 2021 | 216 | 306 | 142 |
| YLDs | Bolivia (Plurinational State of) | Both | 2021 | 1678 | 2341 | 1127 |
| YLDs | Bosnia and Herzegovina | Both | 2021 | 980 | 1391 | 654 |
| YLDs | Botswana | Both | 2021 | 145 | 208 | 95 |
| YLDs | Brazil | Both | 2021 | 67984 | 94003 | 46588 |
| YLDs | Brunei Darussalam | Both | 2021 | 118 | 161 | 82 |
| YLDs | Bulgaria | Both | 2021 | 2215 | 3096 | 1483 |
| YLDs | Burkina Faso | Both | 2021 | 1669 | 2338 | 1113 |
| YLDs | Burundi | Both | 2021 | 710 | 1035 | 458 |
| YLDs | Cabo Verde | Both | 2021 | 70 | 98 | 46 |
| YLDs | Cambodia | Both | 2021 | 3935 | 6224 | 2457 |
| YLDs | Cameroon | Both | 2021 | 1881 | 2665 | 1240 |
| YLDs | Canada | Both | 2021 | 49239 | 69839 | 32901 |
| YLDs | Central African Republic | Both | 2021 | 244 | 347 | 158 |
| YLDs | Chad | Both | 2021 | 841 | 1189 | 551 |
| YLDs | Chile | Both | 2021 | 10041 | 14159 | 6701 |
| YLDs | China | Both | 2021 | 359649 | 520885 | 233955 |
| YLDs | Colombia | Both | 2021 | 7438 | 10214 | 5043 |
| YLDs | Comoros | Both | 2021 | 71 | 99 | 48 |
| YLDs | Congo | Both | 2021 | 315 | 452 | 208 |
| YLDs | Cook Islands | Both | 2021 | 3 | 4 | 2 |
| YLDs | Costa Rica | Both | 2021 | 1292 | 1790 | 872 |
| YLDs | Coted'Ivoire | Both | 2021 | 1535 | 2167 | 1019 |
| YLDs | Croatia | Both | 2021 | 4974 | 7248 | 3175 |
| YLDs | Cuba | Both | 2021 | 9286 | 13075 | 6079 |
| YLDs | Cyprus | Both | 2021 | 1070 | 1535 | 709 |
| YLDs | Czechia | Both | 2021 | 7415 | 10790 | 4828 |
| YLDs | Democratic People's Republic of Korea | Both | 2021 | 3334 | 4631 | 2242 |
| YLDs | Democratic Republic of the Congo | Both | 2021 | 4987 | 7000 | 3325 |
| YLDs | Denmark | Both | 2021 | 6206 | 8841 | 4162 |
| YLDs | Djibouti | Both | 2021 | 73 | 104 | 48 |
| YLDs | Dominica | Both | 2021 | 10 | 14 | 7 |
| YLDs | Dominican Republic | Both | 2021 | 1557 | 2155 | 1049 |
| YLDs | Ecuador | Both | 2021 | 2899 | 3980 | 1984 |
| YLDs | Egypt | Both | 2021 | 6140 | 8469 | 4130 |
| YLDs | El Salvador | Both | 2021 | 1683 | 2414 | 1110 |
| YLDs | Equatorial Guinea | Both | 2021 | 57 | 81 | 38 |
| YLDs | Eritrea | Both | 2021 | 573 | 990 | 319 |
| YLDs | Estonia | Both | 2021 | 385 | 554 | 252 |
| YLDs | Eswatini | Both | 2021 | 46 | 65 | 30 |
| YLDs | Ethiopia | Both | 2021 | 7086 | 9924 | 4716 |
| YLDs | Fiji | Both | 2021 | 65 | 91 | 42 |
| YLDs | Finland | Both | 2021 | 10119 | 14343 | 6715 |
| YLDs | France | Both | 2021 | 118454 | 167468 | 79334 |
| YLDs | Gabon | Both | 2021 | 147 | 208 | 98 |
| YLDs | Gambia | Both | 2021 | 164 | 233 | 108 |
| YLDs | Georgia | Both | 2021 | 1153 | 1599 | 780 |
| YLDs | Germany | Both | 2021 | 120855 | 171410 | 80620 |
| YLDs | Ghana | Both | 2021 | 2446 | 3488 | 1592 |
| YLDs | Greece | Both | 2021 | 6084 | 8593 | 4027 |
| YLDs | Greenland | Both | 2021 | 54 | 75 | 36 |
| YLDs | Grenada | Both | 2021 | 16 | 23 | 11 |
| YLDs | Guam | Both | 2021 | 24 | 34 | 16 |
| YLDs | Guatemala | Both | 2021 | 2981 | 4275 | 1982 |
| YLDs | Guinea | Both | 2021 | 842 | 1178 | 560 |
| YLDs | Guinea-Bissau | Both | 2021 | 99 | 142 | 65 |
| YLDs | Guyana | Both | 2021 | 125 | 174 | 83 |
| YLDs | Haiti | Both | 2021 | 1332 | 2450 | 784 |
| YLDs | Honduras | Both | 2021 | 1072 | 1491 | 727 |
| YLDs | Hungary | Both | 2021 | 6939 | 10079 | 4455 |
| YLDs | Iceland | Both | 2021 | 276 | 388 | 185 |
| YLDs | India | Both | 2021 | 519727 | 736330 | 341790 |
| YLDs | Indonesia | Both | 2021 | 41751 | 58922 | 27865 |
| YLDs | Iran (Islamic Republic of) | Both | 2021 | 8878 | 12417 | 6038 |
| YLDs | Iraq | Both | 2021 | 3400 | 5579 | 2041 |
| YLDs | Ireland | Both | 2021 | 3175 | 4500 | 2099 |
| YLDs | Israel | Both | 2021 | 3575 | 5057 | 2394 |
| YLDs | Italy | Both | 2021 | 83215 | 119387 | 54420 |
| YLDs | Jamaica | Both | 2021 | 388 | 538 | 262 |
| YLDs | Japan | Both | 2021 | 114220 | 163910 | 74553 |
| YLDs | Jordan | Both | 2021 | 471 | 657 | 314 |
| YLDs | Kazakhstan | Both | 2021 | 2127 | 2918 | 1439 |
| YLDs | Kenya | Both | 2021 | 3512 | 4872 | 2366 |
| YLDs | Kiribati | Both | 2021 | 5 | 7 | 3 |
| YLDs | Kuwait | Both | 2021 | 151 | 217 | 99 |
| YLDs | Kyrgyzstan | Both | 2021 | 429 | 591 | 289 |
| YLDs | Lao People's Democratic Republic | Both | 2021 | 663 | 925 | 434 |
| YLDs | Latvia | Both | 2021 | 747 | 1062 | 501 |
| YLDs | Lebanon | Both | 2021 | 653 | 1047 | 402 |
| YLDs | Lesotho | Both | 2021 | 106 | 152 | 69 |
| YLDs | Liberia | Both | 2021 | 276 | 392 | 182 |
| YLDs | Libya | Both | 2021 | 889 | 1267 | 580 |
| YLDs | Lithuania | Both | 2021 | 1486 | 2078 | 981 |
| YLDs | Luxembourg | Both | 2021 | 673 | 959 | 450 |
| YLDs | Madagascar | Both | 2021 | 1029 | 1475 | 663 |
| YLDs | Malawi | Both | 2021 | 958 | 1374 | 629 |
| YLDs | Malaysia | Both | 2021 | 4140 | 5807 | 2763 |
| YLDs | Maldives | Both | 2021 | 41 | 58 | 27 |
| YLDs | Mali | Both | 2021 | 1494 | 2134 | 985 |
| YLDs | Malta | Both | 2021 | 443 | 633 | 294 |
| YLDs | Marshall Islands | Both | 2021 | 4 | 5 | 2 |
| YLDs | Mauritania | Both | 2021 | 329 | 459 | 217 |
| YLDs | Mauritius | Both | 2021 | 158 | 224 | 106 |
| YLDs | Mexico | Both | 2021 | 29880 | 41316 | 20346 |
| YLDs | Micronesia (Federated States of) | Both | 2021 | 10 | 14 | 6 |
| YLDs | Monaco | Both | 2021 | 35 | 50 | 23 |
| YLDs | Mongolia | Both | 2021 | 296 | 411 | 198 |
| YLDs | Montenegro | Both | 2021 | 161 | 236 | 105 |
| YLDs | Morocco | Both | 2021 | 6376 | 8860 | 4296 |
| YLDs | Mozambique | Both | 2021 | 1624 | 2399 | 1045 |
| YLDs | Myanmar | Both | 2021 | 11925 | 16772 | 7934 |
| YLDs | Namibia | Both | 2021 | 139 | 196 | 91 |
| YLDs | Nauru | Both | 2021 | 1 | 1 | 0 |
| YLDs | Nepal | Both | 2021 | 6789 | 9570 | 4475 |
| YLDs | Netherlands | Both | 2021 | 26925 | 38329 | 18096 |
| YLDs | New Zealand | Both | 2021 | 5117 | 7374 | 3334 |
| YLDs | Nicaragua | Both | 2021 | 1348 | 2062 | 871 |
| YLDs | Niger | Both | 2021 | 1421 | 2016 | 926 |
| YLDs | Nigeria | Both | 2021 | 13499 | 18819 | 9006 |
| YLDs | Niue | Both | 2021 | 0 | 0 | 0 |
| YLDs | North Macedonia | Both | 2021 | 550 | 789 | 363 |
| YLDs | Northern Mariana Islands | Both | 2021 | 8 | 12 | 6 |
| YLDs | Norway | Both | 2021 | 8241 | 11949 | 5368 |
| YLDs | Oman | Both | 2021 | 240 | 345 | 159 |
| YLDs | Pakistan | Both | 2021 | 11104 | 15516 | 7405 |
| YLDs | Palau | Both | 2021 | 4 | 6 | 3 |
| YLDs | Palestine | Both | 2021 | 335 | 523 | 207 |
| YLDs | Panama | Both | 2021 | 537 | 734 | 365 |
| YLDs | Papua New Guinea | Both | 2021 | 1183 | 1662 | 784 |
| YLDs | Paraguay | Both | 2021 | 1083 | 1495 | 732 |
| YLDs | Peru | Both | 2021 | 4055 | 5657 | 2751 |
| YLDs | Philippines | Both | 2021 | 11467 | 15955 | 7766 |
| YLDs | Poland | Both | 2021 | 18069 | 26281 | 11664 |
| YLDs | Portugal | Both | 2021 | 8675 | 12307 | 5832 |
| YLDs | Puerto Rico | Both | 2021 | 1263 | 1814 | 836 |
| YLDs | Qatar | Both | 2021 | 50 | 73 | 33 |
| YLDs | Republic of Korea | Both | 2021 | 46714 | 66399 | 30921 |
| YLDs | Republic of Moldova | Both | 2021 | 792 | 1102 | 532 |
| YLDs | Romania | Both | 2021 | 5737 | 8137 | 3811 |
| YLDs | Russian Federation | Both | 2021 | 36798 | 51908 | 24833 |
| YLDs | Rwanda | Both | 2021 | 1178 | 1870 | 717 |
| YLDs | Saint Kitts and Nevis | Both | 2021 | 9 | 13 | 6 |
| YLDs | Saint Lucia | Both | 2021 | 30 | 42 | 20 |
| YLDs | Saint Vincent and the Grenadines | Both | 2021 | 21 | 29 | 14 |
| YLDs | Samoa | Both | 2021 | 22 | 31 | 15 |
| YLDs | San Marino | Both | 2021 | 33 | 47 | 22 |
| YLDs | Sao Tome and Principe | Both | 2021 | 22 | 31 | 15 |
| YLDs | Saudi Arabia | Both | 2021 | 3976 | 5524 | 2685 |
| YLDs | Senegal | Both | 2021 | 1268 | 1793 | 838 |
| YLDs | Serbia | Both | 2021 | 2700 | 3948 | 1743 |
| YLDs | Seychelles | Both | 2021 | 11 | 16 | 8 |
| YLDs | Sierra Leone | Both | 2021 | 537 | 758 | 353 |
| YLDs | Singapore | Both | 2021 | 1752 | 2502 | 1166 |
| YLDs | Slovakia | Both | 2021 | 2719 | 3919 | 1761 |
| YLDs | Slovenia | Both | 2021 | 2621 | 3778 | 1707 |
| YLDs | Solomon Islands | Both | 2021 | 107 | 152 | 70 |
| YLDs | Somalia | Both | 2021 | 693 | 1002 | 446 |
| YLDs | South Africa | Both | 2021 | 3745 | 5088 | 2568 |
| YLDs | South Sudan | Both | 2021 | 409 | 593 | 260 |
| YLDs | Spain | Both | 2021 | 38985 | 54841 | 26024 |
| YLDs | Sri Lanka | Both | 2021 | 6159 | 8592 | 4154 |
| YLDs | Sudan | Both | 2021 | 2437 | 3405 | 1662 |
| YLDs | Suriname | Both | 2021 | 107 | 147 | 72 |
| YLDs | Sweden | Both | 2021 | 14349 | 20815 | 9319 |
| YLDs | Switzerland | Both | 2021 | 16034 | 22599 | 10766 |
| YLDs | Syrian Arab Republic | Both | 2021 | 1370 | 2088 | 883 |
| YLDs | Taiwan (Province of China) | Both | 2021 | 4041 | 5800 | 2628 |
| YLDs | Tajikistan | Both | 2021 | 535 | 752 | 351 |
| YLDs | Thailand | Both | 2021 | 15904 | 22088 | 10673 |
| YLDs | Timor-Leste | Both | 2021 | 184 | 294 | 109 |
| YLDs | Togo | Both | 2021 | 529 | 748 | 349 |
| YLDs | Tokelau | Both | 2021 | 0 | 0 | 0 |
| YLDs | Tonga | Both | 2021 | 10 | 14 | 7 |
| YLDs | Trinidad and Tobago | Both | 2021 | 203 | 281 | 137 |
| YLDs | Tunisia | Both | 2021 | 1432 | 1989 | 965 |
| YLDs | Turkey | Both | 2021 | 14009 | 19667 | 9197 |
| YLDs | Turkmenistan | Both | 2021 | 276 | 388 | 179 |
| YLDs | Tuvalu | Both | 2021 | 1 | 2 | 1 |
| YLDs | Uganda | Both | 2021 | 2214 | 3212 | 1433 |
| YLDs | Ukraine | Both | 2021 | 11908 | 16652 | 8058 |
| YLDs | United Arab Emirates | Both | 2021 | 392 | 539 | 265 |
| YLDs | United Kingdom | Both | 2021 | 60778 | 87198 | 39527 |
| YLDs | United Republic of Tanzania | Both | 2021 | 3678 | 5198 | 2440 |
| YLDs | United States Virgin Islands | Both | 2021 | 30 | 42 | 20 |
| YLDs | United States of America | Both | 2021 | 307109 | 442914 | 197693 |
| YLDs | Uruguay | Both | 2021 | 2404 | 3294 | 1663 |
| YLDs | Uzbekistan | Both | 2021 | 2095 | 2920 | 1389 |
| YLDs | Vanuatu | Both | 2021 | 18 | 26 | 12 |
| YLDs | Venezuela (Bolivarian Republic of) | Both | 2021 | 5358 | 7387 | 3635 |
| YLDs | Viet Nam | Both | 2021 | 27793 | 39107 | 18423 |
| YLDs | Yemen | Both | 2021 | 2199 | 3054 | 1481 |
| YLDs | Zambia | Both | 2021 | 948 | 1343 | 623 |
| YLDs | Zimbabwe | Both | 2021 | 720 | 1015 | 466 |

Abbreviations: YLDs, years lived with disability; HFs, Hip fractures; UI, uncertainty interval.

**Table S5. Joinpoint regression analysis results of ASIR, ASPR, and ASYR for global HFs (≥55 years)**

| **measure** | **location** | **sex** | **joinpoint** | **Start.Obs** | **End.Obs** | **AAPC (%)** | **95% CI_lower** | **95% CI_upper** | **P value** |
| --- | --- | --- | --- | --- | --- | --- | --- | --- | --- |
| ASIR | Global | Both | 5 | 1990 | 2021 | 0.20 | 0.12 | 0.28 | <0.001 |
| ASIR | Global | Female | 5 | 1990 | 2021 | 0.14 | 0.05 | 0.23 | 0.002 |
| ASIR | Global | Male | 2 | 1990 | 2021 | 0.61 | 0.54 | 0.68 | <0.001 |
| ASPR | Global | Both | 4 | 1990 | 2021 | 0.31 | 0.27 | 0.36 | <0.001 |
| ASPR | Global | Female | 4 | 1990 | 2021 | 0.26 | 0.21 | 0.30 | <0.001 |
| ASPR | Global | Male | 5 | 1990 | 2021 | 0.78 | 0.71 | 0.85 | <0.001 |
| ASYR | Global | Both | 5 | 1990 | 2021 | -0.43 | -0.50 | -0.36 | <0.001 |
| ASYR | Global | Female | 5 | 1990 | 2021 | -0.43 | -0.47 | -0.39 | <0.001 |
| ASYR | Global | Male | 5 | 1990 | 2021 | -0.19 | -0.25 | -0.12 | <0.001 |

Abbreviations: ASIR, age-standardized incidence rate; ASPR, age-standardized prevalence rate; ASYR, age-standardized years lived with disability rate; HFs, Hip fractures; CI, confidence interval.

**Table S6. Decomposition analysis results for HFs (≥55 years) across global, 5 SDI regions, and 21 GBD regions**

| **location** | **sex** | **measure** | **percent of Aging (%)** | **percent of Population (%)** | **percent of Epidemiological change (%)** |
| --- | --- | --- | --- | --- | --- |
| Global | Male | Incidence | 12.96 | 72.99 | 14.06 |
| Global | Female | Incidence | 14.84 | 81.60 | 3.57 |
| Global | Both | Incidence | 15.58 | 79.35 | 5.07 |
| East Asia | Male | Incidence | 13.45 | 56.61 | 29.94 |
| East Asia | Female | Incidence | 15.94 | 56.54 | 27.52 |
| East Asia | Both | Incidence | 15.81 | 56.37 | 27.82 |
| Southeast Asia | Male | Incidence | 0.64 | 85.09 | 14.27 |
| Southeast Asia | Female | Incidence | 5.20 | 85.09 | 9.72 |
| Southeast Asia | Both | Incidence | 3.27 | 84.94 | 11.79 |
| Oceania | Male | Incidence | 1.91 | 90.33 | 7.76 |
| Oceania | Female | Incidence | 6.89 | 65.24 | 27.87 |
| Oceania | Both | Incidence | 6.29 | 69.95 | 23.76 |
| Central Asia | Male | Incidence | -3.78 | 105.38 | -1.60 |
| Central Asia | Female | Incidence | -15.62 | 93.84 | 21.78 |
| Central Asia | Both | Incidence | -10.37 | 98.89 | 11.48 |
| Central Europe | Male | Incidence | 28.82 | 71.89 | -0.71 |
| Central Europe | Female | Incidence | 131.51 | 146.90 | -178.42 |
| Central Europe | Both | Incidence | 79.81 | 110.52 | -90.33 |
| Eastern Europe | Male | Incidence | 9.49 | 84.10 | 6.41 |
| Eastern Europe | Female | Incidence | 30.91 | 91.90 | -22.81 |
| Eastern Europe | Both | Incidence | 18.31 | 86.42 | -4.73 |
| High-income Asia Pacific | Male | Incidence | 28.14 | 59.15 | 12.71 |
| High-income Asia Pacific | Female | Incidence | 45.85 | 54.40 | -0.25 |
| High-income Asia Pacific | Both | Incidence | 39.83 | 56.76 | 3.41 |
| Western Europe | Male | Incidence | 30.22 | 55.52 | 14.26 |
| Western Europe | Female | Incidence | 38.29 | 63.90 | -2.19 |
| Western Europe | Both | Incidence | 38.55 | 66.34 | -4.89 |
| Australasia | Male | Incidence | 21.00 | 49.26 | 29.74 |
| Australasia | Female | Incidence | 13.51 | 58.47 | 28.01 |
| Australasia | Both | Incidence | 18.08 | 56.28 | 25.64 |
| Southern Latin America | Male | Incidence | 12.77 | 79.13 | 8.11 |
| Southern Latin America | Female | Incidence | 21.43 | 63.55 | 15.02 |
| Southern Latin America | Both | Incidence | 19.43 | 67.07 | 13.50 |
| High-income North America | Male | Incidence | 5.35 | 59.81 | 34.84 |
| High-income North America | Female | Incidence | -2.73 | 60.18 | 42.55 |
| High-income North America | Both | Incidence | 0.40 | 61.91 | 37.69 |
| Caribbean | Male | Incidence | 7.18 | 63.97 | 28.85 |
| Caribbean | Female | Incidence | 13.21 | 57.00 | 29.78 |
| Caribbean | Both | Incidence | 11.32 | 58.02 | 30.67 |
| Andean Latin America | Male | Incidence | 3.06 | 88.04 | 8.90 |
| Andean Latin America | Female | Incidence | 6.03 | 77.08 | 16.89 |
| Andean Latin America | Both | Incidence | 4.65 | 81.67 | 13.69 |
| Central Latin America | Male | Incidence | 5.30 | 125.65 | -30.95 |
| Central Latin America | Female | Incidence | 12.85 | 118.00 | -30.85 |
| Central Latin America | Both | Incidence | 9.36 | 120.10 | -29.47 |
| Tropical Latin America | Male | Incidence | 8.38 | 95.27 | -3.65 |
| Tropical Latin America | Female | Incidence | 14.77 | 80.95 | 4.27 |
| Tropical Latin America | Both | Incidence | 12.69 | 85.16 | 2.15 |
| North Africa and Middle East | Male | Incidence | 3.32 | 91.24 | 5.44 |
| North Africa and Middle East | Female | Incidence | 3.91 | 72.67 | 23.42 |
| North Africa and Middle East | Both | Incidence | 4.34 | 79.65 | 16.01 |
| South Asia | Male | Incidence | 9.40 | 83.54 | 7.06 |
| South Asia | Female | Incidence | 12.80 | 78.80 | 8.40 |
| South Asia | Both | Incidence | 11.55 | 77.48 | 10.97 |
| Central Sub-Saharan Africa | Male | Incidence | -5.87 | 106.78 | -0.91 |
| Central Sub-Saharan Africa | Female | Incidence | 10.27 | 80.54 | 9.19 |
| Central Sub-Saharan Africa | Both | Incidence | 3.04 | 88.96 | 8.00 |
| Eastern Sub-Saharan Africa | Male | Incidence | -0.15 | 111.23 | -11.08 |
| Eastern Sub-Saharan Africa | Female | Incidence | 6.31 | 90.70 | 2.99 |
| Eastern Sub-Saharan Africa | Both | Incidence | 3.74 | 97.16 | -0.89 |
| Southern Sub-Saharan Africa | Male | Incidence | -5.51 | 122.15 | -16.64 |
| Southern Sub-Saharan Africa | Female | Incidence | -6.86 | 123.59 | -16.73 |
| Southern Sub-Saharan Africa | Both | Incidence | -6.45 | 122.97 | -16.52 |
| High-middle SDI | Male | Incidence | 15.30 | 77.79 | 6.91 |
| High-middle SDI | Female | Incidence | 21.79 | 79.07 | -0.86 |
| High-middle SDI | Both | Incidence | 20.32 | 80.12 | -0.44 |
| Western Sub-Saharan Africa | Male | Incidence | 3.35 | 83.96 | 12.69 |
| Western Sub-Saharan Africa | Female | Incidence | -15.20 | 99.00 | 16.20 |
| Western Sub-Saharan Africa | Both | Incidence | -4.60 | 90.76 | 13.84 |
| Middle SDI | Male | Incidence | 8.85 | 70.15 | 21.00 |
| Middle SDI | Female | Incidence | 11.07 | 67.83 | 21.10 |
| Middle SDI | Both | Incidence | 10.97 | 68.17 | 20.86 |
| High SDI | Male | Incidence | 18.49 | 60.47 | 21.04 |
| High SDI | Female | Incidence | 21.18 | 68.22 | 10.59 |
| High SDI | Both | Incidence | 21.53 | 68.47 | 9.99 |
| Low-middle SDI | Male | Incidence | 6.18 | 89.13 | 4.69 |
| Low-middle SDI | Female | Incidence | 9.67 | 81.97 | 8.36 |
| Low-middle SDI | Both | Incidence | 8.43 | 81.97 | 9.60 |
| Low SDI | Male | Incidence | 5.12 | 87.53 | 7.36 |
| Low SDI | Female | Incidence | 5.70 | 77.25 | 17.05 |
| Low SDI | Both | Incidence | 5.87 | 79.27 | 14.87 |
| Global | Male | Prevalence | 10.86 | 71.65 | 17.48 |
| Global | Female | Prevalence | 12.40 | 79.84 | 7.76 |
| Global | Both | Prevalence | 13.53 | 77.95 | 8.52 |
| East Asia | Male | Prevalence | 9.45 | 60.31 | 30.25 |
| East Asia | Female | Prevalence | 13.27 | 57.39 | 29.33 |
| East Asia | Both | Prevalence | 13.19 | 58.01 | 28.79 |
| Southeast Asia | Male | Prevalence | 0.36 | 79.45 | 20.19 |
| Southeast Asia | Female | Prevalence | 4.88 | 80.87 | 14.25 |
| Southeast Asia | Both | Prevalence | 3.00 | 80.29 | 16.71 |
| Oceania | Male | Prevalence | 1.23 | 81.89 | 16.88 |
| Oceania | Female | Prevalence | 5.90 | 63.59 | 30.51 |
| Oceania | Both | Prevalence | 5.36 | 67.14 | 27.50 |
| Central Asia | Male | Prevalence | -3.00 | 119.38 | -16.38 |
| Central Asia | Female | Prevalence | -16.96 | 118.38 | -1.42 |
| Central Asia | Both | Prevalence | -10.47 | 120.19 | -9.72 |
| Central Europe | Male | Prevalence | 21.41 | 58.91 | 19.68 |
| Central Europe | Female | Prevalence | 84.17 | 99.04 | -83.21 |
| Central Europe | Both | Prevalence | 58.49 | 83.61 | -42.11 |
| Eastern Europe | Male | Prevalence | 7.67 | 82.14 | 10.19 |
| Eastern Europe | Female | Prevalence | 21.40 | 79.61 | -1.01 |
| Eastern Europe | Both | Prevalence | 16.10 | 82.73 | 1.17 |
| High-income Asia Pacific | Male | Prevalence | 25.17 | 61.77 | 13.05 |
| High-income Asia Pacific | Female | Prevalence | 38.46 | 55.26 | 6.29 |
| High-income Asia Pacific | Both | Prevalence | 34.19 | 58.20 | 7.61 |
| Western Europe | Male | Prevalence | 21.57 | 55.43 | 23.00 |
| Western Europe | Female | Prevalence | 26.81 | 58.73 | 14.46 |
| Western Europe | Both | Prevalence | 28.31 | 62.40 | 9.30 |
| Australasia | Male | Prevalence | 17.04 | 50.64 | 32.32 |
| Australasia | Female | Prevalence | 10.88 | 60.58 | 28.54 |
| Australasia | Both | Prevalence | 15.30 | 58.26 | 26.44 |
| Southern Latin America | Male | Prevalence | 7.81 | 74.02 | 18.17 |
| Southern Latin America | Female | Prevalence | 17.15 | 64.32 | 18.54 |
| Southern Latin America | Both | Prevalence | 14.77 | 66.79 | 18.44 |
| High-income North America | Male | Prevalence | 3.55 | 58.50 | 37.95 |
| High-income North America | Female | Prevalence | -4.23 | 58.16 | 46.07 |
| High-income North America | Both | Prevalence | -0.96 | 60.30 | 40.66 |
| Caribbean | Male | Prevalence | 5.82 | 63.29 | 30.88 |
| Caribbean | Female | Prevalence | 12.21 | 56.52 | 31.27 |
| Caribbean | Both | Prevalence | 10.28 | 57.27 | 32.45 |
| Andean Latin America | Male | Prevalence | 2.70 | 81.36 | 15.94 |
| Andean Latin America | Female | Prevalence | 5.22 | 75.31 | 19.47 |
| Andean Latin America | Both | Prevalence | 4.11 | 77.76 | 18.13 |
| Central Latin America | Male | Prevalence | 4.13 | 121.12 | -25.25 |
| Central Latin America | Female | Prevalence | 11.20 | 114.53 | -25.74 |
| Central Latin America | Both | Prevalence | 8.04 | 115.90 | -23.95 |
| Tropical Latin America | Male | Prevalence | 6.00 | 88.76 | 5.25 |
| Tropical Latin America | Female | Prevalence | 12.96 | 75.26 | 11.78 |
| Tropical Latin America | Both | Prevalence | 10.80 | 78.86 | 10.34 |
| North Africa and Middle East | Male | Prevalence | 1.96 | 79.15 | 18.89 |
| North Africa and Middle East | Female | Prevalence | 3.39 | 71.07 | 25.54 |
| North Africa and Middle East | Both | Prevalence | 3.37 | 74.29 | 22.34 |
| South Asia | Male | Prevalence | 7.50 | 71.15 | 21.36 |
| South Asia | Female | Prevalence | 11.32 | 69.29 | 19.39 |
| South Asia | Both | Prevalence | 10.08 | 67.33 | 22.58 |
| Central Sub-Saharan Africa | Male | Prevalence | -2.50 | 80.58 | 21.91 |
| Central Sub-Saharan Africa | Female | Prevalence | 9.52 | 74.13 | 16.35 |
| Central Sub-Saharan Africa | Both | Prevalence | 3.15 | 76.07 | 20.79 |
| Eastern Sub-Saharan Africa | Male | Prevalence | 0.62 | 76.57 | 22.81 |
| Eastern Sub-Saharan Africa | Female | Prevalence | 5.79 | 74.87 | 19.34 |
| Eastern Sub-Saharan Africa | Both | Prevalence | 3.61 | 74.42 | 21.97 |
| Southern Sub-Saharan Africa | Male | Prevalence | -5.58 | 216.00 | -110.42 |
| Southern Sub-Saharan Africa | Female | Prevalence | -8.58 | 221.26 | -112.67 |
| Southern Sub-Saharan Africa | Both | Prevalence | -8.05 | 218.53 | -110.48 |
| High-middle SDI | Male | Prevalence | 12.81 | 81.37 | 5.83 |
| High-middle SDI | Female | Prevalence | 18.05 | 78.20 | 3.74 |
| High-middle SDI | Both | Prevalence | 17.67 | 80.94 | 1.39 |
| Western Sub-Saharan Africa | Male | Prevalence | 2.99 | 75.42 | 21.59 |
| Western Sub-Saharan Africa | Female | Prevalence | -13.80 | 90.50 | 23.29 |
| Western Sub-Saharan Africa | Both | Prevalence | -3.83 | 82.20 | 21.63 |
| Middle SDI | Male | Prevalence | 6.86 | 70.72 | 22.43 |
| Middle SDI | Female | Prevalence | 9.74 | 66.30 | 23.96 |
| Middle SDI | Both | Prevalence | 9.64 | 67.14 | 23.23 |
| High SDI | Male | Prevalence | 14.16 | 59.29 | 26.55 |
| High SDI | Female | Prevalence | 15.06 | 63.98 | 20.96 |
| High SDI | Both | Prevalence | 16.30 | 65.24 | 18.46 |
| Low-middle SDI | Male | Prevalence | 4.62 | 76.41 | 18.96 |
| Low-middle SDI | Female | Prevalence | 8.53 | 72.73 | 18.74 |
| Low-middle SDI | Both | Prevalence | 7.22 | 71.77 | 21.00 |
| Low SDI | Male | Prevalence | 3.91 | 70.68 | 25.41 |
| Low SDI | Female | Prevalence | 5.15 | 67.11 | 27.74 |
| Low SDI | Both | Prevalence | 5.13 | 66.84 | 28.03 |
| Global | Male | YLDs | 12.86 | 103.10 | -15.96 |
| Global | Female | YLDs | 13.84 | 106.43 | -20.27 |
| Global | Both | YLDs | 15.54 | 106.22 | -21.76 |
| East Asia | Male | YLDs | 18.10 | 132.51 | -50.62 |
| East Asia | Female | YLDs | 23.82 | 124.13 | -47.95 |
| East Asia | Both | YLDs | 24.18 | 126.43 | -50.61 |
| Southeast Asia | Male | YLDs | 0.41 | 113.44 | -13.85 |
| Southeast Asia | Female | YLDs | 6.32 | 119.09 | -25.40 |
| Southeast Asia | Both | YLDs | 3.75 | 117.04 | -20.80 |
| Oceania | Male | YLDs | 1.03 | 88.94 | 10.03 |
| Oceania | Female | YLDs | 5.74 | 67.76 | 26.50 |
| Oceania | Both | YLDs | 5.19 | 71.81 | 22.99 |
| Central Asia | Male | YLDs | -4.63 | 199.21 | -94.58 |
| Central Asia | Female | YLDs | -34.91 | 265.54 | -130.63 |
| Central Asia | Both | YLDs | -18.25 | 233.30 | -115.05 |
| Central Europe | Male | YLDs | 671.49 | 2041.29 | -2612.78 |
| Central Europe | Female | YLDs | -118.88 | -146.45 | 365.33 |
| Central Europe | Both | YLDs | -143.13 | -216.81 | 459.93 |
| Eastern Europe | Male | YLDs | -250.05 | -3402.88 | 3752.93 |
| Eastern Europe | Female | YLDs | -21.93 | -90.52 | 212.45 |
| Eastern Europe | Both | YLDs | -28.93 | -169.52 | 298.45 |
| High-income Asia Pacific | Male | YLDs | 27.96 | 73.44 | -1.40 |
| High-income Asia Pacific | Female | YLDs | 44.84 | 66.31 | -11.15 |
| High-income Asia Pacific | Both | YLDs | 39.39 | 69.67 | -9.06 |
| Western Europe | Male | YLDs | 27.22 | 66.75 | 6.03 |
| Western Europe | Female | YLDs | 37.57 | 79.87 | -17.44 |
| Western Europe | Both | YLDs | 38.14 | 81.80 | -19.95 |
| Australasia | Male | YLDs | 17.96 | 50.74 | 31.30 |
| Australasia | Female | YLDs | 11.37 | 60.66 | 27.97 |
| Australasia | Both | YLDs | 15.85 | 58.29 | 25.86 |
| Southern Latin America | Male | YLDs | 21.15 | 219.29 | -140.44 |
| Southern Latin America | Female | YLDs | 36.85 | 146.54 | -83.38 |
| Southern Latin America | Both | YLDs | 33.56 | 161.59 | -95.16 |
| High-income North America | Male | YLDs | 4.02 | 62.21 | 33.76 |
| High-income North America | Female | YLDs | -4.19 | 62.74 | 41.45 |
| High-income North America | Both | YLDs | -0.76 | 64.71 | 36.06 |
| Caribbean | Male | YLDs | 6.86 | 91.24 | 1.90 |
| Caribbean | Female | YLDs | 14.77 | 79.08 | 6.15 |
| Caribbean | Both | YLDs | 12.25 | 80.92 | 6.83 |
| Andean Latin America | Male | YLDs | 3.86 | 136.17 | -40.03 |
| Andean Latin America | Female | YLDs | 7.27 | 118.81 | -26.08 |
| Andean Latin America | Both | YLDs | 5.79 | 125.83 | -31.62 |
| Central Latin America | Male | YLDs | 6.23 | 209.04 | -115.27 |
| Central Latin America | Female | YLDs | 16.12 | 187.43 | -103.56 |
| Central Latin America | Both | YLDs | 11.68 | 193.30 | -104.99 |
| Tropical Latin America | Male | YLDs | 7.99 | 132.48 | -40.47 |
| Tropical Latin America | Female | YLDs | 16.50 | 104.10 | -20.59 |
| Tropical Latin America | Both | YLDs | 13.89 | 111.81 | -25.70 |
| North Africa and Middle East | Male | YLDs | 2.79 | 147.53 | -50.32 |
| North Africa and Middle East | Female | YLDs | 5.04 | 123.39 | -28.43 |
| North Africa and Middle East | Both | YLDs | 4.94 | 133.12 | -38.06 |
| South Asia | Male | YLDs | 9.32 | 96.36 | -5.68 |
| South Asia | Female | YLDs | 13.67 | 90.56 | -4.23 |
| South Asia | Both | YLDs | 12.23 | 88.61 | -0.84 |
| Central Sub-Saharan Africa | Male | YLDs | -2.71 | 94.99 | 7.72 |
| Central Sub-Saharan Africa | Female | YLDs | 10.20 | 86.31 | 3.49 |
| Central Sub-Saharan Africa | Both | YLDs | 3.25 | 88.91 | 7.84 |
| Eastern Sub-Saharan Africa | Male | YLDs | 0.55 | 93.16 | 6.29 |
| Eastern Sub-Saharan Africa | Female | YLDs | 6.30 | 89.88 | 3.82 |
| Eastern Sub-Saharan Africa | Both | YLDs | 3.83 | 89.80 | 6.37 |
| Southern Sub-Saharan Africa | Male | YLDs | -8.40 | 367.51 | -259.10 |
| Southern Sub-Saharan Africa | Female | YLDs | -12.71 | 349.43 | -236.72 |
| Southern Sub-Saharan Africa | Both | YLDs | -12.05 | 354.88 | -242.82 |
| High-middle SDI | Male | YLDs | 22.03 | 165.13 | -87.16 |
| High-middle SDI | Female | YLDs | 31.70 | 156.31 | -88.01 |
| High-middle SDI | Both | YLDs | 30.83 | 162.55 | -93.38 |
| Western Sub-Saharan Africa | Male | YLDs | 3.16 | 91.56 | 5.29 |
| Western Sub-Saharan Africa | Female | YLDs | -16.09 | 110.33 | 5.76 |
| Western Sub-Saharan Africa | Both | YLDs | -4.53 | 100.13 | 4.41 |
| Middle SDI | Male | YLDs | 10.60 | 122.85 | -33.45 |
| Middle SDI | Female | YLDs | 13.80 | 106.90 | -20.71 |
| Middle SDI | Both | YLDs | 14.07 | 110.75 | -24.82 |
| High SDI | Male | YLDs | 16.85 | 70.79 | 12.37 |
| High SDI | Female | YLDs | 19.62 | 82.13 | -1.75 |
| High SDI | Both | YLDs | 20.57 | 81.85 | -2.41 |
| Low-middle SDI | Male | YLDs | 5.76 | 103.82 | -9.58 |
| Low-middle SDI | Female | YLDs | 10.21 | 94.29 | -4.50 |
| Low-middle SDI | Both | YLDs | 8.72 | 94.10 | -2.82 |
| Low SDI | Male | YLDs | 4.38 | 88.49 | 7.13 |
| Low SDI | Female | YLDs | 5.89 | 82.44 | 11.67 |
| Low SDI | Both | YLDs | 5.83 | 82.63 | 11.54 |

Abbreviations: YLDs, years lived with disability; HFs, Hip fractures; SDI, sociodemographic index; GBD, Global Burden of Disease Study.

**Table S7. Predictive analysis results for global HFs (≥55 years)**

| **measure** | **location** | **sex** | **year** | **predictive value** | **95% CI_lower** | **95% CI_upper** |
| --- | --- | --- | --- | --- | --- | --- |
| ASIR | Global | Male | 2050 | 728.44 | 79.50 | 1377.38 |
| ASIR | Global | Female | 2050 | 1192.78 | 220.04 | 2165.51 |
| ASIR | Global | Both | 2050 | 1102.66 | 101.41 | 2142.83 |
| ASPR | Global | Male | 2050 | 1270.02 | 29.04 | 2511.01 |
| ASPR | Global | Female | 2050 | 2248.46 | 382.07 | 4114.86 |
| ASPR | Global | Both | 2050 | 2052.14 | 141.30 | 4112.55 |
| ASYR | Global | Male | 2050 | 109.01 | -2.24 | 220.26 |
| ASYR | Global | Female | 2050 | 185.89 | 11.72 | 360.07 |
| ASYR | Global | Both | 2050 | 174.43 | 0.00 | 365.91 |
| new case | Global | Male | 2050 | 9213751 | 1005584 | 17421918 |
| new case | Global | Female | 2050 | 17179686 | 3169282 | 31190090 |
| new case | Global | Both | 2050 | 26393437 | 4174866 | 48612008 |
| prevalent count | Global | Male | 2050 | 16063975 | 367272 | 31760679 |
| prevalent count | Global | Female | 2050 | 32384820 | 5502959 | 59266681 |
| prevalent count | Global | Both | 2050 | 48448795 | 5870231 | 91027360 |
| YLDs | Global | Male | 2050 | 1378861 | -28288 | 2786010 |
| YLDs | Global | Female | 2050 | 2677424 | 168739 | 5186109 |
| YLDs | Global | Both | 2050 | 4056285 | 140451 | 7972119 |

Abbreviations: ASIR, age-standardized incidence rate; ASPR, age-standardized prevalence rate; ASYR, age-standardized years lived with disability rate; HFs, Hip fractures; YLDs, years lived with disability; CI, confidence interval.

**Table S8a. Frontier analysis results of ASIR for HFs (≥55 years) across 204 countries and territories**

| **Location** | **SDI** | **Effective difference** |
| --- | --- | --- |
| Andorra | 0.87 | 3302.86 |
| Norway | 0.92 | 2746.52 |
| Netherlands | 0.89 | 2679.97 |
| Switzerland | 0.93 | 2598.73 |
| Belgium | 0.85 | 2454.32 |
| Croatia | 0.80 | 2380.57 |
| Australia | 0.84 | 2376.66 |
| France | 0.84 | 2278.38 |
| Greenland | 0.83 | 2256.99 |
| Finland | 0.86 | 2238.68 |
| Slovenia | 0.84 | 2200.09 |
| Canada | 0.87 | 2151.01 |
| Cyprus | 0.84 | 2125.56 |
| New Zealand | 0.85 | 2038.45 |
| Luxembourg | 0.88 | 1964.25 |
| Sweden | 0.89 | 1962.38 |
| Austria | 0.85 | 1797.62 |
| Germany | 0.90 | 1735.52 |
| Denmark | 0.90 | 1658.76 |
| Republic of Korea | 0.89 | 1596.97 |
| Cuba | 0.67 | 1542.90 |
| United States of America | 0.86 | 1499.17 |
| Italy | 0.81 | 1457.65 |
| Hungary | 0.79 | 1427.48 |
| Czechia | 0.83 | 1406.47 |
| United Kingdom | 0.86 | 1349.28 |
| Iceland | 0.88 | 1324.76 |
| Malta | 0.80 | 1237.00 |
| Slovakia | 0.81 | 1209.12 |
| Ireland | 0.87 | 1183.97 |
| Chile | 0.77 | 1107.44 |
| India | 0.58 | 1091.52 |
| San Marino | 0.89 | 1052.28 |
| Spain | 0.77 | 927.07 |
| Monaco | 0.91 | 925.44 |
| Saudi Arabia | 0.82 | 921.83 |
| Poland | 0.81 | 919.09 |
| Viet Nam | 0.63 | 910.77 |
| Portugal | 0.74 | 826.88 |
| Libya | 0.73 | 823.35 |
| Oman | 0.77 | 804.39 |
| Brunei Darussalam | 0.81 | 800.45 |
| Uruguay | 0.72 | 799.30 |
| Israel | 0.81 | 790.39 |
| Bhutan | 0.47 | 768.55 |
| North Macedonia | 0.75 | 733.90 |
| Solomon Islands | 0.43 | 729.07 |
| Montenegro | 0.80 | 722.78 |
| China | 0.72 | 714.62 |
| Lithuania | 0.86 | 671.71 |
| Sri Lanka | 0.70 | 669.90 |
| Palau | 0.75 | 668.98 |
| Serbia | 0.79 | 646.54 |
| Costa Rica | 0.70 | 641.15 |
| Cambodia | 0.47 | 638.57 |
| Japan | 0.87 | 606.78 |
| Papua New Guinea | 0.42 | 603.56 |
| Northern Mariana Islands | 0.77 | 602.26 |
| Nicaragua | 0.52 | 551.68 |
| Nepal | 0.43 | 546.83 |
| Greece | 0.79 | 520.51 |
| Brazil | 0.65 | 510.63 |
| Latvia | 0.83 | 509.46 |
| Mexico | 0.66 | 507.00 |
| Bosnia and Herzegovina | 0.72 | 484.24 |
| Turkey | 0.71 | 481.90 |
| Singapore | 0.86 | 479.43 |
| Sao Tome and Principe | 0.51 | 465.87 |
| Argentina | 0.72 | 460.60 |
| Belarus | 0.78 | 458.97 |
| Guatemala | 0.54 | 454.39 |
| Qatar | 0.85 | 449.42 |
| El Salvador | 0.56 | 438.71 |
| Russian Federation | 0.81 | 437.69 |
| Romania | 0.77 | 431.06 |
| Myanmar | 0.53 | 428.67 |
| Indonesia | 0.66 | 418.24 |
| Estonia | 0.84 | 416.06 |
| Puerto Rico | 0.83 | 414.20 |
| Morocco | 0.56 | 413.46 |
| Guyana | 0.65 | 400.15 |
| Bulgaria | 0.77 | 390.39 |
| Saint Kitts and Nevis | 0.75 | 383.56 |
| United States Virgin Islands | 0.82 | 377.51 |
| Kenya | 0.52 | 375.81 |
| Maldives | 0.65 | 369.65 |
| Albania | 0.71 | 359.64 |
| Nauru | 0.63 | 359.09 |
| Afghanistan | 0.34 | 352.12 |
| Malaysia | 0.74 | 350.16 |
| Grenada | 0.67 | 337.82 |
| Burkina Faso | 0.29 | 336.88 |
| Bermuda | 0.82 | 328.55 |
| Paraguay | 0.64 | 325.76 |
| Antigua and Barbuda | 0.75 | 324.23 |
| Ecuador | 0.66 | 323.12 |
| Venezuela (Bolivarian Republic of) | 0.60 | 322.53 |
| Micronesia (Federated States of) | 0.59 | 321.52 |
| Georgia | 0.73 | 320.14 |
| Cabo Verde | 0.53 | 319.79 |
| Ethiopia | 0.36 | 319.19 |
| Zambia | 0.51 | 318.96 |
| Gambia | 0.41 | 318.07 |
| Nigeria | 0.50 | 315.92 |
| Gabon | 0.63 | 312.43 |
| Bolivia (Plurinational State of) | 0.60 | 311.86 |
| Mali | 0.27 | 309.90 |
| Coted'Ivoire | 0.43 | 300.95 |
| American Samoa | 0.72 | 296.38 |
| Ghana | 0.56 | 294.84 |
| Cameroon | 0.48 | 294.10 |
| Iran (Islamic Republic of) | 0.70 | 292.82 |
| Samoa | 0.59 | 291.96 |
| Niue | 0.73 | 286.35 |
| Senegal | 0.41 | 281.93 |
| Palestine | 0.63 | 280.19 |
| Mauritania | 0.50 | 277.70 |
| Mozambique | 0.33 | 276.73 |
| Eritrea | 0.40 | 276.22 |
| Equatorial Guinea | 0.66 | 275.98 |
| Togo | 0.41 | 274.03 |
| Ukraine | 0.76 | 272.68 |
| Yemen | 0.45 | 272.20 |
| Honduras | 0.51 | 270.18 |
| Guinea-Bissau | 0.35 | 269.16 |
| Tokelau | 0.69 | 267.76 |
| Benin | 0.37 | 265.80 |
| Bahamas | 0.81 | 264.77 |
| Barbados | 0.75 | 263.19 |
| Tuvalu | 0.58 | 261.66 |
| Thailand | 0.68 | 260.50 |
| Uganda | 0.42 | 258.19 |
| Saint Vincent and the Grenadines | 0.64 | 256.56 |
| Tunisia | 0.68 | 254.21 |
| Rwanda | 0.44 | 254.04 |
| United Republic of Tanzania | 0.45 | 251.64 |
| Djibouti | 0.49 | 250.68 |
| Republic of Moldova | 0.73 | 249.04 |
| Malawi | 0.38 | 247.19 |
| Belize | 0.61 | 245.90 |
| Taiwan (Province of China) | 0.87 | 243.73 |
| Algeria | 0.66 | 243.49 |
| Dominican Republic | 0.62 | 240.65 |
| Kazakhstan | 0.73 | 240.30 |
| Liberia | 0.35 | 238.92 |
| Suriname | 0.63 | 236.74 |
| Angola | 0.45 | 232.96 |
| Haiti | 0.45 | 231.20 |
| Comoros | 0.48 | 231.00 |
| Timor-Leste | 0.44 | 230.23 |
| Congo | 0.58 | 229.49 |
| Guinea | 0.34 | 227.94 |
| Marshall Islands | 0.57 | 225.33 |
| Lebanon | 0.74 | 223.07 |
| Cook Islands | 0.78 | 221.78 |
| Democratic Republic of the Congo | 0.38 | 221.17 |
| Sierra Leone | 0.36 | 218.71 |
| Zimbabwe | 0.47 | 210.96 |
| Lao People's Democratic Republic | 0.49 | 210.27 |
| Philippines | 0.65 | 209.50 |
| Saint Lucia | 0.67 | 208.41 |
| Colombia | 0.66 | 206.27 |
| Syrian Arab Republic | 0.62 | 199.85 |
| Burundi | 0.29 | 199.78 |
| Iraq | 0.66 | 196.14 |
| Tonga | 0.63 | 190.06 |
| United Arab Emirates | 0.85 | 186.66 |
| South Sudan | 0.28 | 186.40 |
| Bahrain | 0.75 | 185.19 |
| Peru | 0.66 | 183.97 |
| Panama | 0.71 | 182.15 |
| Armenia | 0.70 | 179.92 |
| Sudan | 0.54 | 176.10 |
| Central African Republic | 0.31 | 172.79 |
| Guam | 0.80 | 167.78 |
| Kuwait | 0.85 | 166.74 |
| Vanuatu | 0.47 | 162.92 |
| Chad | 0.24 | 160.92 |
| Egypt | 0.61 | 160.15 |
| Jordan | 0.73 | 155.91 |
| Botswana | 0.64 | 154.80 |
| Jamaica | 0.68 | 154.73 |
| Dominica | 0.75 | 152.65 |
| Mongolia | 0.62 | 151.80 |
| Lesotho | 0.51 | 145.94 |
| Seychelles | 0.73 | 145.08 |
| Fiji | 0.68 | 139.11 |
| Madagascar | 0.40 | 137.15 |
| Namibia | 0.62 | 136.28 |
| Niger | 0.17 | 125.81 |
| Trinidad and Tobago | 0.77 | 124.93 |
| Pakistan | 0.50 | 122.96 |
| Uzbekistan | 0.66 | 112.85 |
| Eswatini | 0.59 | 110.55 |
| Democratic People's Republic of Korea | 0.57 | 96.53 |
| Kyrgyzstan | 0.60 | 95.82 |
| Mauritius | 0.72 | 92.91 |
| South Africa | 0.68 | 90.32 |
| Azerbaijan | 0.69 | 77.27 |
| Tajikistan | 0.54 | 70.70 |
| Kiribati | 0.53 | 53.59 |
| Turkmenistan | 0.68 | 45.62 |
| Somalia | 0.08 | 39.80 |
| Bangladesh | 0.49 | 36.29 |

Abbreviations: ASIR, age-standardized incidence rate; HFs, Hip fractures; SDI, sociodemographic index.

**Table S8b. Frontier analysis results of ASPR for HFs (≥55 years) across 204 countries and territories**

| **Location** | **SDI** | **Effective difference** |
| --- | --- | --- |
| Andorra | 0.87 | 7177.43 |
| Belgium | 0.85 | 6065.91 |
| Switzerland | 0.93 | 5739.54 |
| Finland | 0.86 | 5561.50 |
| Norway | 0.92 | 5434.99 |
| France | 0.84 | 5421.99 |
| Netherlands | 0.89 | 5234.21 |
| Australia | 0.84 | 4965.98 |
| Greenland | 0.83 | 4900.40 |
| Canada | 0.87 | 4837.42 |
| Luxembourg | 0.88 | 4585.83 |
| Austria | 0.85 | 4403.51 |
| New Zealand | 0.85 | 4314.77 |
| Sweden | 0.89 | 4266.30 |
| United States of America | 0.86 | 4130.94 |
| Republic of Korea | 0.89 | 4101.55 |
| Germany | 0.90 | 4047.25 |
| Cyprus | 0.84 | 3989.28 |
| Italy | 0.81 | 3705.69 |
| Slovenia | 0.84 | 3562.77 |
| Denmark | 0.90 | 3509.29 |
| Croatia | 0.80 | 3351.26 |
| Iceland | 0.88 | 3312.45 |
| United Kingdom | 0.86 | 3224.97 |
| Malta | 0.80 | 3193.07 |
| Ireland | 0.87 | 3027.57 |
| San Marino | 0.89 | 2728.40 |
| Chile | 0.77 | 2701.84 |
| Spain | 0.77 | 2697.33 |
| Portugal | 0.74 | 2396.28 |
| Cuba | 0.67 | 2339.10 |
| Monaco | 0.91 | 2275.47 |
| Hungary | 0.79 | 2208.94 |
| Czechia | 0.83 | 2190.64 |
| Israel | 0.81 | 2056.40 |
| Uruguay | 0.72 | 2005.32 |
| Slovakia | 0.81 | 1948.99 |
| Japan | 0.87 | 1895.28 |
| Brunei Darussalam | 0.81 | 1846.77 |
| Greece | 0.79 | 1704.93 |
| Singapore | 0.86 | 1689.73 |
| India | 0.58 | 1613.62 |
| Saudi Arabia | 0.82 | 1586.36 |
| Poland | 0.81 | 1543.44 |
| Viet Nam | 0.63 | 1364.92 |
| Argentina | 0.72 | 1295.65 |
| Lithuania | 0.86 | 1284.27 |
| China | 0.72 | 1135.45 |
| Sri Lanka | 0.70 | 1130.54 |
| Bhutan | 0.47 | 1122.73 |
| Costa Rica | 0.70 | 1112.80 |
| Montenegro | 0.80 | 1065.67 |
| Nicaragua | 0.52 | 1057.64 |
| Cambodia | 0.47 | 1019.77 |
| Solomon Islands | 0.43 | 983.46 |
| Brazil | 0.65 | 982.98 |
| El Salvador | 0.56 | 932.37 |
| Oman | 0.77 | 931.76 |
| Latvia | 0.83 | 931.04 |
| Papua New Guinea | 0.42 | 893.75 |
| Serbia | 0.79 | 888.19 |
| Nepal | 0.43 | 885.43 |
| Palau | 0.75 | 878.21 |
| North Macedonia | 0.75 | 876.39 |
| Mexico | 0.66 | 863.39 |
| Northern Mariana Islands | 0.77 | 862.61 |
| Puerto Rico | 0.83 | 852.40 |
| Belarus | 0.78 | 851.83 |
| Estonia | 0.84 | 827.68 |
| Turkey | 0.71 | 797.22 |
| Russian Federation | 0.81 | 793.75 |
| Romania | 0.77 | 784.46 |
| Libya | 0.73 | 772.11 |
| Guatemala | 0.54 | 767.87 |
| Bosnia and Herzegovina | 0.72 | 761.31 |
| Myanmar | 0.53 | 718.08 |
| Sao Tome and Principe | 0.51 | 621.98 |
| Georgia | 0.73 | 612.81 |
| Indonesia | 0.66 | 610.95 |
| Morocco | 0.56 | 609.88 |
| Paraguay | 0.64 | 602.98 |
| Thailand | 0.68 | 598.95 |
| Bulgaria | 0.77 | 593.32 |
| Ukraine | 0.76 | 581.91 |
| Venezuela (Bolivarian Republic of) | 0.60 | 578.73 |
| Iran (Islamic Republic of) | 0.70 | 578.69 |
| Qatar | 0.85 | 572.55 |
| Maldives | 0.65 | 566.88 |
| Palestine | 0.63 | 564.22 |
| Albania | 0.71 | 553.26 |
| Lebanon | 0.74 | 548.63 |
| Ecuador | 0.66 | 544.37 |
| Bermuda | 0.82 | 542.20 |
| Taiwan (Province of China) | 0.87 | 537.58 |
| Malaysia | 0.74 | 535.46 |
| Iraq | 0.66 | 535.03 |
| Afghanistan | 0.34 | 519.30 |
| Guyana | 0.65 | 518.59 |
| United States Virgin Islands | 0.82 | 501.88 |
| Timor-Leste | 0.44 | 498.95 |
| Republic of Moldova | 0.73 | 479.06 |
| Colombia | 0.66 | 472.27 |
| Saint Kitts and Nevis | 0.75 | 469.10 |
| Samoa | 0.59 | 463.25 |
| Nigeria | 0.50 | 457.39 |
| Tunisia | 0.68 | 455.54 |
| Rwanda | 0.44 | 454.22 |
| Bahamas | 0.81 | 451.27 |
| Cabo Verde | 0.53 | 450.32 |
| Algeria | 0.66 | 449.22 |
| Bolivia (Plurinational State of) | 0.60 | 446.55 |
| Kenya | 0.52 | 444.05 |
| Grenada | 0.67 | 428.96 |
| Eritrea | 0.40 | 425.38 |
| Kazakhstan | 0.73 | 398.88 |
| Panama | 0.71 | 397.32 |
| Peru | 0.66 | 396.40 |
| Nauru | 0.63 | 394.99 |
| Suriname | 0.63 | 393.85 |
| Gabon | 0.63 | 393.28 |
| Gambia | 0.41 | 391.22 |
| Ghana | 0.56 | 390.68 |
| Belize | 0.61 | 390.01 |
| United Arab Emirates | 0.85 | 389.68 |
| Syrian Arab Republic | 0.62 | 384.95 |
| Senegal | 0.41 | 383.79 |
| Niue | 0.73 | 383.67 |
| Cameroon | 0.48 | 383.34 |
| Burkina Faso | 0.29 | 381.05 |
| Honduras | 0.51 | 380.75 |
| Antigua and Barbuda | 0.75 | 377.67 |
| Cook Islands | 0.78 | 377.60 |
| American Samoa | 0.72 | 376.54 |
| Mauritania | 0.50 | 376.17 |
| Barbados | 0.75 | 375.62 |
| Coted'Ivoire | 0.43 | 371.61 |
| Ethiopia | 0.36 | 370.25 |
| Mali | 0.27 | 368.31 |
| Togo | 0.41 | 367.73 |
| Yemen | 0.45 | 363.60 |
| Uganda | 0.42 | 360.26 |
| Dominican Republic | 0.62 | 356.90 |
| Philippines | 0.65 | 356.80 |
| Micronesia (Federated States of) | 0.59 | 350.56 |
| Benin | 0.37 | 348.73 |
| Zambia | 0.51 | 348.65 |
| Equatorial Guinea | 0.66 | 346.20 |
| Tokelau | 0.69 | 344.75 |
| Kuwait | 0.85 | 343.89 |
| Haiti | 0.45 | 343.47 |
| Angola | 0.45 | 342.15 |
| Saint Vincent and the Grenadines | 0.64 | 336.64 |
| Tuvalu | 0.58 | 334.92 |
| Armenia | 0.70 | 332.75 |
| Lao People's Democratic Republic | 0.49 | 317.20 |
| United Republic of Tanzania | 0.45 | 316.02 |
| Sudan | 0.54 | 310.63 |
| Liberia | 0.35 | 310.31 |
| Saint Lucia | 0.67 | 304.10 |
| Democratic Republic of the Congo | 0.38 | 303.58 |
| Tonga | 0.63 | 292.45 |
| Malawi | 0.38 | 289.72 |
| Mozambique | 0.33 | 288.66 |
| Mongolia | 0.62 | 286.47 |
| Comoros | 0.48 | 285.29 |
| Djibouti | 0.49 | 283.81 |
| Niger | 0.17 | 283.05 |
| Jordan | 0.73 | 279.42 |
| Sierra Leone | 0.36 | 278.50 |
| Guam | 0.80 | 275.45 |
| Jamaica | 0.68 | 275.35 |
| Guinea-Bissau | 0.35 | 271.33 |
| Guinea | 0.34 | 269.08 |
| Congo | 0.58 | 268.89 |
| Burundi | 0.29 | 256.85 |
| Marshall Islands | 0.57 | 249.19 |
| Dominica | 0.75 | 236.98 |
| Seychelles | 0.73 | 236.07 |
| Egypt | 0.61 | 233.95 |
| Trinidad and Tobago | 0.77 | 233.15 |
| Bahrain | 0.75 | 230.80 |
| Chad | 0.24 | 229.22 |
| Democratic People's Republic of Korea | 0.57 | 222.60 |
| Zimbabwe | 0.47 | 201.29 |
| Kyrgyzstan | 0.60 | 198.87 |
| Mauritius | 0.72 | 188.78 |
| Vanuatu | 0.47 | 187.14 |
| Namibia | 0.62 | 184.82 |
| Botswana | 0.64 | 181.63 |
| Fiji | 0.68 | 157.81 |
| Madagascar | 0.40 | 130.56 |
| Pakistan | 0.50 | 130.49 |
| Central African Republic | 0.31 | 129.91 |
| South Sudan | 0.28 | 115.48 |
| Lesotho | 0.51 | 106.25 |
| Tajikistan | 0.54 | 106.14 |
| Eswatini | 0.59 | 105.62 |
| Azerbaijan | 0.69 | 104.99 |
| South Africa | 0.68 | 101.91 |
| Uzbekistan | 0.66 | 100.56 |
| Bangladesh | 0.49 | 73.08 |
| Turkmenistan | 0.68 | 68.90 |
| Somalia | 0.08 | 67.53 |
| Kiribati | 0.53 | 33.75 |

Abbreviations: ASPR, age-standardized prevalence rate; HFs, Hip fractures; SDI, sociodemographic index.

**Table S8c. Frontier analysis results of ASYR for HFs (≥55 years) across 204 countries and territories**

| **Location** | **SDI** | **Effective difference** |
| --- | --- | --- |
| Greenland | 0.83 | 535.15 |
| Andorra | 0.87 | 478.72 |
| Belgium | 0.85 | 388.41 |
| Switzerland | 0.93 | 375.87 |
| Norway | 0.92 | 365.83 |
| Finland | 0.86 | 353.93 |
| Netherlands | 0.89 | 353.92 |
| France | 0.84 | 346.92 |
| Australia | 0.84 | 326.29 |
| Canada | 0.87 | 312.34 |
| Luxembourg | 0.88 | 291.74 |
| New Zealand | 0.85 | 283.04 |
| Austria | 0.85 | 276.80 |
| Sweden | 0.89 | 274.86 |
| India | 0.58 | 268.01 |
| Cyprus | 0.84 | 267.54 |
| Germany | 0.90 | 253.99 |
| Republic of Korea | 0.89 | 252.69 |
| United States of America | 0.86 | 246.82 |
| Slovenia | 0.84 | 246.64 |
| Croatia | 0.80 | 241.97 |
| Italy | 0.81 | 225.12 |
| Denmark | 0.90 | 224.49 |
| Brunei Darussalam | 0.81 | 218.08 |
| Solomon Islands | 0.43 | 211.95 |
| Cuba | 0.67 | 208.68 |
| Iceland | 0.88 | 202.01 |
| United Kingdom | 0.86 | 196.81 |
| Malta | 0.80 | 191.02 |
| Bhutan | 0.47 | 190.87 |
| Cambodia | 0.47 | 189.96 |
| Papua New Guinea | 0.42 | 184.39 |
| Chile | 0.77 | 181.30 |
| Ireland | 0.87 | 181.05 |
| Uruguay | 0.72 | 175.78 |
| Viet Nam | 0.63 | 162.17 |
| San Marino | 0.89 | 160.12 |
| Nepal | 0.43 | 157.49 |
| Spain | 0.77 | 152.04 |
| Hungary | 0.79 | 145.39 |
| Saudi Arabia | 0.82 | 145.07 |
| Czechia | 0.83 | 143.65 |
| Nicaragua | 0.52 | 138.22 |
| Argentina | 0.72 | 134.39 |
| Monaco | 0.91 | 132.08 |
| Myanmar | 0.53 | 131.07 |
| Portugal | 0.74 | 130.95 |
| Palau | 0.75 | 128.32 |
| Guatemala | 0.54 | 126.84 |
| Slovakia | 0.81 | 124.11 |
| Brazil | 0.65 | 121.78 |
| Afghanistan | 0.34 | 115.77 |
| Israel | 0.81 | 113.31 |
| Mexico | 0.66 | 109.13 |
| Sri Lanka | 0.70 | 108.79 |
| Sao Tome and Principe | 0.51 | 107.71 |
| El Salvador | 0.56 | 105.20 |
| Northern Mariana Islands | 0.77 | 103.22 |
| Eritrea | 0.40 | 103.03 |
| Indonesia | 0.66 | 101.47 |
| Japan | 0.87 | 99.50 |
| Guyana | 0.65 | 98.31 |
| Poland | 0.81 | 95.80 |
| Lithuania | 0.86 | 94.84 |
| Timor-Leste | 0.44 | 93.85 |
| Costa Rica | 0.70 | 91.04 |
| Rwanda | 0.44 | 89.80 |
| Libya | 0.73 | 86.41 |
| Mali | 0.27 | 85.97 |
| Burkina Faso | 0.29 | 85.35 |
| Haiti | 0.45 | 84.77 |
| Greece | 0.79 | 84.77 |
| Singapore | 0.86 | 84.20 |
| Morocco | 0.56 | 82.04 |
| Kenya | 0.52 | 81.44 |
| Cameroon | 0.48 | 77.14 |
| Bolivia (Plurinational State of) | 0.60 | 77.00 |
| Gambia | 0.41 | 75.58 |
| Paraguay | 0.64 | 75.12 |
| Oman | 0.77 | 74.84 |
| Ghana | 0.56 | 74.44 |
| Nigeria | 0.50 | 74.38 |
| Togo | 0.41 | 74.27 |
| Senegal | 0.41 | 74.00 |
| Benin | 0.37 | 73.24 |
| Zambia | 0.51 | 72.83 |
| Angola | 0.45 | 72.45 |
| Nauru | 0.63 | 71.83 |
| Ethiopia | 0.36 | 71.20 |
| Uganda | 0.42 | 70.34 |
| China | 0.72 | 70.33 |
| Samoa | 0.59 | 69.56 |
| Gabon | 0.63 | 69.05 |
| North Macedonia | 0.75 | 68.88 |
| Honduras | 0.51 | 68.09 |
| Venezuela (Bolivarian Republic of) | 0.60 | 68.02 |
| Mozambique | 0.33 | 67.62 |
| Guinea-Bissau | 0.35 | 67.25 |
| Micronesia (Federated States of) | 0.59 | 66.86 |
| Coted'Ivoire | 0.43 | 66.56 |
| Ecuador | 0.66 | 65.59 |
| Burundi | 0.29 | 65.43 |
| Yemen | 0.45 | 64.10 |
| Suriname | 0.63 | 63.61 |
| Democratic Republic of the Congo | 0.38 | 63.28 |
| Lao People's Democratic Republic | 0.49 | 62.87 |
| Georgia | 0.73 | 62.79 |
| Mauritania | 0.50 | 61.76 |
| Saint Kitts and Nevis | 0.75 | 61.59 |
| United Republic of Tanzania | 0.45 | 60.58 |
| Comoros | 0.48 | 60.52 |
| Malawi | 0.38 | 60.22 |
| Tuvalu | 0.58 | 58.82 |
| Montenegro | 0.80 | 58.79 |
| Latvia | 0.83 | 58.65 |
| Djibouti | 0.49 | 58.43 |
| Guinea | 0.34 | 58.07 |
| Liberia | 0.35 | 58.01 |
| Turkey | 0.71 | 57.17 |
| Malaysia | 0.74 | 56.90 |
| Philippines | 0.65 | 56.65 |
| Grenada | 0.67 | 56.54 |
| Sierra Leone | 0.36 | 55.83 |
| Bahamas | 0.81 | 55.13 |
| Equatorial Guinea | 0.66 | 54.79 |
| Niger | 0.17 | 54.65 |
| Cabo Verde | 0.53 | 54.14 |
| American Samoa | 0.72 | 53.94 |
| Chad | 0.24 | 53.68 |
| United States Virgin Islands | 0.82 | 53.03 |
| Congo | 0.58 | 52.82 |
| Dominican Republic | 0.62 | 52.14 |
| Belize | 0.61 | 51.58 |
| Marshall Islands | 0.57 | 50.87 |
| Palestine | 0.63 | 50.21 |
| Serbia | 0.79 | 49.37 |
| Niue | 0.73 | 48.14 |
| Maldives | 0.65 | 47.02 |
| Iraq | 0.66 | 46.69 |
| Tokelau | 0.69 | 46.50 |
| Romania | 0.77 | 46.08 |
| Saint Vincent and the Grenadines | 0.64 | 45.49 |
| Russian Federation | 0.81 | 45.48 |
| Central African Republic | 0.31 | 45.39 |
| Bosnia and Herzegovina | 0.72 | 45.23 |
| Bulgaria | 0.77 | 44.89 |
| Ukraine | 0.76 | 44.37 |
| Zimbabwe | 0.47 | 44.35 |
| Sudan | 0.54 | 42.89 |
| Puerto Rico | 0.83 | 42.34 |
| Belarus | 0.78 | 41.72 |
| Thailand | 0.68 | 41.44 |
| Tonga | 0.63 | 39.21 |
| Mongolia | 0.62 | 39.20 |
| Vanuatu | 0.47 | 37.81 |
| South Sudan | 0.28 | 37.52 |
| Estonia | 0.84 | 35.54 |
| Algeria | 0.66 | 35.10 |
| Antigua and Barbuda | 0.75 | 33.75 |
| Colombia | 0.66 | 33.72 |
| Iran (Islamic Republic of) | 0.70 | 32.72 |
| United Arab Emirates | 0.85 | 32.14 |
| Dominica | 0.75 | 31.86 |
| Republic of Moldova | 0.73 | 31.59 |
| Saint Lucia | 0.67 | 31.52 |
| Kazakhstan | 0.73 | 31.21 |
| Botswana | 0.64 | 30.60 |
| Namibia | 0.62 | 30.22 |
| Barbados | 0.75 | 29.70 |
| Peru | 0.66 | 28.96 |
| Madagascar | 0.40 | 28.52 |
| Lesotho | 0.51 | 27.85 |
| Panama | 0.71 | 26.65 |
| Albania | 0.71 | 26.62 |
| Syrian Arab Republic | 0.62 | 26.27 |
| Tunisia | 0.68 | 25.53 |
| Fiji | 0.68 | 24.66 |
| Jamaica | 0.68 | 24.10 |
| Pakistan | 0.50 | 23.18 |
| Qatar | 0.85 | 22.99 |
| Guam | 0.80 | 22.21 |
| Egypt | 0.61 | 21.91 |
| Trinidad and Tobago | 0.77 | 21.34 |
| Cook Islands | 0.78 | 19.78 |
| Seychelles | 0.73 | 19.29 |
| Democratic People's Republic of Korea | 0.57 | 18.99 |
| Eswatini | 0.59 | 18.50 |
| Lebanon | 0.74 | 17.47 |
| Armenia | 0.70 | 17.04 |
| Bermuda | 0.82 | 15.38 |
| Tajikistan | 0.54 | 13.78 |
| Somalia | 0.08 | 13.72 |
| Taiwan (Province of China) | 0.87 | 13.58 |
| Kyrgyzstan | 0.60 | 13.58 |
| Kiribati | 0.53 | 11.21 |
| South Africa | 0.68 | 9.64 |
| Mauritius | 0.72 | 8.34 |
| Uzbekistan | 0.66 | 6.26 |
| Jordan | 0.73 | 6.15 |
| Bangladesh | 0.49 | 1.27 |
| Azerbaijan | 0.69 | 0.98 |
| Turkmenistan | 0.68 | 0.51 |
| Bahrain | 0.75 | 0.00 |
| Kuwait | 0.85 | 0.00 |

Abbreviations: ASYR, age-standardized years lived with disability rate; HFs, Hip fractures; SDI, sociodemographic index.

**Table S9. Etiology analysis results of ASIR, ASPR, and ASYR for HFs (≥55 years)**

| **measure** | **location** | **sex** | **cause** | **year** | **percent (%)** |
| --- | --- | --- | --- | --- | --- |
| ASIR | Global | Both | Falls | 2021 | 91.55 |
| ASIR | Global | Both | Road injuries | 2021 | 3.07 |
| ASIR | Global | Both | Exposure to mechanical forces | 2021 | 2.58 |
| ASIR | Global | Both | Interpersonal violence | 2021 | 1.18 |
| ASIR | Global | Both | Other transport injuries | 2021 | 0.55 |
| ASIR | Global | Both | Animal contact | 2021 | 0.42 |
| ASIR | Global | Both | Drowning | 2021 | 0.18 |
| ASIR | Global | Both | Self-harm | 2021 | 0.11 |
| ASIR | Global | Both | Environmental heat and cold exposure | 2021 | 0.10 |
| ASIR | Global | Both | Fire, heat, and hot substances | 2021 | 0.10 |
| ASIR | Global | Both | Foreign body | 2021 | 0.07 |
| ASIR | Global | Both | Conflict and terrorism | 2021 | 0.04 |
| ASIR | Global | Both | Exposure to forces of nature | 2021 | 0.02 |
| ASIR | Global | Both | Police conflict and executions | 2021 | 0.01 |
| ASIR | Global | Both | Poisonings | 2021 | 0.01 |
| ASIR | Global | Both | Adverse effects of medical treatment | 2021 | 0.00 |
| ASPR | Global | Both | Falls | 2021 | 90.77 |
| ASPR | Global | Both | Road injuries | 2021 | 4.61 |
| ASPR | Global | Both | Exposure to mechanical forces | 2021 | 1.41 |
| ASPR | Global | Both | Other transport injuries | 2021 | 1.26 |
| ASPR | Global | Both | Interpersonal violence | 2021 | 0.64 |
| ASPR | Global | Both | Self-harm | 2021 | 0.36 |
| ASPR | Global | Both | Conflict and terrorism | 2021 | 0.22 |
| ASPR | Global | Both | Foreign body | 2021 | 0.20 |
| ASPR | Global | Both | Environmental heat and cold exposure | 2021 | 0.16 |
| ASPR | Global | Both | Fire, heat, and hot substances | 2021 | 0.12 |
| ASPR | Global | Both | Drowning | 2021 | 0.07 |
| ASPR | Global | Both | Exposure to forces of nature | 2021 | 0.07 |
| ASPR | Global | Both | Animal contact | 2021 | 0.04 |
| ASPR | Global | Both | Police conflict and executions | 2021 | 0.04 |
| ASPR | Global | Both | Poisonings | 2021 | 0.02 |
| ASPR | Global | Both | Adverse effects of medical treatment | 2021 | 0.00 |
| ASYR | Global | Both | Falls | 2021 | 89.47 |
| ASYR | Global | Both | Road injuries | 2021 | 4.95 |
| ASYR | Global | Both | Exposure to mechanical forces | 2021 | 1.85 |
| ASYR | Global | Both | Other transport injuries | 2021 | 1.07 |
| ASYR | Global | Both | Interpersonal violence | 2021 | 0.94 |
| ASYR | Global | Both | Self-harm | 2021 | 0.40 |
| ASYR | Global | Both | Conflict and terrorism | 2021 | 0.36 |
| ASYR | Global | Both | Foreign body | 2021 | 0.22 |
| ASYR | Global | Both | Environmental heat and cold exposure | 2021 | 0.20 |
| ASYR | Global | Both | Fire, heat, and hot substances | 2021 | 0.15 |
| ASYR | Global | Both | Animal contact | 2021 | 0.12 |
| ASYR | Global | Both | Drowning | 2021 | 0.11 |
| ASYR | Global | Both | Exposure to forces of nature | 2021 | 0.11 |
| ASYR | Global | Both | Police conflict and executions | 2021 | 0.04 |
| ASYR | Global | Both | Poisonings | 2021 | 0.02 |
| ASYR | Global | Both | Adverse effects of medical treatment | 2021 | 0.00 |
| ASIR | Global | Both | Falls | 1990 | 87.65 |
| ASIR | Global | Both | Road injuries | 1990 | 4.24 |
| ASIR | Global | Both | Exposure to mechanical forces | 1990 | 3.53 |
| ASIR | Global | Both | Interpersonal violence | 1990 | 2.10 |
| ASIR | Global | Both | Other transport injuries | 1990 | 0.71 |
| ASIR | Global | Both | Animal contact | 1990 | 0.70 |
| ASIR | Global | Both | Drowning | 1990 | 0.22 |
| ASIR | Global | Both | Environmental heat and cold exposure | 1990 | 0.20 |
| ASIR | Global | Both | Fire, heat, and hot substances | 1990 | 0.16 |
| ASIR | Global | Both | Self-harm | 1990 | 0.15 |
| ASIR | Global | Both | Conflict and terrorism | 1990 | 0.13 |
| ASIR | Global | Both | Foreign body | 1990 | 0.09 |
| ASIR | Global | Both | Exposure to forces of nature | 1990 | 0.08 |
| ASIR | Global | Both | Police conflict and executions | 1990 | 0.02 |
| ASIR | Global | Both | Poisonings | 1990 | 0.01 |
| ASIR | Global | Both | Adverse effects of medical treatment | 1990 | 0.00 |
| ASPR | Global | Both | Falls | 1990 | 86.40 |
| ASPR | Global | Both | Road injuries | 1990 | 7.41 |
| ASPR | Global | Both | Exposure to mechanical forces | 1990 | 1.91 |
| ASPR | Global | Both | Other transport injuries | 1990 | 1.54 |
| ASPR | Global | Both | Interpersonal violence | 1990 | 1.14 |
| ASPR | Global | Both | Self-harm | 1990 | 0.50 |
| ASPR | Global | Both | Environmental heat and cold exposure | 1990 | 0.30 |
| ASPR | Global | Both | Foreign body | 1990 | 0.26 |
| ASPR | Global | Both | Fire, heat, and hot substances | 1990 | 0.19 |
| ASPR | Global | Both | Drowning | 1990 | 0.11 |
| ASPR | Global | Both | Conflict and terrorism | 1990 | 0.08 |
| ASPR | Global | Both | Animal contact | 1990 | 0.07 |
| ASPR | Global | Both | Exposure to forces of nature | 1990 | 0.05 |
| ASPR | Global | Both | Poisonings | 1990 | 0.03 |
| ASPR | Global | Both | Police conflict and executions | 1990 | 0.01 |
| ASPR | Global | Both | Adverse effects of medical treatment | 1990 | 0.00 |
| ASYR | Global | Both | Falls | 1990 | 83.27 |
| ASYR | Global | Both | Road injuries | 1990 | 8.60 |
| ASYR | Global | Both | Exposure to mechanical forces | 1990 | 2.57 |
| ASYR | Global | Both | Interpersonal violence | 1990 | 1.74 |
| ASYR | Global | Both | Other transport injuries | 1990 | 1.39 |
| ASYR | Global | Both | Self-harm | 1990 | 0.75 |
| ASYR | Global | Both | Environmental heat and cold exposure | 1990 | 0.41 |
| ASYR | Global | Both | Foreign body | 1990 | 0.36 |
| ASYR | Global | Both | Fire, heat, and hot substances | 1990 | 0.27 |
| ASYR | Global | Both | Drowning | 1990 | 0.19 |
| ASYR | Global | Both | Conflict and terrorism | 1990 | 0.17 |
| ASYR | Global | Both | Animal contact | 1990 | 0.16 |
| ASYR | Global | Both | Exposure to forces of nature | 1990 | 0.09 |
| ASYR | Global | Both | Poisonings | 1990 | 0.03 |
| ASYR | Global | Both | Police conflict and executions | 1990 | 0.01 |
| ASYR | Global | Both | Adverse effects of medical treatment | 1990 | 0.00 |
| ASIR | Southern Sub-Saharan Africa | Both | Falls | 2021 | 50.10 |
| ASIR | Southern Sub-Saharan Africa | Both | Road injuries | 2021 | 17.87 |
| ASIR | Southern Sub-Saharan Africa | Both | Interpersonal violence | 2021 | 15.71 |
| ASIR | Southern Sub-Saharan Africa | Both | Exposure to mechanical forces | 2021 | 10.69 |
| ASIR | Southern Sub-Saharan Africa | Both | Animal contact | 2021 | 1.81 |
| ASIR | Southern Sub-Saharan Africa | Both | Fire, heat, and hot substances | 2021 | 1.08 |
| ASIR | Southern Sub-Saharan Africa | Both | Other transport injuries | 2021 | 0.87 |
| ASIR | Southern Sub-Saharan Africa | Both | Self-harm | 2021 | 0.65 |
| ASIR | Southern Sub-Saharan Africa | Both | Drowning | 2021 | 0.45 |
| ASIR | Southern Sub-Saharan Africa | Both | Environmental heat and cold exposure | 2021 | 0.36 |
| ASIR | Southern Sub-Saharan Africa | Both | Foreign body | 2021 | 0.31 |
| ASIR | Southern Sub-Saharan Africa | Both | Police conflict and executions | 2021 | 0.05 |
| ASIR | Southern Sub-Saharan Africa | Both | Exposure to forces of nature | 2021 | 0.03 |
| ASIR | Southern Sub-Saharan Africa | Both | Conflict and terrorism | 2021 | 0.02 |
| ASIR | Southern Sub-Saharan Africa | Both | Poisonings | 2021 | 0.01 |
| ASIR | Southern Sub-Saharan Africa | Both | Adverse effects of medical treatment | 2021 | 0.00 |
| ASPR | Southern Sub-Saharan Africa | Both | Falls | 2021 | 50.31 |
| ASPR | Southern Sub-Saharan Africa | Both | Road injuries | 2021 | 25.63 |
| ASPR | Southern Sub-Saharan Africa | Both | Interpersonal violence | 2021 | 10.15 |
| ASPR | Southern Sub-Saharan Africa | Both | Exposure to mechanical forces | 2021 | 5.38 |
| ASPR | Southern Sub-Saharan Africa | Both | Self-harm | 2021 | 2.26 |
| ASPR | Southern Sub-Saharan Africa | Both | Other transport injuries | 2021 | 1.97 |
| ASPR | Southern Sub-Saharan Africa | Both | Fire, heat, and hot substances | 2021 | 1.42 |
| ASPR | Southern Sub-Saharan Africa | Both | Foreign body | 2021 | 1.14 |
| ASPR | Southern Sub-Saharan Africa | Both | Environmental heat and cold exposure | 2021 | 0.60 |
| ASPR | Southern Sub-Saharan Africa | Both | Conflict and terrorism | 2021 | 0.33 |
| ASPR | Southern Sub-Saharan Africa | Both | Drowning | 2021 | 0.25 |
| ASPR | Southern Sub-Saharan Africa | Both | Animal contact | 2021 | 0.25 |
| ASPR | Southern Sub-Saharan Africa | Both | Police conflict and executions | 2021 | 0.21 |
| ASPR | Southern Sub-Saharan Africa | Both | Exposure to forces of nature | 2021 | 0.08 |
| ASPR | Southern Sub-Saharan Africa | Both | Poisonings | 2021 | 0.02 |
| ASPR | Southern Sub-Saharan Africa | Both | Adverse effects of medical treatment | 2021 | 0.00 |
| ASYR | Southern Sub-Saharan Africa | Both | Falls | 2021 | 50.30 |
| ASYR | Southern Sub-Saharan Africa | Both | Road injuries | 2021 | 25.16 |
| ASYR | Southern Sub-Saharan Africa | Both | Interpersonal violence | 2021 | 10.39 |
| ASYR | Southern Sub-Saharan Africa | Both | Exposure to mechanical forces | 2021 | 5.72 |
| ASYR | Southern Sub-Saharan Africa | Both | Self-harm | 2021 | 2.23 |
| ASYR | Southern Sub-Saharan Africa | Both | Other transport injuries | 2021 | 1.90 |
| ASYR | Southern Sub-Saharan Africa | Both | Fire, heat, and hot substances | 2021 | 1.40 |
| ASYR | Southern Sub-Saharan Africa | Both | Foreign body | 2021 | 1.09 |
| ASYR | Southern Sub-Saharan Africa | Both | Environmental heat and cold exposure | 2021 | 0.58 |
| ASYR | Southern Sub-Saharan Africa | Both | Animal contact | 2021 | 0.35 |
| ASYR | Southern Sub-Saharan Africa | Both | Conflict and terrorism | 2021 | 0.31 |
| ASYR | Southern Sub-Saharan Africa | Both | Drowning | 2021 | 0.27 |
| ASYR | Southern Sub-Saharan Africa | Both | Police conflict and executions | 2021 | 0.19 |
| ASYR | Southern Sub-Saharan Africa | Both | Exposure to forces of nature | 2021 | 0.09 |
| ASYR | Southern Sub-Saharan Africa | Both | Poisonings | 2021 | 0.02 |
| ASYR | Southern Sub-Saharan Africa | Both | Adverse effects of medical treatment | 2021 | 0.00 |
| ASIR | Southern Sub-Saharan Africa | Both | Falls | 1990 | 48.09 |
| ASIR | Southern Sub-Saharan Africa | Both | Road injuries | 1990 | 17.98 |
| ASIR | Southern Sub-Saharan Africa | Both | Interpersonal violence | 1990 | 16.38 |
| ASIR | Southern Sub-Saharan Africa | Both | Exposure to mechanical forces | 1990 | 11.66 |
| ASIR | Southern Sub-Saharan Africa | Both | Animal contact | 1990 | 2.03 |
| ASIR | Southern Sub-Saharan Africa | Both | Fire, heat, and hot substances | 1990 | 1.05 |
| ASIR | Southern Sub-Saharan Africa | Both | Other transport injuries | 1990 | 0.77 |
| ASIR | Southern Sub-Saharan Africa | Both | Self-harm | 1990 | 0.54 |
| ASIR | Southern Sub-Saharan Africa | Both | Drowning | 1990 | 0.49 |
| ASIR | Southern Sub-Saharan Africa | Both | Environmental heat and cold exposure | 1990 | 0.43 |
| ASIR | Southern Sub-Saharan Africa | Both | Foreign body | 1990 | 0.34 |
| ASIR | Southern Sub-Saharan Africa | Both | Conflict and terrorism | 1990 | 0.20 |
| ASIR | Southern Sub-Saharan Africa | Both | Exposure to forces of nature | 1990 | 0.03 |
| ASIR | Southern Sub-Saharan Africa | Both | Poisonings | 1990 | 0.01 |
| ASIR | Southern Sub-Saharan Africa | Both | Police conflict and executions | 1990 | 0.00 |
| ASIR | Southern Sub-Saharan Africa | Both | Adverse effects of medical treatment | 1990 | 0.00 |
| ASPR | Southern Sub-Saharan Africa | Both | Falls | 1990 | 43.28 |
| ASPR | Southern Sub-Saharan Africa | Both | Road injuries | 1990 | 30.28 |
| ASPR | Southern Sub-Saharan Africa | Both | Interpersonal violence | 1990 | 12.89 |
| ASPR | Southern Sub-Saharan Africa | Both | Exposure to mechanical forces | 1990 | 5.45 |
| ASPR | Southern Sub-Saharan Africa | Both | Self-harm | 1990 | 2.26 |
| ASPR | Southern Sub-Saharan Africa | Both | Other transport injuries | 1990 | 1.64 |
| ASPR | Southern Sub-Saharan Africa | Both | Fire, heat, and hot substances | 1990 | 1.39 |
| ASPR | Southern Sub-Saharan Africa | Both | Foreign body | 1990 | 1.20 |
| ASPR | Southern Sub-Saharan Africa | Both | Environmental heat and cold exposure | 1990 | 0.71 |
| ASPR | Southern Sub-Saharan Africa | Both | Drowning | 1990 | 0.30 |
| ASPR | Southern Sub-Saharan Africa | Both | Conflict and terrorism | 1990 | 0.23 |
| ASPR | Southern Sub-Saharan Africa | Both | Animal contact | 1990 | 0.21 |
| ASPR | Southern Sub-Saharan Africa | Both | Exposure to forces of nature | 1990 | 0.06 |
| ASPR | Southern Sub-Saharan Africa | Both | Police conflict and executions | 1990 | 0.06 |
| ASPR | Southern Sub-Saharan Africa | Both | Poisonings | 1990 | 0.02 |
| ASPR | Southern Sub-Saharan Africa | Both | Adverse effects of medical treatment | 1990 | 0.00 |
| ASYR | Southern Sub-Saharan Africa | Both | Falls | 1990 | 43.00 |
| ASYR | Southern Sub-Saharan Africa | Both | Road injuries | 1990 | 30.25 |
| ASYR | Southern Sub-Saharan Africa | Both | Interpersonal violence | 1990 | 13.04 |
| ASYR | Southern Sub-Saharan Africa | Both | Exposure to mechanical forces | 1990 | 5.62 |
| ASYR | Southern Sub-Saharan Africa | Both | Self-harm | 1990 | 2.25 |
| ASYR | Southern Sub-Saharan Africa | Both | Other transport injuries | 1990 | 1.60 |
| ASYR | Southern Sub-Saharan Africa | Both | Fire, heat, and hot substances | 1990 | 1.39 |
| ASYR | Southern Sub-Saharan Africa | Both | Foreign body | 1990 | 1.18 |
| ASYR | Southern Sub-Saharan Africa | Both | Environmental heat and cold exposure | 1990 | 0.70 |
| ASYR | Southern Sub-Saharan Africa | Both | Drowning | 1990 | 0.31 |
| ASYR | Southern Sub-Saharan Africa | Both | Animal contact | 1990 | 0.26 |
| ASYR | Southern Sub-Saharan Africa | Both | Conflict and terrorism | 1990 | 0.24 |
| ASYR | Southern Sub-Saharan Africa | Both | Exposure to forces of nature | 1990 | 0.06 |
| ASYR | Southern Sub-Saharan Africa | Both | Police conflict and executions | 1990 | 0.06 |
| ASYR | Southern Sub-Saharan Africa | Both | Poisonings | 1990 | 0.02 |
| ASYR | Southern Sub-Saharan Africa | Both | Adverse effects of medical treatment | 1990 | 0.00 |

Abbreviations: ASIR, age-standardized incidence rate; ASPR, age-standardized prevalence rate; ASYR, age-standardized years lived with disability rate; HFs, Hip fractures.
